# Unnatural amino acid substitutions to improve in vivo stability and tumor uptake of ^68^Ga-labeled GRPR-targeted TacBOMB2 derivatives for cancer imaging with positron emission tomography

**DOI:** 10.1186/s41181-024-00241-7

**Published:** 2024-02-02

**Authors:** Lei Wang, Hsiou-Ting Kuo, Zhengxing Zhang, Chengcheng Zhang, Chao-Cheng Chen, Devon Chapple, Ryan Wilson, Nadine Colpo, François Bénard, Kuo-Shyan Lin

**Affiliations:** 1 Department of Molecular Oncology, BC Cancer Research Institute, Vancouver, BC V5Z1L3 Canada; 2 Department of Molecular Imaging and Therapy, BC Cancer, Vancouver, BC V5Z4E6 Canada; 3 Department of Radiology, University of British Columbia, Vancouver, BC V5Z1M9 Canada

**Keywords:** Gastrin-releasing peptide receptor, Agonist, In vivo stability, Positron emission tomography, Pancreas uptake

## Abstract

**Background:**

Overexpressed in various solid tumors, gastrin-releasing peptide receptor (GRPR) is a promising cancer imaging marker and therapeutic target. Although antagonists are preferable for the development of GRPR-targeted radiopharmaceuticals due to potentially fewer side effects, internalization of agonists may lead to longer tumor retention and better treatment efficacy. In this study, we systematically investigated unnatural amino acid substitutions to improve in vivo stability and tumor uptake of a previously reported GRPR-targeted agonist tracer, [^68^Ga]Ga-TacBOMB2 (^68^Ga-DOTA-Pip-D-Phe^6^-Gln^7^-Trp^8^-Ala^9^-Val^10^-Gly^11^-His^12^-Leu^13^-Thz^14^-NH_2_).

**Results:**

Unnatural amino acid substitutions were conducted for Gln^7^, Trp^8^, Ala^9^, Val^10^, Gly^11^ and His^12^, either alone or in combination. Out of 25 unnatural amino acid substitutions, *tert*-Leu^10^ (Tle^10^) and NMe-His^12^ substitutions were identified to be preferable modifications especially in combination. Compared with the previously reported [^68^Ga]Ga-TacBOMB2, the Tle^10^ and NMe-His^12^ derived [^68^Ga]Ga-LW01110 showed retained agonist characteristics and improved GRPR binding affinity (K_i_ = 7.62 vs 1.39 nM), in vivo stability (12.7 vs 89.0% intact tracer in mouse plasma at 15 min post-injection) and tumor uptake (5.95 vs 16.6 %ID/g at 1 h post-injection).

**Conclusions:**

Unnatural amino acid substitution is an effective strategy to improve in vivo stability and tumor uptake of peptide-based radiopharmaceuticals. With excellent tumor uptake and tumor-to-background contrast, [^68^Ga]Ga-LW01110 is promising for detecting GRPR-expressing cancer lesions with PET. Since agonists can lead to internalization upon binding to receptors and foreseeable long tumor retention, our optimized GRPR-targeted sequence, [Tle^10^,NMe-His^12^,Thz^14^]Bombesin(7–14), is a promising template for use for the design of GRPR-targeted radiotherapeutic agents.

**Supplementary Information:**

The online version contains supplementary material available at 10.1186/s41181-024-00241-7.

## Background

Gastrin-releasing peptide receptor (GRPR) is a G protein-coupled receptor, expressed in pancreas, gastrointestinal tract, and central nervous system, and involved in physiological functions such as synaptic plasticity, hormone secretion, and smooth muscle contraction (Jensen et al. 2008; Bitar and Zhu 1993; Weber 2009). Overexpression of GRPR has been reported to induce cancer cell proliferation and facilitate malignant neoplasm development (Jensen et al. 2008; Weber 2009; Cornelio et al. 2007; Hajri et al. 1996; Moody et al. 1996; Preston et al. 1995, 1994; Gugger and Reubi 1999; Markwalder and Reubi 1999; Roesler et al. 2006; Shimoda 1992; Qin et al. 1994). The overexpression of GRPR in various tumors makes it a promising target for the design of targeted radiopharmaceuticals for diagnosis and radioligand therapy of GRPR-expressing cancers.

Two natural ligands, gastrin-releasing peptide (GRP) and bombesin (BBN) show high binding affinity towards GRPR and share the same heptapeptide sequence (Trp-Ala-Val-Gly-His-Leu-Met-NH_2_) at the C-terminus (Erspamer et al. 1972a, 1972b; McDonald et al. 1978). The C-terminal heptapeptide of GRP and BBN has been used as a template for designing GRPR-targeted radiopharmaceuticals for decades (Varvarigou et al. 2004; Baum et al. 2007; Kähkönen et al. 2013; Stoykow et al. 2016; Baratto et al. 2021; Kurth et al. 2020; Nock et al. 2017; Marsouvanidis et al. 2013). The derivatives of GRP and BBN have been radiolabeled for imaging with single photon emission computed tomography (SPECT) and positron emission tomography (PET), and some of them have also been radiolabeled with beta and alpha emitters for radiotherapeutic applications (McDonald et al. 1978; Baum et al. 2007; Kurth et al. 2020; Nock et al. 2017; Minamimoto et al. 2016; Lin et al. 2004). However, the current clinically validated GRPR-targeted radioligands show an extremely high uptake in pancreas (Baum et al. 2007; Kähkönen et al. 2013; Kurth et al. 2020; Nock et al. 2017; Minamimoto et al. 2016), which not only limits the detection of cancer lesions located in or adjacent to pancreas, but also lowers the maximum tolerated dose for targeted radioligand therapy. Our group recently reported ^68^Ga-labeled TacsBOMB2 based on a known pseudopeptide-bond-containing antagonist sequence [Leu^13^ψThz^14^]Bombesin(7–14), which showed significant lower pancreas uptake than the clinically validated [^68^Ga]Ga-RM2 (Wang et al. 2022). Replacing the reduced peptide bond (Leu^13^ψThz^14^) with an amide bond restores the GRPR agonist characterizations and the derived [^68^Ga]Ga-TacBOMB2 retained high GRPR binding affinity and low uptake in mouse pancreas (Wang et al. 2023).

The development of GRPR-targeted radiopharmaceuticals has been focused on using antagonist sequences as targeting vectors because of their potentially higher tumor uptake due to higher in vivo stability (Ghosh et al. 2019) and more binding sites than those available for agonists (Mansi et al. 2009), and/or less short term adverse effects (Chatalic et al. 2016; Mansi et al. 2013). However, agonists can be internalized upon binding to GRPR and lead to a longer tumor retention (Jensen et al. 2008; Mansi et al. 2013; Yang et al. 2011), which might be preferable especially for the development of radiotherapeutic agents. The in vivo instability of GRPR-targeted ligands is caused by enzymatic degradation especially by neutral endopeptidase 24.11 (NEP) (Chatalic et al. 2016; Nock et al. 2014). The reported cleavage sites including His^12^-Leu^13^, Trp^8^-Ala^9^ and Gln^7^-Trp^8^ for AMBA derivatives and Trp^8^-Ala^9^, Ala^9^-Val^10^ and Gln^7^-Trp^8^ for RM2 derivatives (Kähkönen et al. 2013; Linder et al. 2009).

**Fig. 1:**
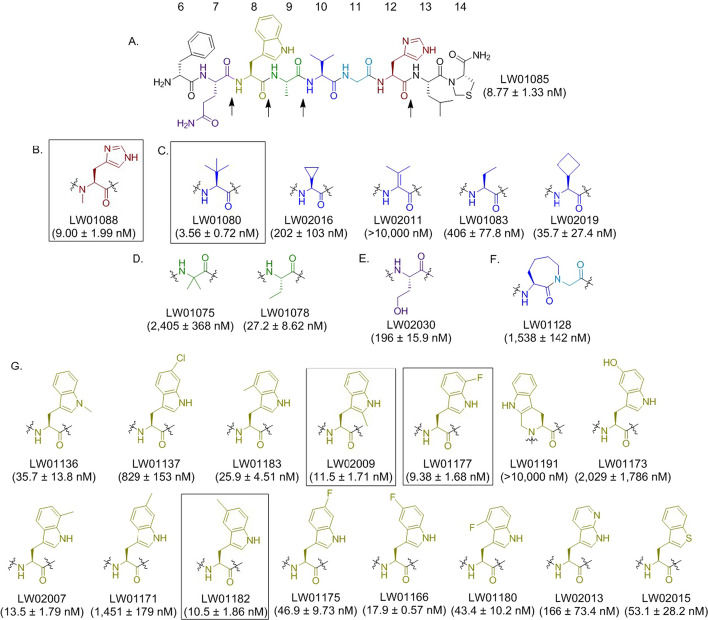
Chemical structures and GRPR binding affinities (K_i_, mean ± SD, n = 3) of **A** LW01085 and its derivatives with an unnatural amino acid substitution at **B** His^12^, **C** Val^10^, **D** Ala^9^, **E** Gln^7^, **F** Val^10^-Gly^11^, and **G** Trp^8^. The potential cleavage sites of LW01085 are pointed by black arrows

We hypothesized that (1) replacing amino acids at the potential cleavage sites of our previously reported GRPR agonist [^68^Ga]Ga-TacBOMB2 ([^68^Ga]Ga-DOTA-Pip-D-Phe^6^-Gln^7^-Trp^8^-Ala^9^-Val^10^-Gly^11^-His^12^-Leu^13^-Thz^14^-NH_2_) with unnatural amino acids can improve in vivo stability and retain the agonist characteristics; and (2) the resulting stabilized [^68^Ga]Ga-TacBOMB2 derivatives can also retain the minimal pancreas uptake characteristics. Thus, in this study we first synthesized the GRPR-targeted sequence of TacBOMB2 (LW01085, D-Phe^6^-Gln^7^-Trp^8^-Ala^9^-Val^10^-Gly^11^-His^12^-Leu^13^-Thz^14^-NH_2_, Fig. 1) and systematically substituted the amino acids (Gln^7^, Trp^8^, Ala^9^, Val^10^, Gly^11^ and His^12^) at its potential cleavage sites with an unnatural amino acid. The derivatives with high GRPR binding affinity were coupled with the DOTA chelator and 4-amino-(1-carboxymethyl)piperidine (Pip) linker. The binding affinities of their Ga-complexed standards were further confirmed by in vitro competition assays, and their agonists characteristics were confirmed by calcium release assays. Finally, the lead candidates were radiolabeled with ^68^Ga and evaluated by PET imaging and ex vivo biodistribution studies using the GRPR-expressing PC-3 prostate cancer model.

## Methods

### Synthesis of GRPR-targeted ligands

Detailed procedures for the synthesis, purification, and characterization of GRPR-targeted ligands and their ^nat^Ga/^68^Ga-complexed analogs are provided in the Supplementary Information (Additional file 1: Figs. S1-S46 and Tables S1-S4).

### Cell culture

The PC-3 cells obtained from ATCC (via Cedarlane, Burlington, Canada) were cultured in RPMI 1640 medium (Life Technologies Corporations, Carlsbad, CA, USA) supplemented with 10% FBS, penicillin (100 U/mL) and streptomycin (100 μg/mL) at 37 °C in a Panasonic Healthcare (Tokyo, Japan) MCO-19AIC humidified incubator containing 5% CO_2_ and 95% air. The cells were confirmed pathogen-free via IMPACT Rodent Pathogen Test (IDEXX BioAnalytics, Columbia, MO, USA). Cells grown to 80–90% confluence were washed with sterile Dulbecco’s phosphate-buffered saline (DPBS, pH 7.4) and collected after 1-min trypsinization. The cell concentration was counted using Moxi mini automated cell counter (ORFLO Technologies, Ketchum, IDUSA).

### In vitro competition binding assays

Inhibition constants (K_i_) of GRPR-targeted ligands to GRPR were measured by in vitro competition binding assay using PC-3 cells and [^125^I-Tyr^4^]Bombesin as the radioligand following previously published procedures (Wang et al. 2022, 2023; Bratanovic et al. 2022). The assays were conducted in triplicate with varied concentrations (10 μM to 1 pM) of tested ligands. Briefly, PC-3 cells were seeded in 24-well poly-D-lysine plates at 2 × 10^5^ cells/well 24–48 h prior to the assay. The growth medium was replaced with 400 μL of reaction medium (RPMI 1640 containing 2 mg/mL BSA, and 20 mM HEPES), then the plates were incubated at 37 °C for 60 min. The tested ligands in 50 μL reaction medium and 50 μL of 0.01 nM [^125^I-Tyr^4^]Bombesin were added into the wells followed by incubation with moderate agitation for 1 h at 37 °C. Cells were gently washed with ice-cold DPBS twice, harvested by trypsinization, and counted for radioactivity on a Perkin Elmer (Waltham, MA, USA) Wizard2 2480 automatic gamma counter. Data were analyzed using nonlinear regression with GraphPad (San Diego, CA, USA) Prism 8 software.

### Fluorometric calcium release assays

Following previously published procedures (Bratanovic et al. 2022; Lau et al. 2019), 5 × 10^4^ PC-3 cells were seeded in 96-well clear bottom black plates 24 h prior to the assay. The growth medium was removed and replaced with a loading buffer containing a calcium-sensitive dye (FLIPR Calcium 6 assay kit from Molecular Devices, San Jose, CA, USA). After incubated at 37 °C for 30 min, the plates were placed in a FlexStation 3 microplate reader (Molecular Devices). Tested ligands (50 nM) or DPBS (negative control) were added to the cells and the fluorescent signals were acquired for 2 min. Agonistic/antagonistic properties of the tested ligands were determined based on the relative fluorescent unit (RFU = max – min) of their generated fluorescent signals.

### LogD_7.4_ measurements

The LogD_7.4_ values of ^68^Ga-labeled tracers were measured using the shake flask method as previously published (Lin et al. 2015). Briefly, aliquots (2 μL) of the ^68^Ga-labeled tracers were added into a 15 mL falcon tube containing 3 mL of n-octanol and 3 mL of DPBS (pH 7.4). The mixture was vortexed for 1 min and then centrifuged at 3,000 rpm for 15 min. Samples of the n-octanol (1 mL) and DPBS (1 mL) layers were collected and measured in a Perkin Elmer Wizard2 2480 automatic gamma counter. LogD_7.4_ was calculated with the following equation: LogD_7.4_ = log_10_[(counts in n-octanol phase)/(counts in DPBS phase)].

### Biodistribution, PET imaging, and in vivo stability studies

PET/CT imaging, biodistribution, and in vivo stability studies were conducted on male NOD.Cg-Rag1^tm1Mom^ Il2rg^tm1Wjl^/SzJ (NRG) mice following previously published procedures (Bratanovic et al. 2022; Lau et al. 2019; Lin et al. 2015; Kuo et al. 2018). The experiments were conducted according to the guidelines established by the Canadian Council on Animal Care and approved by Animal Ethics Committee of the University of British Columbia. The mice were anaesthetized by inhalation of 2.5% isoflurane in 2 mL/min oxygen, and implanted subcutaneously with 5 × 10^6^ PC-3 cells (100 µL; 1:1 PBS/Matrigel) behind the left shoulder. Mice were used for PET/CT imaging and biodistribution studies when the tumor grew to 5–8 mm in diameter over around 4 weeks.

PET imaging experiments were conducted using a Siemens Inveon (Knoxville, TN, USA) micro PET/CT scanner. Each tumor bearing mouse was injected with 3–5 MBq (90.6–166.8 ng) of ^68^Ga-labeled tracer via the lateral caudal tail vein under anaesthesia (2% isoflurane in oxygen). For blocking, the mice were co-injected with 100 μg of [D-Phe^6^,Leu-NHEt^13^,des-Met^14^]Bombesin(6–14). The mice were allowed to recover and roam freely in their cages. After 50 min, the mice were sedated again with 2% isoflurane in oxygen inhalation and positioned on the scanner. A 10-min CT scan was conducted first for localization and attenuation correction after segmentation for reconstructing the PET images, followed by a 10-min static PET imaging acquisition.

For biodistribution studies, the mice were injected with 2–4 MBq (50.2–106.8 ng) of radiotracer as described above. For blocking, the mice were co-injected with [D-Phe^6^,Leu-NHEt^13^,des-Met^14^]Bombesin(6–14) (100 μg). At 1 h post-injection, the mice were anesthetized with 2% isoflurane inhalation, and euthanized by CO_2_ inhalation. Blood was withdrawn by cardiac puncture, and organs/tissues of interest were collected. The collected organs/tissues were rinsed with PBS, blotted dry, weighed, and counted using the automatic gamma counter.

For in vivo stability studies, the ^68^Ga-labeled ligand (6–15 MBq) was injected via the lateral caudal vein into healthy male NRG mice (n = 3). At 15 min post-injection, mice were euthanized, and the urine and blood samples were collected. The plasma was extracted from whole blood samples by the addition of CH_3_CN (500 μL), 1-min vortex, 20-min centrifugation, and the separation of supernatant. The plasma and urine samples were analyzed via radio-HPLC using the conditions for quality control (Additional file 1: Table S4).

### Statistical analysis

Statistical analyses were performed by Student’s *t*-test using the Microsoft (Redmond, WA, USA) Excel software. The comparison of biodistribution data between two tracers was conducted via unpaired two-tailed test. Unpaired one-tailed test was used to compare biodistribution data between blocked/unblocked mice injected with the same tracer. The difference was considered statistically significant when the* p* value was < 0.05.

## Results

### GRPR binding affinities of LW01085 and its derivatives

As shown in Fig. 1, the GRPR binding affinity (K_i_) of LW01085 is 8.77 ± 1.33 nM. Tle^10^ substitution improves binding affinity (LW01080: 3.56 ± 0.72 nM) while NMe-His^12^ (LW01088: 9.00 ± 1.99 nM), 2-Me-Trp^8^ (LW02009: 11.5 ± 1.71 nM), 7-F-Trp^8^ (LW01177: 9.38 ± 1.68 nM) and 5-Me-Trp^8^ (LW01182: 10.5 ± 1.86 nM) substitutions lead to analogs with comparable binding affinities. The other substitutions generate analogs with either slightly reduced (K_i_ = 13.5–53.1 nM for LW02019, LW01078, LW01136, LW01183, LW02007, LW01175, LW01166, LW01180 and LW02015) or greatly reduced binding affinities (K_i_ > 150 nM for LW02016, LW02011, LW01083, LW01075, LW02030, LW01128, LW01137, LW01191, LW01173, LW01171 and LW02013).

### GRPR binding affinities of Ga-TacBOMB2 derivatives

**Fig. 2:**
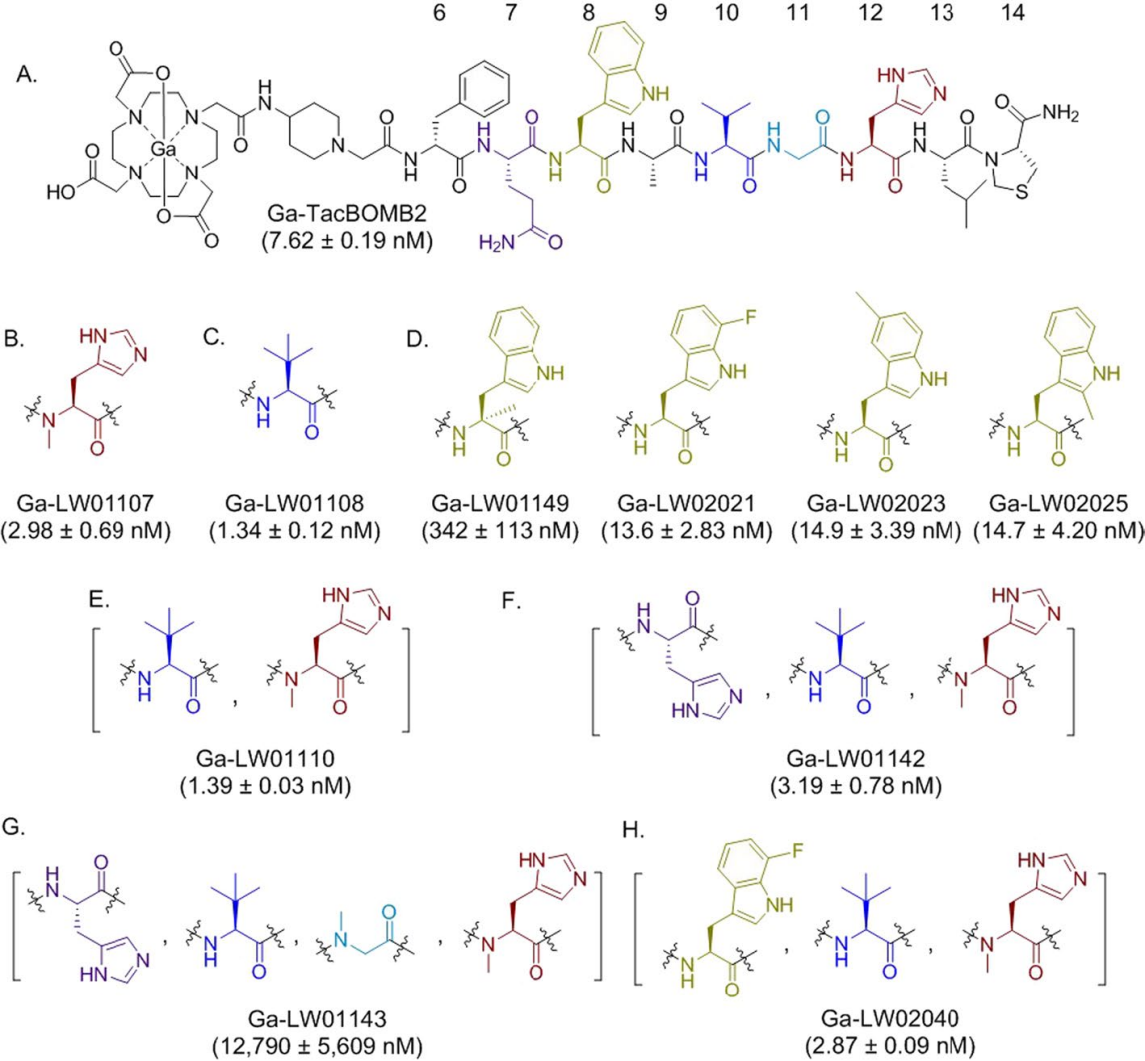
Chemical structures and GRPR binding affinities (K_i_, mean ± SD, n = 3) of **A** Ga-TacBOMB2 and its derivatives with an unnatural amino acid substitution at **B** His^12^, **C** Val^10^, **D** Trp^8^, **E** Val^10^ and His^12^, **F** Gln^7^, Val^10^ and His^12^, **G** Gln^7^, Val^10^, Gly^11^ and His^12^, and **H** Trp^8^, Val^10^ and His^12^

As shown in Fig. 2 and Additional file 1: Figs. S47–S48, compared with the previously reported Ga-TacBOMB2 (K_i_ = 7.62 ± 0.19 nM) (Wang et al. 2023), the NMe-His^12^ and Tle^10^ substitutions, either alone, combined or combined with an additional His^7^ or 7-F-Trp^8^ substitution generate analogs with an enhanced binding affinity (Ga-LW01107: 2.98 ± 0.69 nM; Ga-LW01108: 1.34 ± 0.12 nM; Ga-LW01110: 1.39 ± 0.03 nM; Ga-LW01142: 3.19 ± 0.78 nM; Ga-LW02040: 2.87 ± 0.09 nM). The substitutions of 7-F-Trp^8^, 5-Me-Trp^8^ and 2-Me-Trp^8^ lead to Ga-LW02021, Ga-LW02023 and Ga-LW02025, respectively, with a slightly reduced binding affinity (K_i_ = 13.6–14.9 nM). The derivatives containing an αMe-Trp^8^ (Ga-LW01149) or NMe-Gly^11^ substitution (Ga-LW01143) have poor binding affinities (K_i_ > 300 nM).

### Confirmation of agonist characteristics of Ga-TacBOMB2 derivatives

**Fig. 3:**
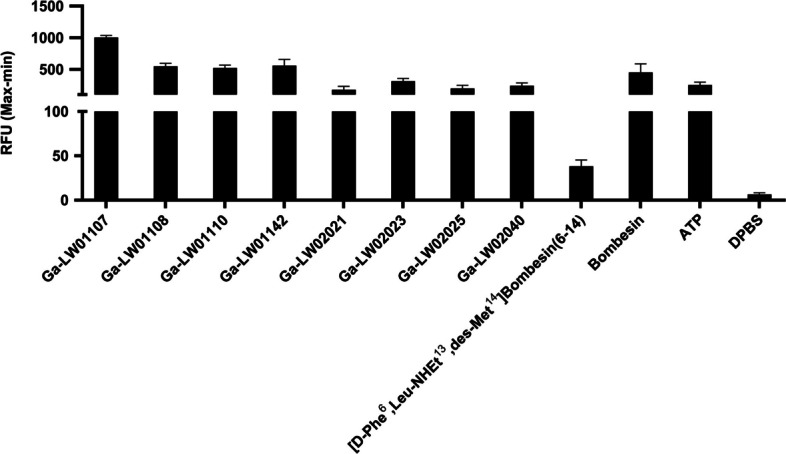
Intracellular calcium efflux in PC-3 cells induced by GRPR-targeted ligands. Cells were incubated with DPBS or 50 nM of Ga-complexed GRPR-targeted ligand, [D-Phe^6^,Leu-NHEt^13^,des-Met^14^]Bombesin(6–14), bombesin, or ATP

To confirm the agonist characteristics of Ga-TacBOMB2 derivatives, calcium release assays were conducted using PC-3 cells. As shown in Fig. 3, Ga-LW01107, Ga-LW01108, Ga-LW01110, Ga-LW01142, Ga-LW02021, Ga-LW02023, Ga-LW02025, and Ga-LW02040 induced Ca^2+^ efflux corresponding to 1,004 ± 32.0, 549 ± 46.7, 521 ± 43.7, 559 ± 96.2, 178 ± 53.7, 312 ± 45.9, 197 ± 50.3, and 242 ± 44.1 relative fluorescence units (RFUs), respectively. The RFUs for the blank control (DPBS), antagonist control ([D-Phe^6^,Leu-NHEt^13^,des-Met^14^]Bombesin(6–14), and positive controls (ATP and bombesin) are 6.57 ± 1.66, 38.2 ± 7.20, 253 ± 46.5 and 450 ± 136, respectively. Therefore, all tested Ga-TacBOMB2 derivatives are confirmed to be GRPR agonists as they induced comparable or higher calcium release than the positive control ATP.

### PET imaging and biodistribution

**Fig. 4:**
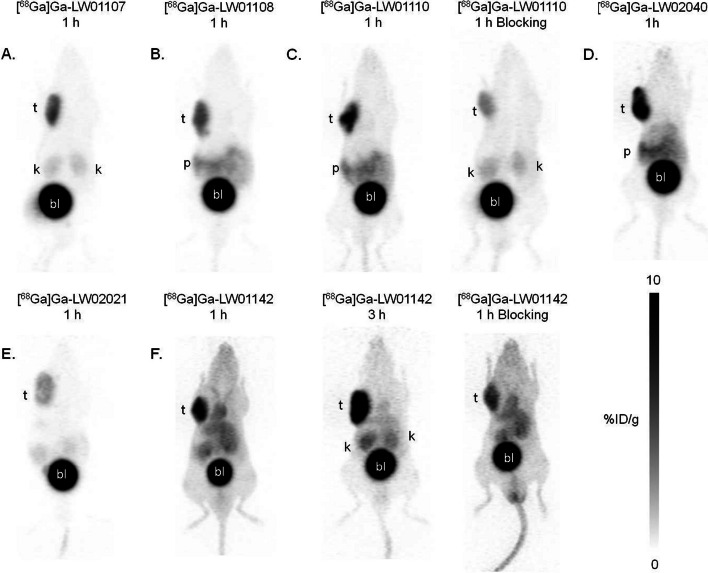
Representative PET images of **A** [^68^Ga]Ga-LW01107, **B** [^68^Ga]Ga-LW01108, **C** [^68^Ga]Ga-LW01110, **D** [^68^Ga]Ga-LW02040, **E** [^68^Ga]Ga-LW02021 and **F** [^68^Ga]Ga-LW01142 in mice bearing PC-3 tumor xenografts. Blocking study was performed by co-injection with 100 μg of [D-Phe^6^,Leu-NHEt^13^,des-Met^14^]Bombesin(6–14). t: tumor; k: kidney; p: pancreas; bl: urinary bladder

The capability of ^68^Ga-labeled TacBOMB2 derivatives to target GRPR in vivo was evaluated by PET imaging and biodistribution studies in mice bearing GRPR-expressing PC-3 tumor xenografts. As shown in Fig. 4, all ^68^ Ga-labeled tracers enabled visualization of PC-3 tumors with good tumor-to-background contrasts. These tracers were excreted mainly via the renal pathway and had only low to moderate uptake in pancreas. Higher tumor uptake was observed by using [^68^Ga]Ga-LW01110, [^68^Ga]Ga-LW02040 and [^68^Ga]Ga-LW01142, followed by [^68^Ga]Ga-LW01107 and [^68^Ga]Ga-LW01108, and [^68^Ga]Ga-LW02021 had the lowest tumor uptake. [^68^Ga]Ga-LW01142 which showed high blood retention at 1 h post-injection was further evaluated at 3 h post-injection (Fig. 4F). The tumor uptake of [^68^Ga]-LW01142 increased further at 3 h post-injection, leading to an enhanced tumor-to-background contrast. Co-injection of [D-Phe^6^,Leu-NHEt^13^,des-Met^14^]Bombesin(6–14) reduced tumor uptake of both [^68^Ga]Ga-LW01110 and [^68^Ga]Ga-LW01142 at 1 h post- injection (Figs. 4C and F).

The biodistribution data of ^68^Ga-labeled GRPR-targeted tracers in PC-3 tumor-bearing mice obtained at 1 h post-injection are provided in Additional file 1: Table S5, and the previously reported data obtained from [^68^Ga]Ga-TacBOMB2, [^68^Ga]Ga-RM2 and [^68^Ga]Ga-AMBA are included for comparison (Wang et al. 2022, 2023). In consistent with the observations from PET images, all ^68^Ga-labeled LW01107, LW01108, LW01110, LW01142, LW02021, and LW02040 had significantly lower uptake in pancreas (0.39 ± 0.03, 9.32 ± 1.97, 8.99 ± 1.54, 4.40 ± 0.27, 1.22 ± 0.18 and 11.7 ± 0.47 %ID/g, respectively) than [^68^Ga]Ga-RM2 (41.9 ± 10.1 %ID/g) and [^68^Ga]Ga-AMBA (62.4 ± 4.26 %ID/g). [^68^Ga]Ga-LW01110 showed the highest tumor uptake (16.6 ± 1.60 %ID/g), followed by [^68^Ga]Ga-LW02040 (12.3 ± 2.14 %ID/g) and [^68^Ga]Ga-LW01142 (11.4 ± 1.22 %ID/g). [^68^Ga]Ga-LW01110 also had the best tumor-to-organ uptake ratios (134 ± 16.7, 119 ± 22.6, 24.7 ± 4.17 and 5.10 ± 0.39 for tumor-to-bone, tumor-to-muscle, tumor-to-blood and tumor-to-kidney, respectively). With the lowest uptake in pancreas (0.39 ± 0.03 %ID/g), [^68^Ga]Ga-LW01107 showed the highest tumor-to-pancreas uptake ratio (17.9 ± 1.10).

**Fig. 5:**
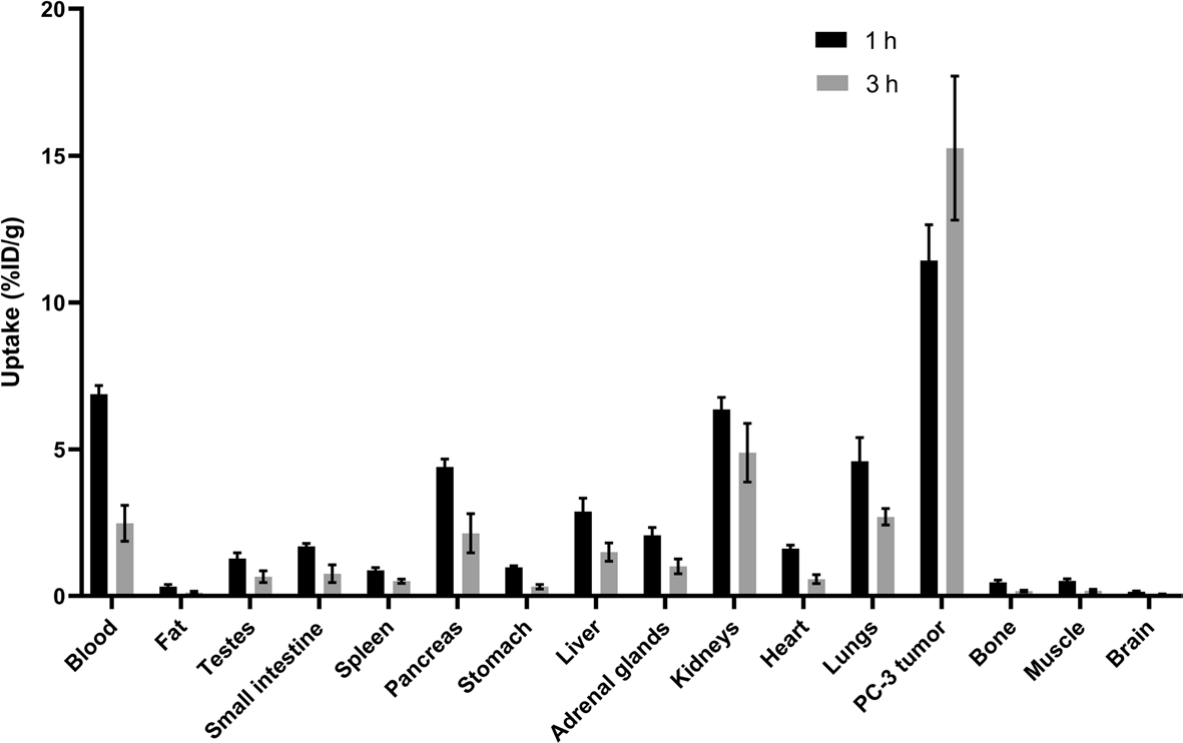
Uptake (mean ± SD, n = 4) of [^68^Ga]Ga-LW01142 at 1 and 3 h post-injection in PC-3 tumor-bearing mice

As [^68^Ga]Ga-LW01142 had high blood pool uptake at 1 h post-injection (6.88 ± 0.29 %ID/g), its biodistribution was further evaluated at 3 h post-injection. The tumor uptake increased (11.4 ± 1.22 to 15.3 ± 2.45 %ID/g) and the uptake in other organs/tissues decreased at 3 h post-injection (Figs. 4F and 5; Additional file 1: Table S6), leading to enhanced tumor-to-background contrast ratios. The tumor-to-bone, tumor-to-muscle, tumor-to-blood, tumor-to-kidney, and tumor-to-pancreas ratios of [^68^Ga]Ga-LW01142 at 3 h post-injection were 91.6 ± 12.2, 86.6 ± 25.1, 6.40 ± 1.70, 3.17 ± 0.46 and 7.36 ± 1.17, respectively.

Blocking studies of [^68^Ga]Ga-LW01110 and [^68^Ga]Ga-LW01142 were conducted at 1 h post-injection (Additional file 1: Table S6). The results showed that co-injection of [D-Phe^6^,Leu-NHEt^13^,des-Met^14^]Bombesin(6–14) reduced their average tumor uptake values by 44% and 31%, respectively. The average pancreas uptake values of [^68^Ga]Ga-LW01110 and [^68^Ga]Ga-LW01142 were also reduced by 42% and 30%, respectively.

### LogD_7.4_ measurement and in vivo stability

LogD_7.4_ measurements were conducted for the ^68^Ga-labeled tracers, and as shown in Table 1, these ^68^Ga-labeled tracers are highly hydrophilic with average LogD_7.4_ values in the range of -3.10 to -1.81. In vivo stability studies showed that, compared with the previously reported [^68^Ga]Ga-TacBOMB2 (12.7 ± 2.93% intact at 15 min post-injection), Tle^10^ ([^68^Ga]Ga-LW01108) and NMe-His^12^ ([^68^Ga]Ga-LW01107) substitutions increased the intact tracer fraction in mouse plasma to 35.3 ± 0.93 and 66.2 ± 12.4%, respectively (Additional file 1: Figs. S49-S50 and Table 1). Further improvement was obtained by combining both Tle^10^ and NMe-His^12^ as ≥ 89% intact was observed for [^68^Ga]Ga-LW01110, [^68^Ga]Ga-LW01142 and [^68^Ga]Ga-LW02040 (Additional file 1: Figs. S51-S53 and Table 1). No intact tracer was detected in mouse urine samples for all the tested GRPR-targeted ligands (Additional file 1: Figs. S49-S53).

**Table 1:** LogD_7.4_ values and in vivo stability of GRPR-targeted tracers

Tracer	LogD_7.4_ (*n* = 3)	Intact fraction (%) in plasma at 15 min post-injection
[^68^Ga]Ga-TacBOMB2	− 3.21 ± 0.04	12.7 ± 2.93
[^68^Ga]Ga-LW01107	− 3.10 ± 0.05	66.2 ± 12.4
[^68^Ga]Ga-LW01108	− 3.10 ± 0.02	35.3 ± 0.93
[^68^Ga]Ga-LW01110	− 2.83 ± 0.08	89.0 ± 2.17
[^68^Ga]Ga-LW01142	− 1.81 ± 0.02	91.2 ± 5.30
[^68^Ga]Ga-LW02021	− 2.46 ± 0.09	–
[^68^Ga]Ga-LW02040	− 2.48 ± 0.12	92.9 ± 2.30
[^68^Ga]Ga-RM2	− 2.76 ± 0.03	71.9 ± 10.4
[^68^Ga]Ga-AMBA	− 3.66 ± 0.03	39.4 ± 10.8

Data are presented as mean ± SD (*n* = 3). The data of [^68^Ga]Ga-TacBOMB2, [^68^Ga]Ga-RM2 and [^68^Ga]Ga-AMBA have been reported previously (Wang et al. 2022, 2023) and are included here for comparison

## Discussion

In this study, we first compared LW01085 (D-Phe-[Thz^14^]Bombesin(7–14), the pharmacophore of our previously reported [^68^Ga]Ga-TacBOMB2) with 25 derivatives with an unnatural amino acid substitution at the potential cleavage sites (Fig. 1). In vitro competition binding assays showed that NMe-His^12^ substitution (LW01088) is tolerable, which is consistent with a previous report by Horwell, et al. (1996) that Ac-Bombesin(7–14) and Ac-[NMe-His^12^]Bombesin have similar GRPR binding affinities (K_i_ = 0.7 vs 0.4 nM). In addition, we discovered that 2-Me-Trp^8^ (LW02009), 7-F-Trp^8^ (LW01177), 5-Me-Trp^8^ (LW01182) and Tle^10^ (LW01080) substitutions also led to derivatives with enhanced or comparable GRPR binding affinities. Therefore, these unnatural amino acid substitutions were selected and subsequently compared/combined with two reported unnatural amino acid substitutions (αMe-Trp^8^ and NMe-Gly^11^) for the design of Ga-DOTA-complexed Pip-linker-containing GRPR-targeted ligands (Fig. 2).

Despite being popularly used for the design of GRPR-targeted antagonist ligands (Richter et al. 2016; Sah et al. 2015), NMe-Gly^11^ substitution was reported to cause > 30-fold reduction in GRPR binding affinity for an agonist sequence (K_i_ = 0.7 vs 25 nM for Ac-Bombesin(7–14) and Ac-[NMe-Gly^11^]Bombesin(7–14), respectively) (Horwell et al. 1996). Consistent with the previous report, we also observed a dramatic reduction in GRPR binding affinity with the NMe-Gly^11^ substitution (Figs. 2F and G, K_i_ = 3.19 vs 12,790 nM for Ga-LW01142 and Ga-LW01143, respectively). αMe-Trp^8^ substitution has been successfully used by the Wester group for the design of potent and stable radiolabeled GRPR-targeted antagonists derived from RM2 (Guenther et al. 2022). However, for agonist Ga-TacBOMB2, αMe-Trp^8^ substitution in Ga-LW01149 caused significant loss of binding affinity (K_i_ = 7.62 vs 342 nM, Figs. 2A and D). Our data suggest that GRPR agonists and antagonists might bind to the receptors in different configurations as αMe-Trp^8^ and NMe-Gly^11^ substitutions which are commonly used for antagonist modifications hinder the binding of agonists to the receptors.

His^7^ (the amino acid at the corresponding position in GRP), 2-Me-Trp^8^, 7-F-Trp^8^, 5-Me-Trp^8^, Tle^10^ and NMe-His^12^ substitutions, either alone or in combination, still led to GRPR-targeted ligands with potent binding affinities (K_i_ = 1.34–14.9 nM, Fig. 2). This suggests that compared with the targeted peptide sequences presented in Fig. 1, the addition of Ga-DOTA complex and the Pip linker does not affect their binding affinity. Similarly, based on the results of calcium release assays (Fig. 3), His^7^, 2-Me-Trp^8^, 7-F-Trp^8^, 5-Me-Trp^8^, Tle^10^ and NMe-His^12^ substitutions, either alone or in combination, do not change their agonist characteristics.

Subsequently, we radiolabeled potent candidates and evaluated their potential for prostate cancer imaging. As shown in Fig. 4, all ^68^Ga-labeled tracers were successfully used to visualize PC-3 tumor xenografts in their PET images, confirming good GRPR targeting capabilities of these tracers. A lower tumor uptake was observed for [^68^Ga]Ga-LW02021, which could be due to its relatively weaker GRPR binding affinity compared with those of others (K_i_ = 13.6 vs 1.34 – 3.19 nM, Fig. 2). The clearance of these tracers was mainly via the renal pathway, consistent with the highly hydrophilic nature of these tracers (LogD_7.4_ values ≤ -1.81). A higher blood retention was observed for [^68^Ga]Ga-LW01142 at 1 h post-injection, which could be due to its relatively higher lipophilicity than other tracers (LogD_7.4_ = -1.81 vs -2.46 – -3.10, Table 1).

Ex vivo biodistribution studies were also conducted to better quantify uptake in tumors and normal organs/tissues. As shown in Additional file 1: Table S5, except [^68^Ga]Ga-LW02021 (3.08 ± 0.48 %ID/g at 1 h post-injection), all other evaluated tracers had comparable or improved uptake in PC-3 tumors when compared to that of the previously reported [^68^Ga]Ga-TacBOMB2 (5.95 ± 0.05 %ID/g). Notably, while Tle^10^ substitution led to [^68^Ga]Ga-LW01108 (5.90 ± 0.68 %ID/g) with a comparable tumor uptake, NMe-His^12^ led to [^68^Ga]Ga-LW01107 (7.05 ± 0.71 %ID/g) with an improved tumor uptake. Most importantly, the combination of both Tle^10^ and NMe-His^12^ with and without an addition substitution (His^7^ or 7-F-Trp^8^) led to [^68^Ga]Ga-LW01110 (16.6 ± 1.66 %ID/g), [^68^Ga]Ga-LW01142 (11.4 ± 1.22 %ID/g) and [^68^Ga]Ga-LW02040 (12.3 ± 2.14 %ID/g) with a further improved tumor uptake. Since ^68^Ga-labeled LW01107, LW01108, LW01110, LW01142 and LW02040 have comparable GRPR binding affinities (K_i_ = 1.34–3.19 nM), we suspected that the greatly improved tumor uptake for tracers with at least both Tle^10^ and NMe-His^12^ substitutions could be mainly due to their improved in vivo stability.

In vivo stability studies were subsequently conducted to verify our hypothesis. As shown in Table 1, compared with the previously reported [^68^Ga]Ga-TacBOMB2 (12.7 ± 2.93% intact tracer at 15 min post-injection), Tle^10^ and NMe-His^12^ substitutions led to [^68^Ga]Ga-LW01108 (35.3 ± 0.93% intact) and [^68^Ga]Ga-LW01107 (66.2 ± 12.4% intact) with an improved in vivo stability. Combination of at least both Tle^10^ and NMe-His^12^ substitutions further led to [^68^Ga]Ga-LW01110, [^68^Ga]Ga-LW01142 and [^68^Ga]Ga-LW02040 with an average ≥ 89% intact tracer at 15 min post-injection. These data are consistent with the trend of their tumor uptake observed from the ex vivo biodistribution studies: [^68^Ga]Ga-TacBOMB2 ≈ [^68^Ga]Ga-LW01108 < [^68^Ga]Ga-LW01107 < [^68^Ga]Ga-LW01110, [^68^Ga]Ga-LW01142 and [^68^Ga]Ga-LW02040. In addition, our in vivo stability data also suggest that His^12^-Leu^13^ is the major cleavage site of GRPR-targeted ligands, followed by Ala^9^-Val^10^, and then Gln^7^-Trp^8^/Trp^8^-Ala^9^. This is also consistent with the fact that most of reported GRPR-targeted radioligands had modifications to avoid the cleavage at His^12^-Leu^13^ such as using Sta^13^ substitution for the RM2 derivatives (Kähkönen et al. 2013; Mansi et al. 2011) and Leu^13^ψThz^14^ in our previously reported TacsBOMB derivatives (Wang et al. 2022). Contrary to the improved stability observed in plasma, no intact tracer was detected in urine samples even for ligands with both Tle^10^ and NMe-His^12^ substitutions (Additional file 1: Figs. S49–S53). This is due to the facts that GRPR-targeted ligands are cleaved mainly by NEP and kidneys have the highest NEP expression level (Jiang et al. 2004). Therefore, GRPR-targeted tracers which remain intact in plasma are completely metabolized by NEP in kidneys before being excreted into the urinary bladder.

Compared with the clinically validated [^68^Ga]Ga-RM2 and [^68^Ga]Ga-AMBA (Wang et al. 2022, 2023), our stabilized tracers ([^68^Ga]Ga-LW01110, [^68^Ga]Ga-LW01142 and [^68^Ga]Ga-LW02040) have not only higher tumor uptake, but also comparable or even higher tumor-to-background uptake ratios (Additional file 1: Tables S2–S3). Most importantly, they also have a much lower pancreas uptake than [^68^Ga]Ga-RM2 and [^68^Ga]Ga-AMBA (4.40–11.7 vs 41.9–62.4%ID/g at 1 h post-injection). Therefore, these tracers are expected to have a higher sensitivity for detecting cancer lesions in or adjacent to the pancreas, and can achieve better treatment efficacy and cause less damage to the pancreas when radiolabeled with an α- or β-emitter for radiotherapeutic applications.

## Conclusions

We systematically replaced the amino acids (Gln^7^, Trp^8^, Ala^9^, Val^10^, Gly^11^ and His^12^) at potential cleavage sites of the previously reported sequence of [^68^Ga]Ga-TacBOMB2, and identified that Tle^10^ and NMe-His^12^ substitutions, either alone or in combination, led to derivatives with comparable/enhanced GRPR binding affinities. In vivo stability and ex vivo biodistribution studies confirmed the improved stability resulted from unnatural amino acid substitutions, which further led to enhanced tumor uptake. With both Tle^10^ and NMe-His^12^ substitutions, the top candidate [^68^Ga]Ga-LW01110 has higher in vivo stability, tumor uptake and tumor-to-background uptake ratios than clinically validated [^68^Ga]Ga-RM2 and [^68^Ga]Ga-AMBA, and is promising for use for detecting GRPR-expressing tumors with PET. Due to the observed lower pancreas uptake and foreseeable longer tumor retention as being agonists, our optimized sequence, [Tle^10^,NMe-His^12^,Thz^14^]Bombesin(7–14), is a promising template for use for the design of GRPR-targeted radiotherapeutic agents.

### Supplementary Information

**Additional file 1. **Supplementary Information for the GRPR-targeted radioligands with unnatural amino acid substitutions.

## Data Availability

The datasets used and/or analysed during the current study are available from the corresponding author on reasonable request.

## Abbreviations

BBN: Bombesin
DPBS: Dulbecco’s phosphate-buffered saline
GRP: Gastrin-releasing peptide
GRPR: Gastrin-releasing peptide receptor
K_i_: Inhibition constant
NEP: Neutral endopeptidase 24.11
NRG mice: NOD.Cg-Rag1^tm1Mom^ Il2rg^tm1Wjl^/SzJ mice
PET: Positron emission tomography
RFU: Relative fluorescent unit
Tle: *tert*-Leu
