# Supplementary material for: Unnatural amino acid substitutions to improve in vivo stability and tumor uptake of 68Ga-labeled GRPR-targeted TacBOMB2 derivatives for cancer imaging with positron emission tomography
Source: EJNMMI Radiopharm Chem. 2024 Feb 2;9:8. doi: 10.1186/s41181-024-00241-7 (PMC10837402; doi:10.1186/s41181-024-00241-7)
Supplement: Supplementary file 1 — Additional file 1. Supplementary Information for the GRPR-targeted radioligands with unnatural amino acid substitutions. [file 41181_2024_241_MOESM1_ESM.doc]

**SUPPLEMENTARY INFORMATION**

**Unnatural amino acid** **substitutions to improve *in vivo* stability and tumor uptake of ^68^Ga-labeled GRPR-targeted TacBOMB2 derivatives for cancer imaging with positron emission tomography**

Lei Wang^1^, Hsiou-Ting Kuo^1^, Zhengxing Zhang^1^, Chengcheng Zhang^1^, Chao-Cheng Chen^1^, Devon Chapple^1^, Ryan Wilson^1^, Nadine Colpo^1,2^, François Bénard^1,2,3^, Kuo-Shyan Lin*^,1,2,3^

^1^Department of Molecular Oncology, BC Cancer Research Institute, Vancouver, BC V5Z1L3, Canada

^2^Department of Molecular Imaging and Therapy, BC Cancer, Vancouver, BC V5Z4E6, Canada

^3^Department of Radiology, University of British Columbia, Vancouver, BC V5Z1M9, Canada

*Corresponding Author:

Kuo-Shyan Lin. Address: 675 West 10^th^ Avenue, Rm 4-123, Vancouver, BC V5Z1L3, Canada.

Phone: 1-604-675-8208; E-mail: [klin@bccrc.ca](mailto:klin@bccrc.ca)

**General methods**

All chemicals and solvents were purchased from commercial sources and used without further purification. Synthesis of GRPR-targeted peptides was constructed on solid phase using an AAPPTec (Louisville, KY, USA) Endeavor 90 peptide synthesizer. Purification and quality control of peptides and their ^nat^Ga/^68^Ga-complexed analogs were performed on Agilent (Santa Clara, CA, USA) HPLC systems equipped with a model 1200 quaternary pump, a model 1200 UV absorbance detector (220 nm), and a Bioscan (Washington, DC, USA) NaI scintillation detector. The operation of Agilent HPLC systems was controlled using the Agilent ChemStation software. A semi-preparative column (Luna C18, 5 µm, 250 × 10 mm) and an analytical column (Luna C18, 5 µm, 250 × 4.6 mm) purchased from Phenomenex (Torrance, CA, USA) were used for purification and quality control, respectively. The HPLC eluates containing the desired product were collected and lyophilized with a Labconco (Kansas City, MO, USA) FreeZone 4.5 Plus freeze-drier. MS analyses were conducted with a Waters (Milford, MA, USA) Acquity QDa mass spectrometer equipped with a 2489 UV/Vis detector and an e2695 Separations module. C18 Sep-Pak cartridges (1 cm^3^, 50 mg) were purchased from Waters (Milford, MA, USA). ^68^Ga was eluted from an ITM Medical Isotopes GmbH (Munich, Germany) generator, and purified according to the previously published procedures using a DGA resin column from Eichrom Technologies LLC (Lisle, IL, USA) [1]. The radioactivity of ^68^Ga-labeled peptides was measured using a Capintec (Ramsey, NJ, USA) CRC®-25R/W dose calibrator and the radioactivity of mouse tissues collected from biodistribution studies were counted using a Perkin Elmer (Waltham, MA, USA) Wizard2 2480 automatic gamma counter.

**General procedures for the synthesis of GRPR-targeted peptides**

All GRPR-targeted peptides were synthesized on solid phase using Fmoc peptide chemistry. Rink Amide MBHA resin (0.05 mmol, 0.125g) was treated with 20% piperidine in *N*,*N*-dimethylformamide (DMF) to remove Fmoc protecting group. Fmoc-protected amino acids (3 eq.) and Fmoc-4-amino-(1-carboxymethyl)piperidine (3 eq.) were pre-activated with HATU (3 eq.), HOAt (3 eq.), and *N*,*N*-diisopropylethylamine (DIEA, 9 eq.) and then sequentially coupled to the resin. For DOTA coupling, DOTA(*t*Bu)_3_ (5 eq.) pre-activated with HATU (5 eq.) and DIEA (25 eq.) was coupled to the *N*-terminus. The peptides were deprotected and simultaneously cleaved from the resin with a mixture of trifluoroacetic acid (TFA, 81.5%), triisopropylsilane (TIS 1.0%), water (5%), 2,2′-(ethylenedioxy)diethanethiol (DODT, 2.5%), thioanisole (5%), and phenol (5%) for 2 h at room temperature. The cleaved peptides were filtered and then precipitated by the addition of cold diethyl ether. The crude peptides were collected by centrifugation and purified with HPLC (semi-preparative column; flow rate: 4.5 mL/min). The eluates containing the desired peptides were collected and lyophilized. The purities of all GRPR-targeted peptides were ≥ 95% as determined by HPLC (analytical column, flow rate: 2.0 mL/min, Tables S1 and S2 ).

Table S1: Peptide sequences and purities of GRPR-targeted peptides. The substituted unnatural amino acids are in bold.

| Name | Sequence | Purity (%) |
| --- | --- | --- |
| LW01085 | D-Phe-Gln-Trp-Ala-Val-Gly-His-Leu-Thz-NH_2_ | 99 |
| LW01088 | D-Phe-Gln-Trp-Ala-Val-Gly-**NMe-His**-Leu-Thz-NH_2_ | 98 |
| LW01080 | D-Phe-Gln-Trp-Ala-**Tle**-Gly-His-Leu-Thz-NH_2_ | 99 |
| LW02016 | D-Phe-Gln-Trp-Ala-**cyclopropylglycine**-Gly-His-Leu-Thz-NH_2_ | 98 |
| LW02011 | D-Phe-Gln-Trp-Ala-**2,3-dehydro-Val**-Gly-His-Leu-Thz-NH_2_ | 99 |
| LW01083 | D-Phe-Gln-Trp-Ala-**2-Abu-**Gly-His-Leu-Thz-NH_2_ | 96 |
| LW02019 | D-Phe-Gln-Trp-Ala-**cyclobutaneacetic acid**-Gly-His-Leu-Thz-NH_2_ | 98 |
| LW01075 | D-Phe-Gln-Trp-**Aib**-Val-Gly-His-Leu-Thz-NH_2_ | 99 |
| LW01078 | D-Phe-Gln-Trp-**2-Abu**-Val-Gly-His-Leu-Thz-NH_2_ | 99 |
| LW02030 | D-Phe-**Hse**-Trp-Ala-Val-Gly-His-Leu-Thz-NH_2_ | 98 |
| LW01128 | D-Phe-Gln-Trp-Ala-**3**-**amino**-**1**-**carboxymethylcarprolactame**-His-Leu-Thz-NH_2_ | 95 |
| LW01136 | D-Phe-Gln-**Trp(Me)**-Ala-Val-Gly-His-Leu-Thz-NH_2_ | 99 |
| LW01137 | D-Phe-Gln-**6-Cl-Trp**-Ala-Val-Gly-His-Leu-Thz-NH_2_ | 98 |
| LW01183 | D-Phe-Gln-**4-Me-Trp**-Ala-Val-Gly-His-Leu-Thz-NH_2_ | 99 |
| LW02009 | D-Phe-Gln-**2-Me-Trp**-Ala-Val-Gly-His-Leu-Thz-NH_2_ | 99 |
| LW01177 | D-Phe-Gln-**7-F-Trp**-Ala-Val-Gly-His-Leu-Thz-NH_2_ | 98 |
| LW01191 | D-Phe-Gln-**D-Tpi**-Ala-Val-Gly-His-Leu-Thz-NH_2_ | 96 |
| LW01173 | D-Phe-Gln-**5-OH-Trp**-Ala-Val-Gly-His-Leu-Thz-NH_2_ | 99 |
| LW02007 | D-Phe-Gln-**7-Me-Trp**-Ala-Val-Gly-His-Leu-Thz-NH_2_ | 99 |
| LW01171 | D-Phe-Gln-**6-Me-Trp**-Ala-Val-Gly-His-Leu-Thz-NH_2_ | 99 |
| LW01182 | D-Phe-Gln-**5-Me-Trp**-Ala-Val-Gly-His-Leu-Thz-NH_2_ | 98 |
| LW01175 | D-Phe-Gln-**6-F-Trp**-Ala-Val-Gly-His-Leu-Thz-NH_2_ | 99 |
| LW01166 | D-Phe-Gln-**5-F-Trp**-Ala-Val-Gly-His-Leu-Thz-NH_2_ | 99 |
| LW01180 | D-Phe-Gln-**4-F-Trp**-Ala-Val-Gly-His-Leu-Thz-NH_2_ | 96 |
| LW02013 | D-Phe-Gln-**7-Aza-Trp**-Ala-Val-Gly-His-Leu-Thz-NH_2_ | 99 |
| LW02015 | D-Phe-Gln-**Bta**-Ala-Val-Gly-His-Leu-Thz-NH_2_ | 96 |

Table S2: Peptide sequences and purities of DOTA-conjugated GRPR-targeted peptides. The substituted unnatural amino acids are in bold.

| Name | Sequence | Purity (%) |
| --- | --- | --- |
| LW01107 | DOTA-Pip-D-Phe-Gln-Trp-Ala-Val-Gly-**NMe-His**-Leu-Thz-NH_2_ | 95 |
| LW01108 | DOTA-Pip-D-Phe-Gln-Trp-Ala-**Tle**-Gly-His-Leu-Thz-NH_2_ | 99 |
| LW01149 | DOTA-Pip- D-Phe-Gln-**α-Me-Trp**-Ala-Val-Gly-His-Leu-Thz-NH_2_ | 98 |
| LW02021 | DOTA-Pip-D-Phe-Gln-**7-F-Trp**-Ala-Val-Gly-His-Leu-Thz-NH_2_ | 98 |
| LW02023 | DOTA-Pip-D-Phe-Gln-**5-Me-Trp**-Ala-Val-Gly-His-Leu-Thz-NH_2_ | 98 |
| LW02025 | DOTA-Pip-D-Phe-Gln-**2-Me-Trp**-Ala-Val-Gly-His-Leu-Thz-NH_2_ | 99 |
| LW01110 | DOTA-Pip-D-Phe-Gln-Trp-Ala-**Tle**-Gly-**NMe-His**-Leu-Thz-NH_2_ | 97 |
| LW01142 | DOTA-Pip-D-Phe-His-Trp-Ala-**Tle**-Gly-**NMe-His**-Leu-Thz-NH_2_ | 99 |
| LW01143 | DOTA-Pip-D-Phe-His-Trp-Ala-**Tle**-**NMe-Gly**-**NMe-His**-Leu-Thz-NH_2_ | 99 |
| LW02040 | DOTA-Pip-D-Phe-Gln-**7-F-Trp**-Ala-**Tle**-Gly-**NMe-His**-Leu-Thz-NH_2_ | 97 |

Synthesis of LW01085

Following general procedures, LW01085 was obtained in 41% yield after HPLC purification using 23% CH_3_CN and 0.1% TFA in H_2_O (retention time = 16.0 min).


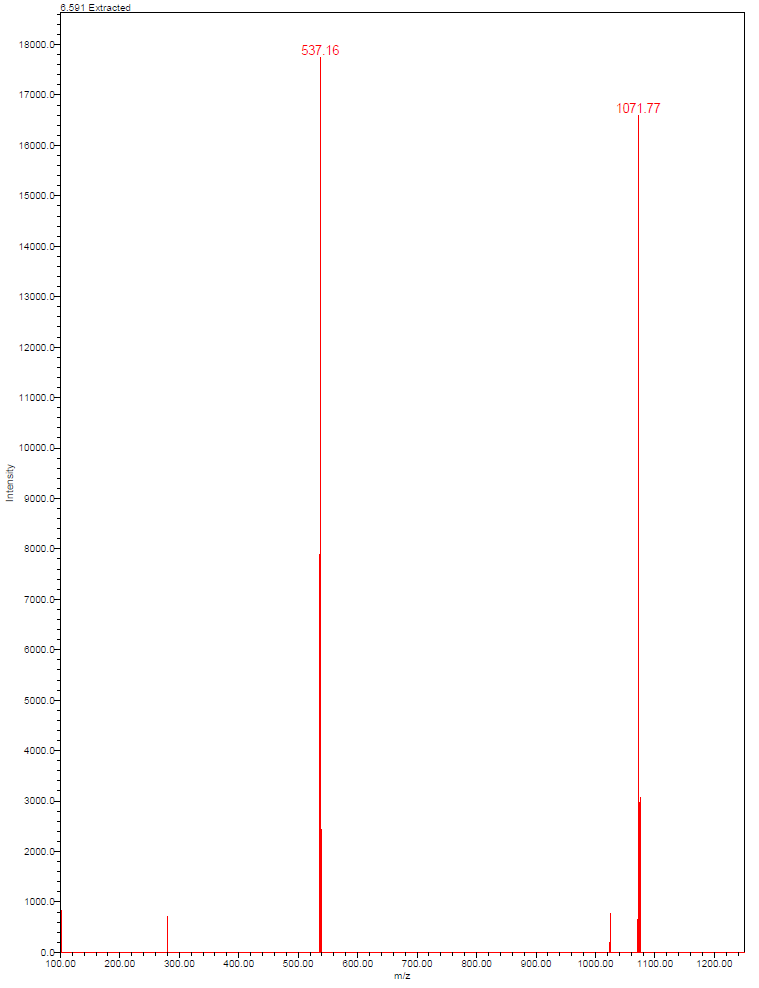


Figure S1: The MS spectrum of LW01085: calculated [M+H]^+^ (m/z) 1071.5; found 1071.8.

Synthesis of LW01088

Following general procedures, LW01088 was obtained in 29% yield after HPLC purification using 23% CH_3_CN and 0.1% TFA in H_2_O (retention time = 17.4 min).


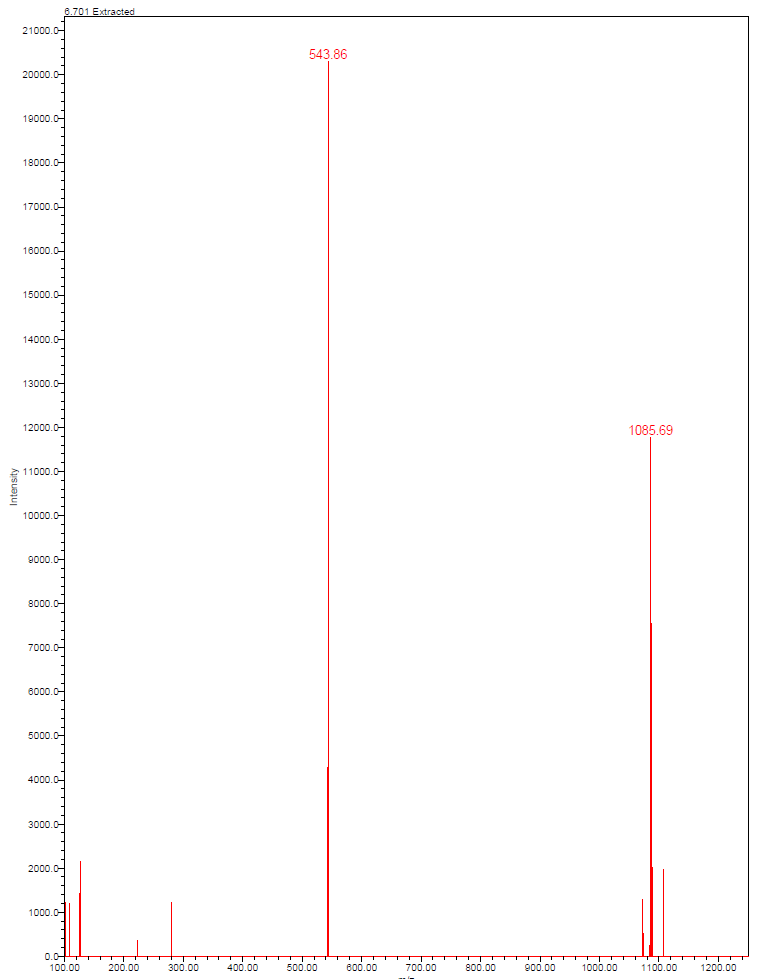


Figure S2: The MS spectrum of LW01088: calculated [M+H]^+^ (m/z) 1085.5; found 1085.7.

Synthesis of LW01080

Following general procedures, LW01080 was obtained in 26% yield after HPLC purification using 26% CH_3_CN and 0.1% TFA in H_2_O (retention time = 9.0 min).


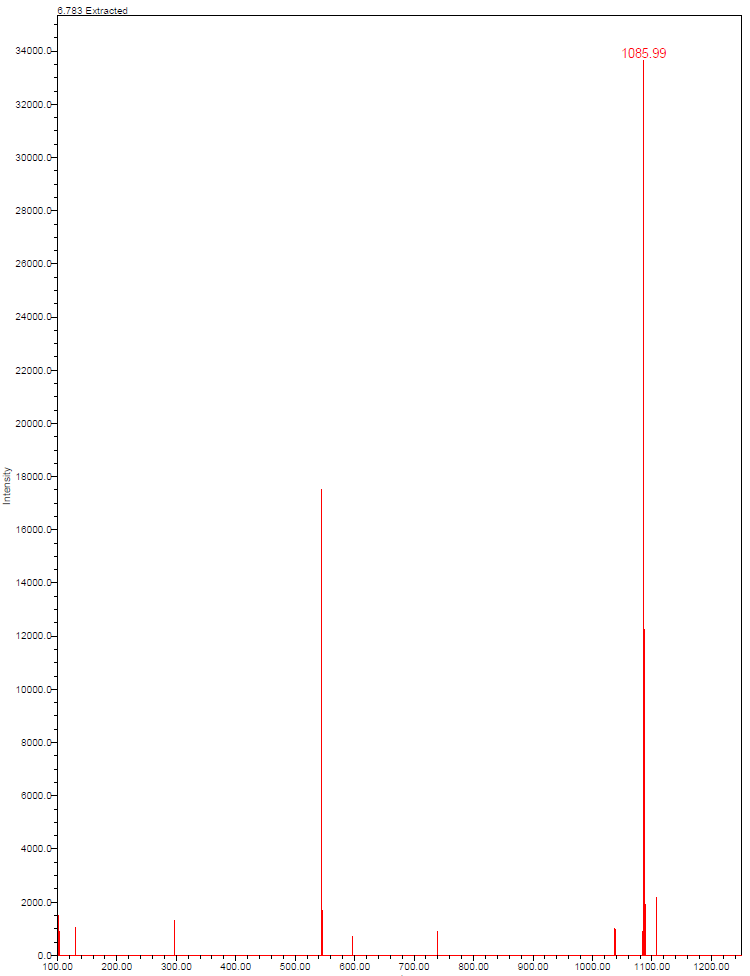


Figure S3: The MS spectrum of LW01080: calculated [M+H]^+^ (m/z) 1985.5; found 1986.0.

Synthesis of LW02016

Following general procedures, LW02016 was obtained in 11% yield after HPLC purification using 25% CH_3_CN and 0.1% TFA in H_2_O (retention time = 8.4 min).


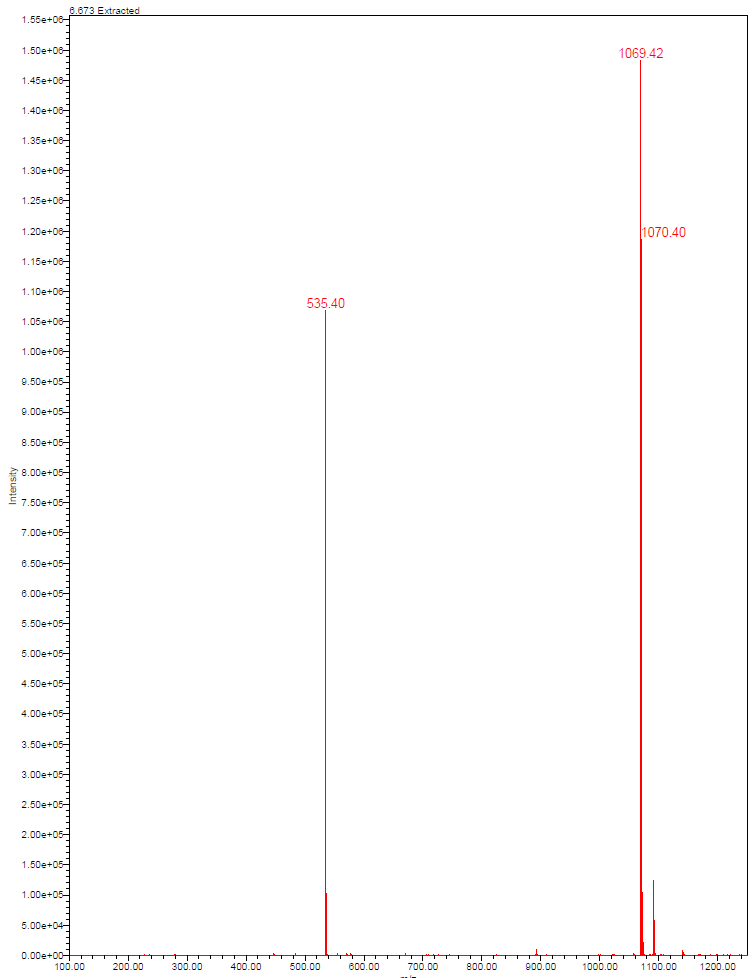


Figure S4: The MS spectrum of LW02016: calculated [M+H]^+^ (m/z) 1069.5; found 1069.4.

Synthesis of LW02011

Following general procedures, LW02011 was obtained in 5.0% yield after HPLC purification using 25% CH_3_CN and 0.1% TFA in H_2_O (retention time = 9.0 min).


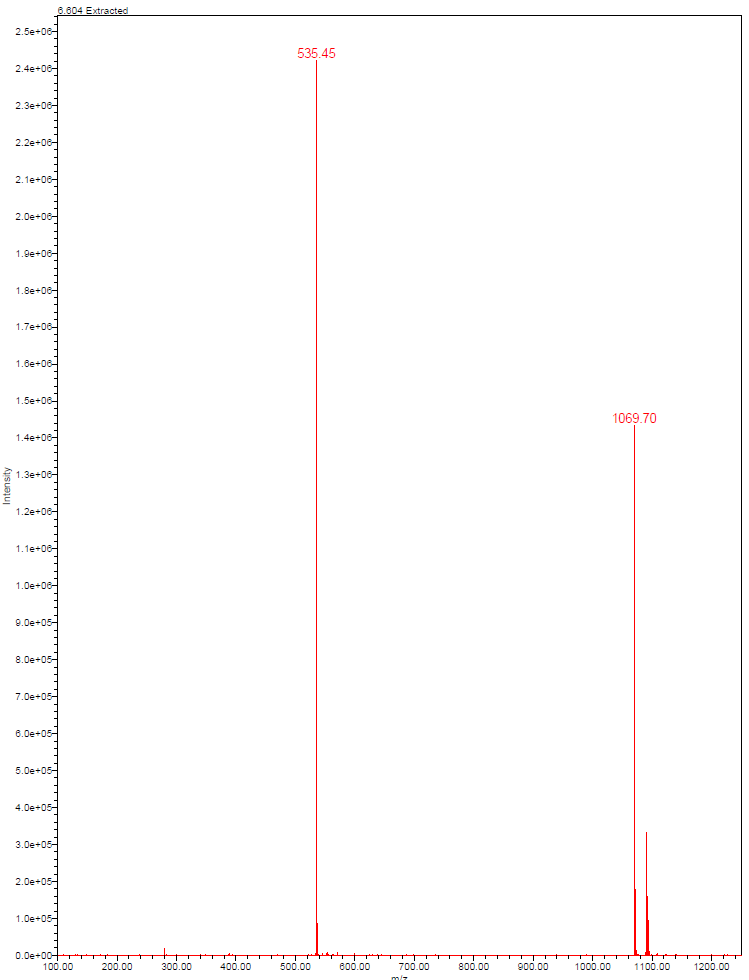


Figure S5: The MS spectrum of LW02011: calculated [M+H]^+^ (m/z) 1069.5; found 1069.7.

Synthesis of LW01083

Following general procedures, LW01083 was obtained in 46% yield after HPLC purification using 22% CH_3_CN and 0.1% TFA in H_2_O (retention time = 12.7 min).


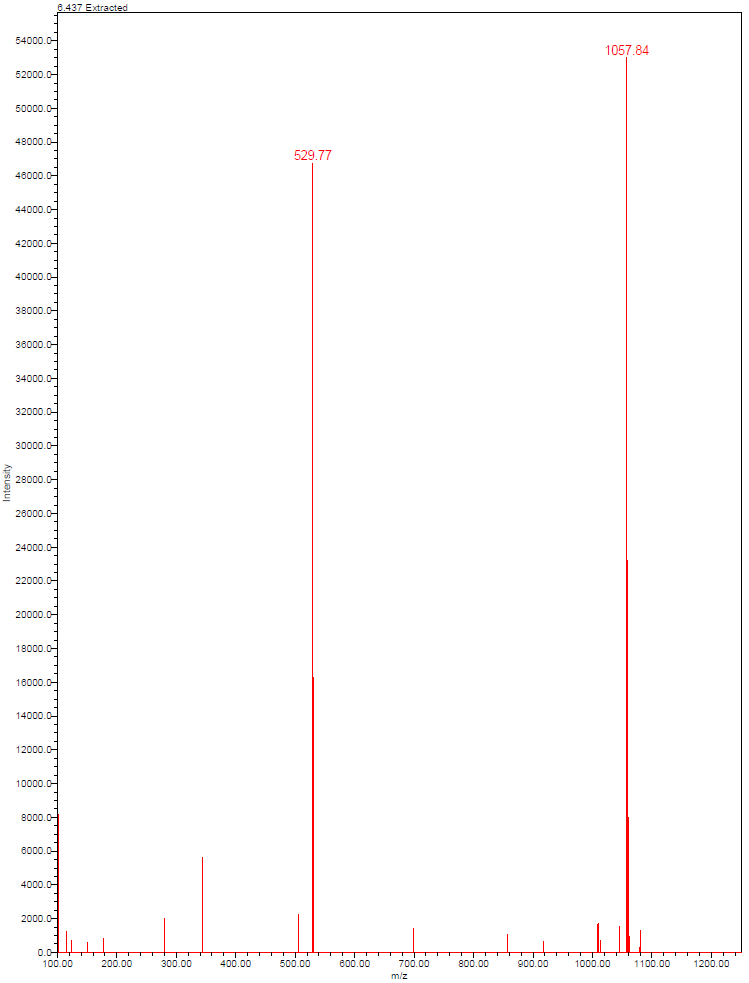


Figure S6: The MS spectrum of LW01083: calculated [M+H]^+^ (m/z) 1057.5; found 1057.8.

Synthesis of LW02019

Following general procedures, LW02019 was obtained in 15% yield after HPLC purification using 26% CH_3_CN and 0.1% TFA in H_2_O (retention time = 9.8 min).


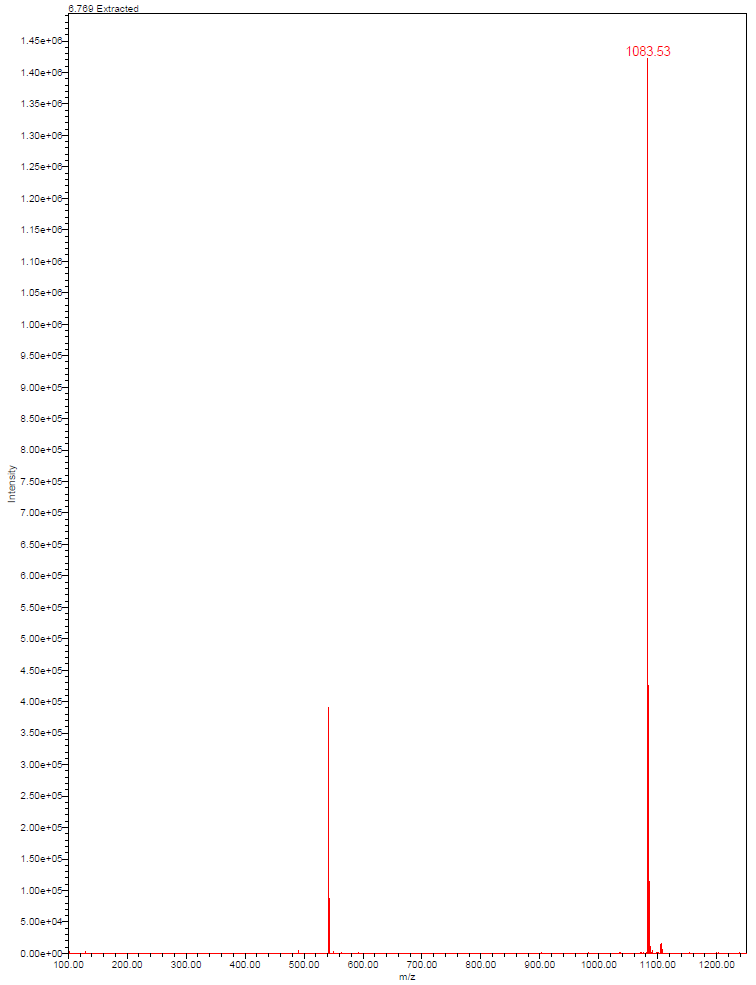


Figure S7: The MS spectrum of LW02019: calculated [M+H]^+^ (m/z) 1083.5; found 1083.5.

Synthesis of LW01075

Following general procedures, LW01075 was obtained in 13% yield after HPLC purification using 25% CH_3_CN and 0.1% TFA in H_2_O (retention time = 11.6 min).


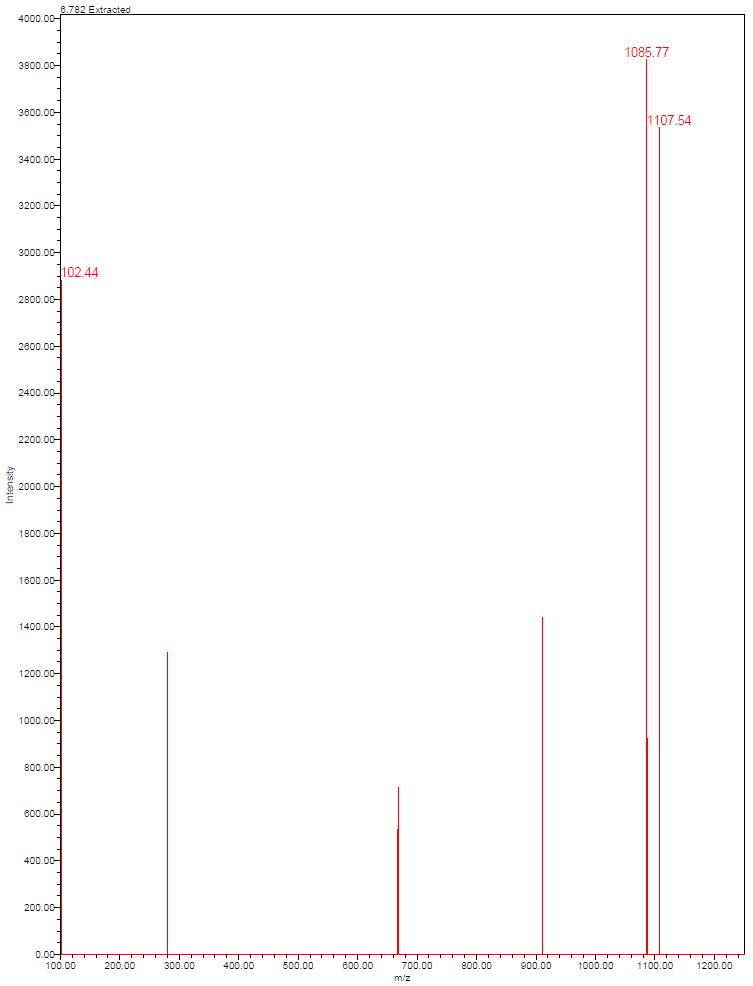


Figure S8: The MS spectrum of LW01075: calculated [M+H]^+^ (m/z) 1085.5; found 1085.8.

Synthesis of LW01078

Following general procedures, LW01078 was obtained in 27% yield after HPLC purification using 25% CH_3_CN and 0.1% TFA in H_2_O (retention time = 10.8 min).


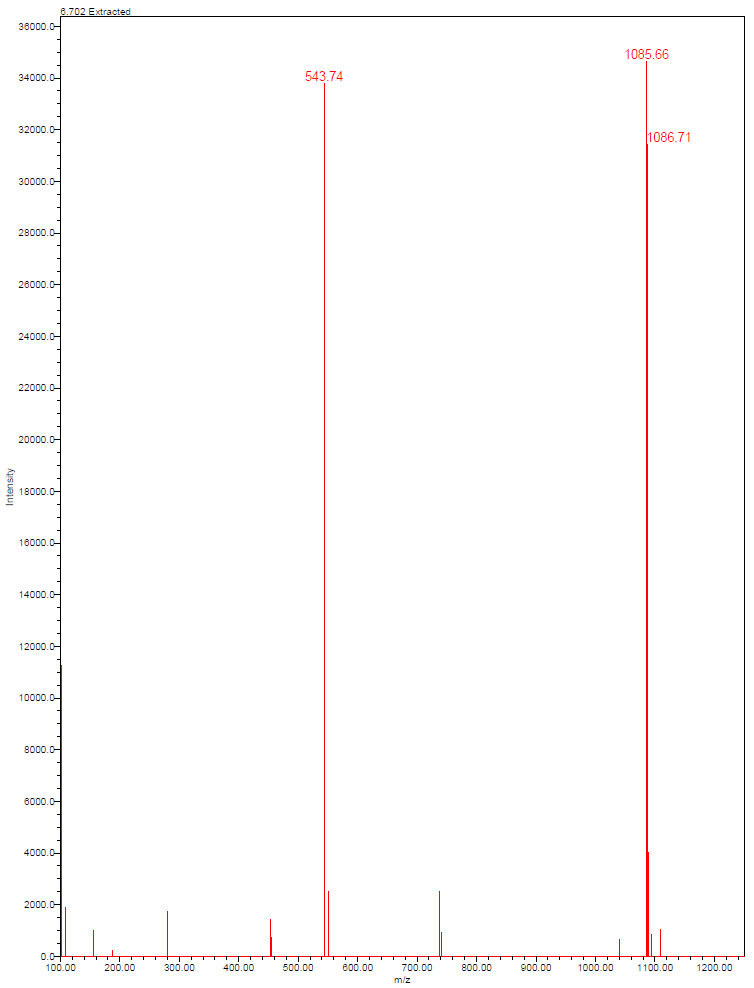


Figure S9: The MS spectrum of LW01078: calculated [M+H]^+^ (m/z) 1085.5; found 1085.7.

Synthesis of LW02030

Following general procedures, LW02030 was obtained in 2.2% yield after HPLC purification using 25% CH_3_CN and 0.1% TFA in H_2_O (retention time = 12.2 min).


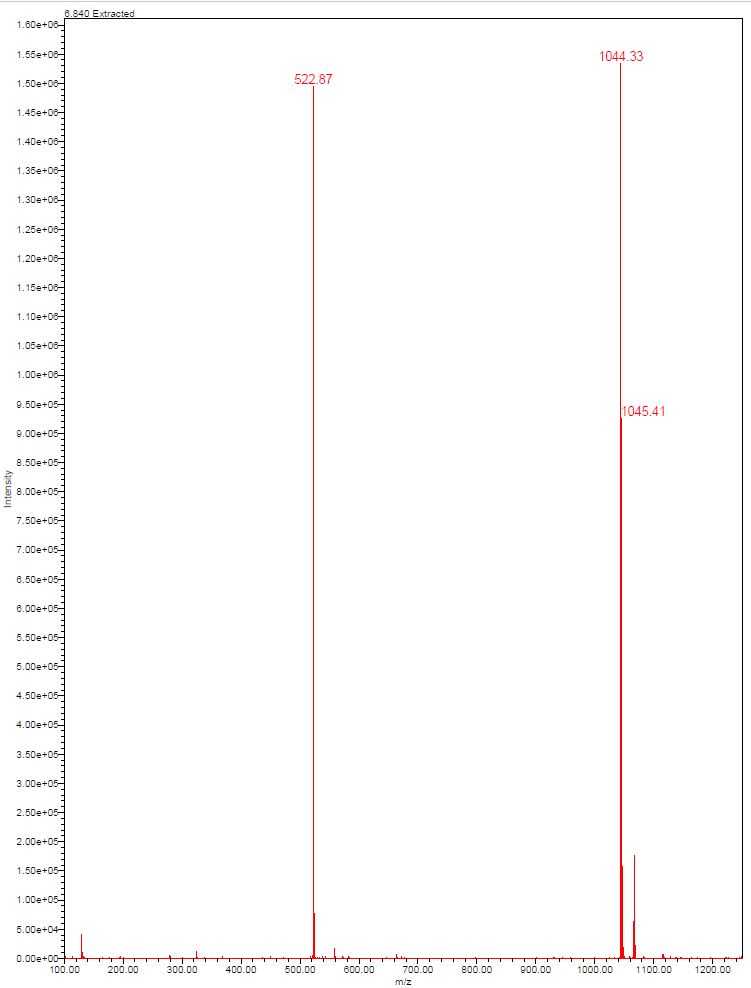


Figure S10: The MS spectrum of LW02030: calculated [M+H]^+^ (m/z) 1044.5; found 1044.3.

Synthesis of LW01128

Following general procedures, LW01128 was obtained in 10% yield after HPLC purification using 24% CH_3_CN and 0.1% TFA in H_2_O (retention time = 14.3 min).


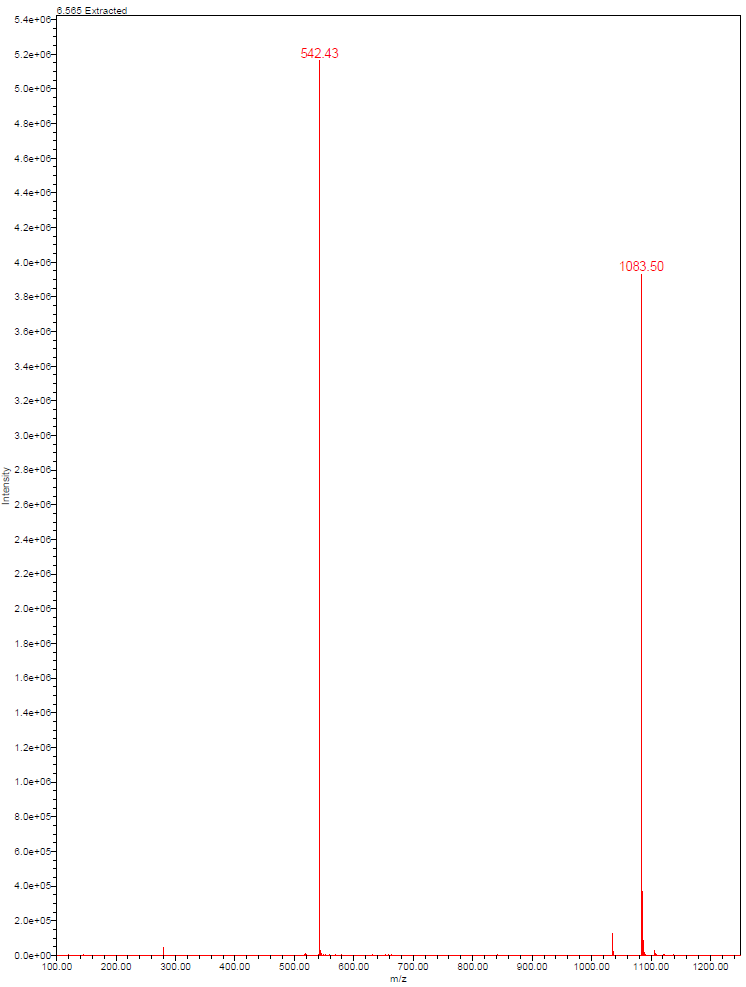


Figure S11: The MS spectrum of LW01128: calculated [M+H]^+^ (m/z) 1083.5; found 1083.5.

Synthesis of LW01136

Following general procedures, LW01136 was obtained in 30% yield after HPLC purification using 27% CH_3_CN and 0.1% TFA in H_2_O (retention time = 9.8 min).


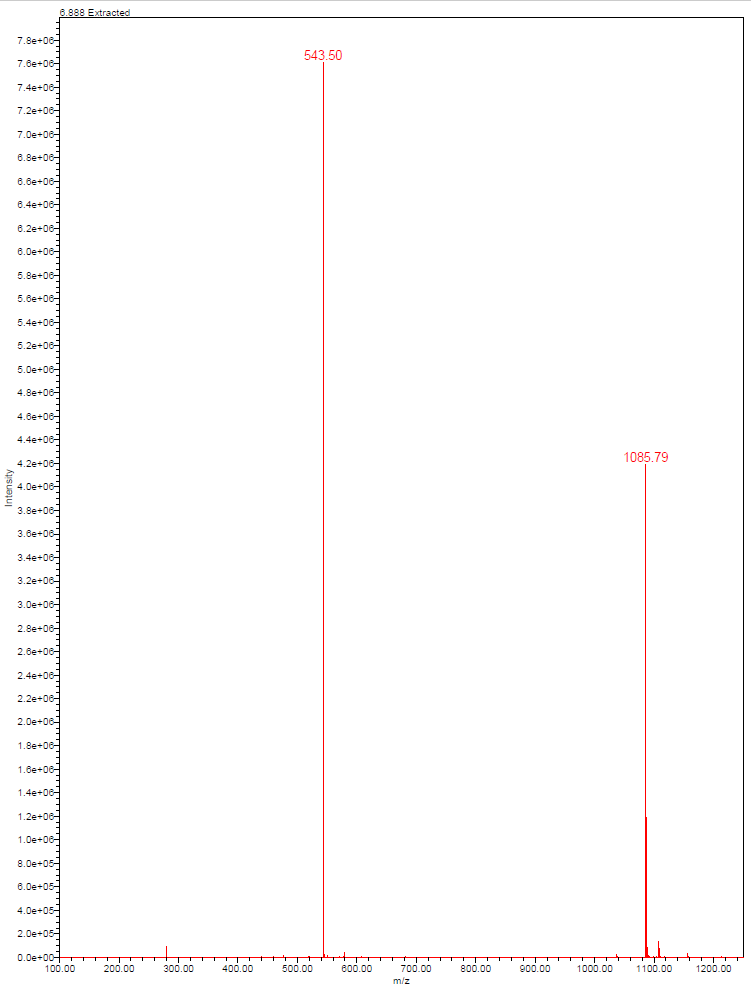


Figure S12: The MS spectrum of LW01136: calculated [M+H]^+^ (m/z) 1085.5; found 1085.8.

Synthesis of LW01137

Following general procedures, LW01137 was obtained in 18% yield after HPLC purification using 28% CH_3_CN and 0.1% TFA in H_2_O (retention time = 12.6 min).


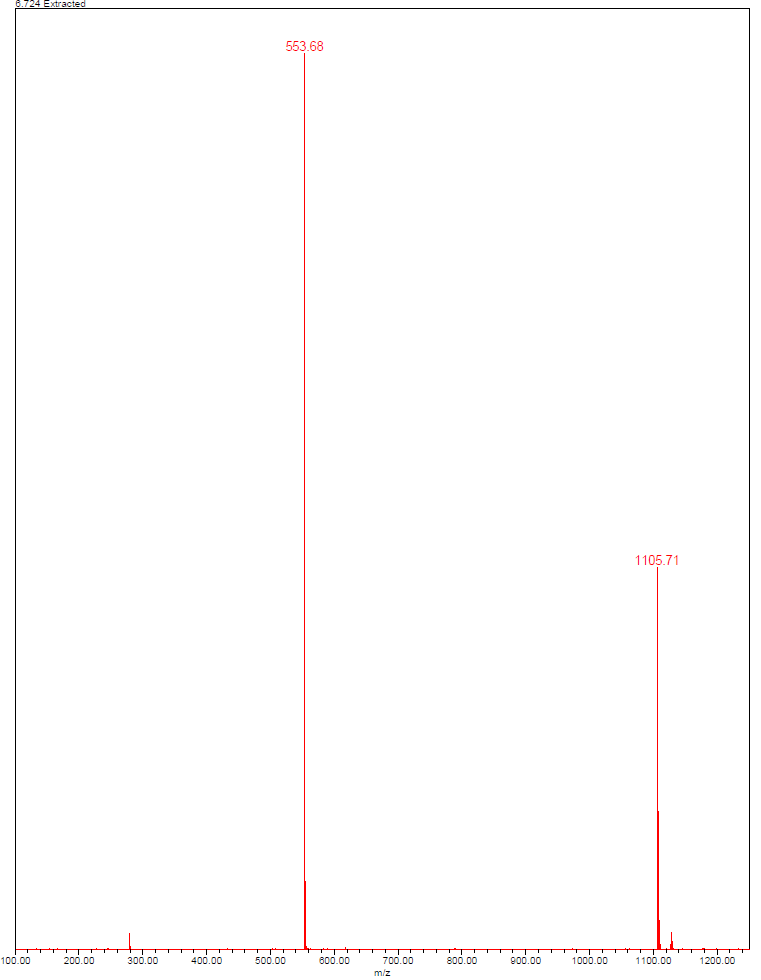


Figure S13: The MS spectrum of LW01137: calculated [M+H]^+^ (m/z) 1105.5; found 1105.7.

Synthesis of LW01183

Following general procedures, LW01183 was obtained in 26% yield after HPLC purification using 26% CH_3_CN and 0.1% TFA in H_2_O (retention time = 10.3 min).


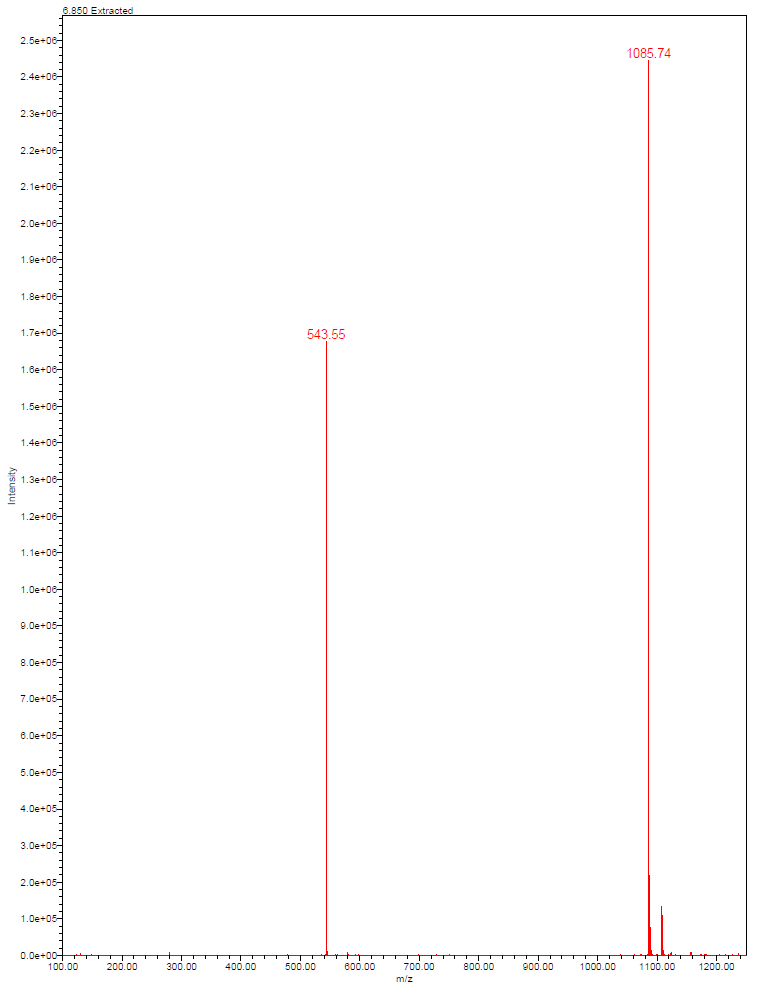


Figure S14: The MS spectrum of LW01183: calculated [M+H]^+^ (m/z) 1085.5; found 1085.7.

Synthesis of LW02009

Following general procedures, LW02009 was obtained in 23% yield after HPLC purification using 26% CH_3_CN and 0.1% TFA in H_2_O (retention time = 9.1 min).


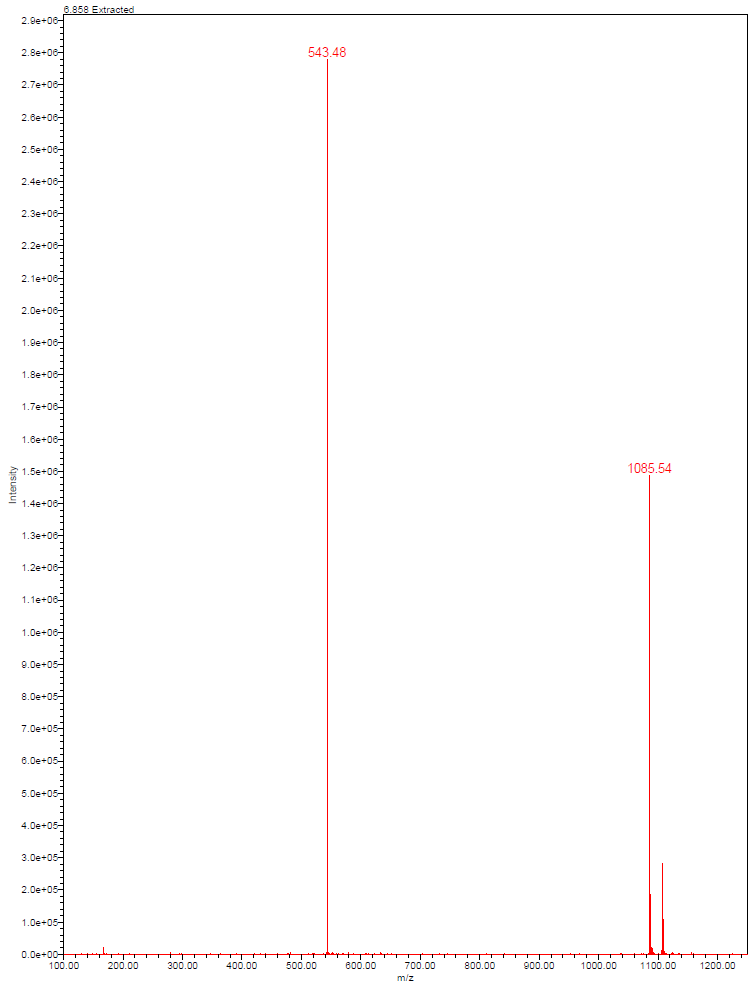


Figure S15: The MS spectrum of LW02009: calculated [M+H]^+^ (m/z) 1085.5; found 1085.5.

Synthesis of LW01177

Following general procedures, LW01177 was obtained in 24% yield after HPLC purification using 25% CH_3_CN and 0.1% TFA in H_2_O (retention time = 12.2 min).


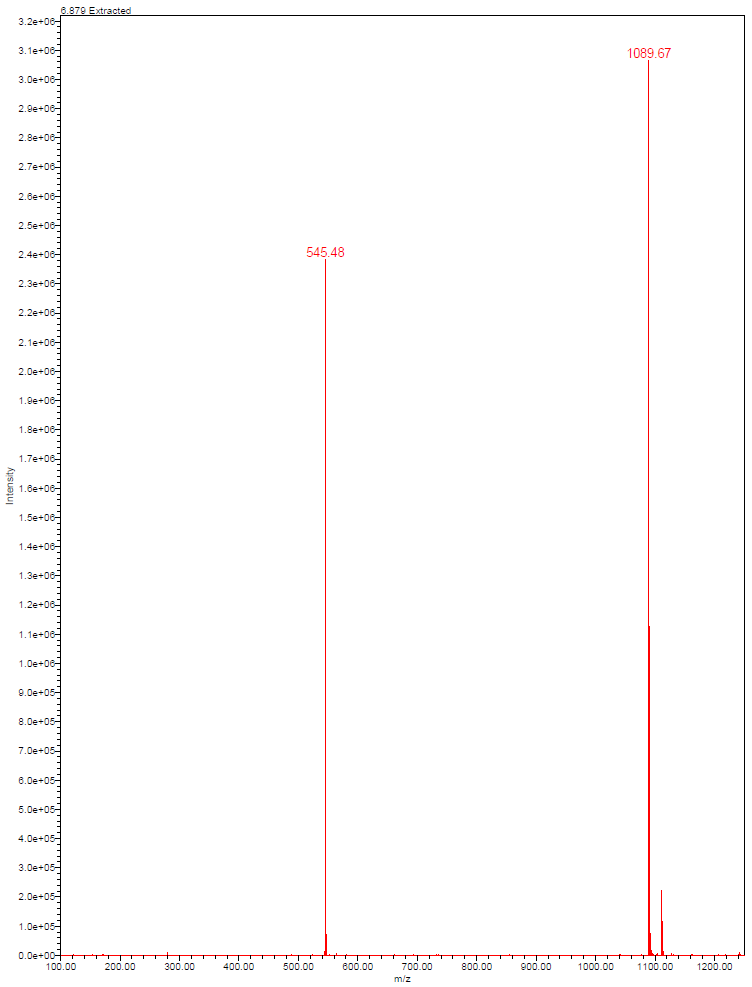


Figure S16: The MS spectrum of LW01177: calculated [M+H]^+^ (m/z) 1089.5; found 1089.7.

Synthesis of LW01191

Following general procedures, LW01191 was obtained in 20% yield after HPLC purification using 27% CH_3_CN and 0.1% TFA in H_2_O (retention time = 11.3 min).


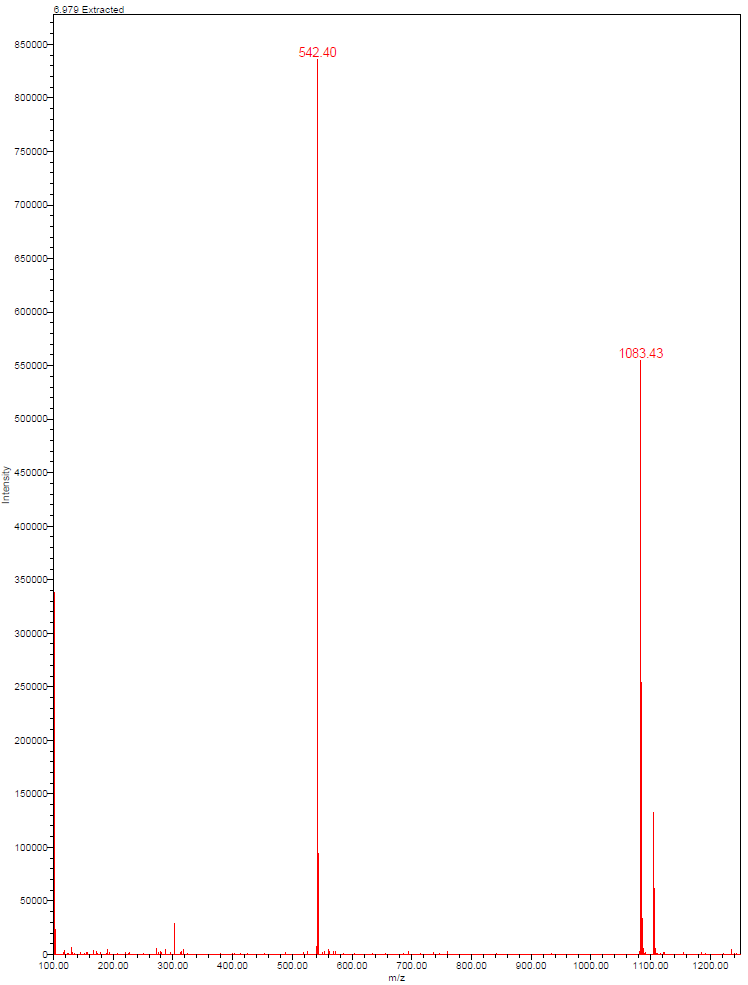


Figure S17: The MS spectrum of LW01191: calculated [M+H]^+^ (m/z) 1083.5; found 1083.4.

Synthesis of LW01173

Following general procedures, LW01173 was obtained in 21% yield after HPLC purification using 22% CH_3_CN and 0.1% TFA in H_2_O (retention time = 8.6 min).


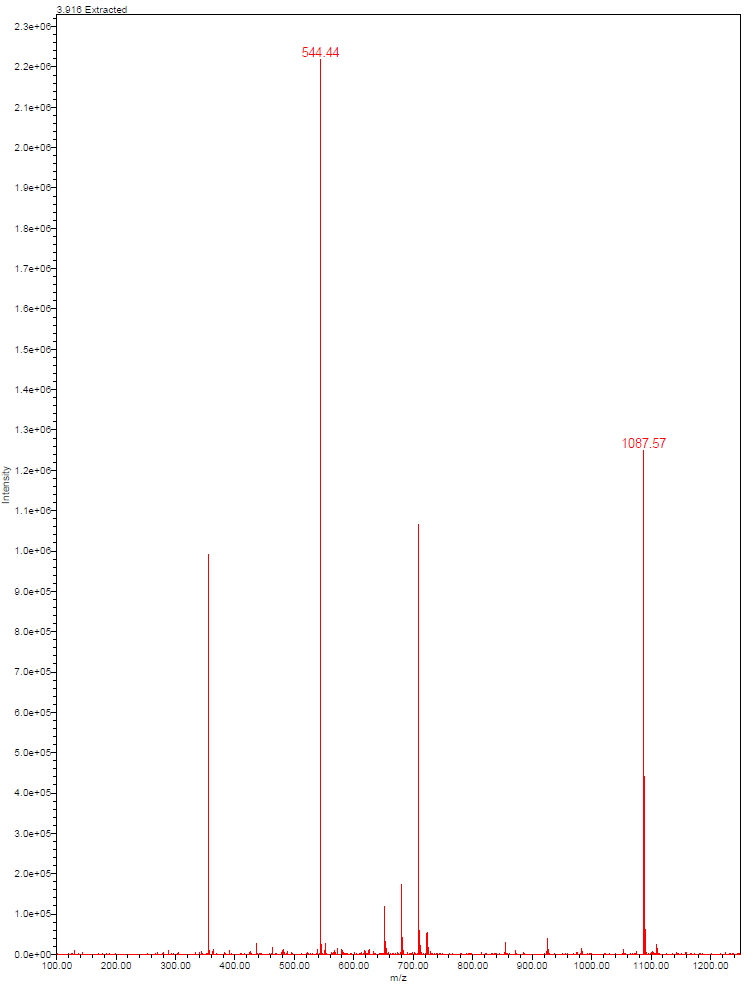


Figure S18: The MS spectrum of LW01173: calculated [M+H]^+^ (m/z) 1087.5; found 1087.6.

Synthesis of LW02007

Following general procedures, LW02007 was obtained in 25% yield after HPLC purification using 27% CH_3_CN and 0.1% TFA in H_2_O (retention time = 9.0 min).


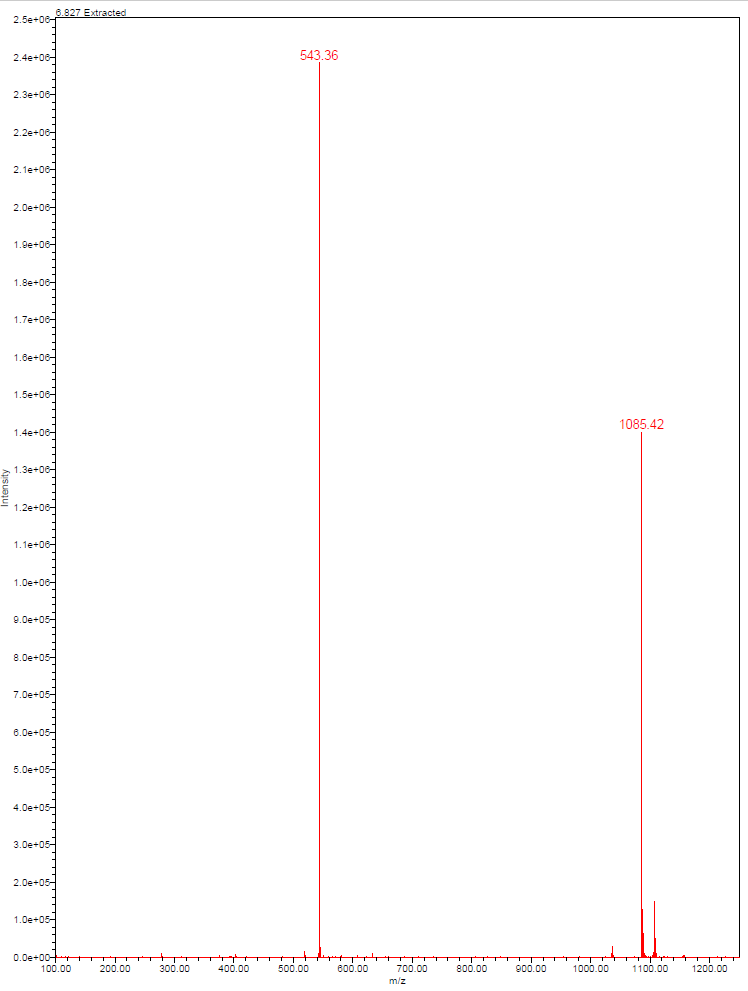


Figure S19: The MS spectrum of LW02007: calculated [M+H]^+^ (m/z) 1085.5; found 1085.4.

Synthesis of LW01171

Following general procedures, LW01171 was obtained in 29% yield after HPLC purification using 26% CH_3_CN and 0.1% TFA in H_2_O (retention time = 11.5 min).


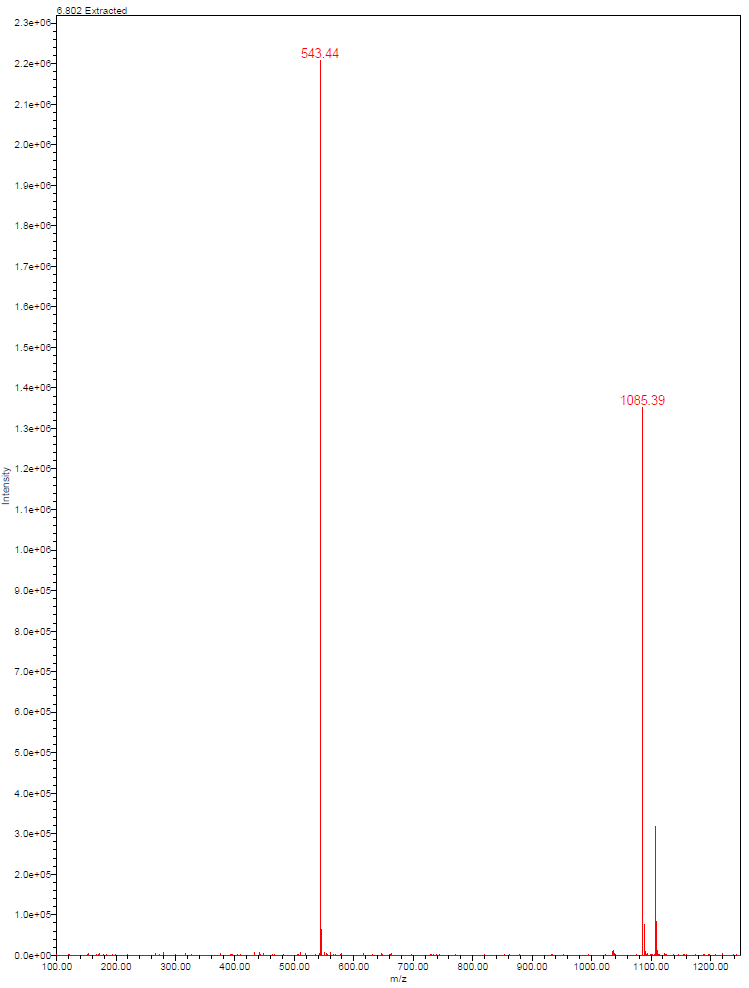


Figure S20: The MS spectrum of LW01171: calculated [M+H]^+^ (m/z) 1085.5; found 1085.4.

Synthesis of LW01182

Following general procedures, LW01182 was obtained in 33% yield after HPLC purification using 26% CH_3_CN and 0.1% TFA in H_2_O (retention time = 11.7 min).


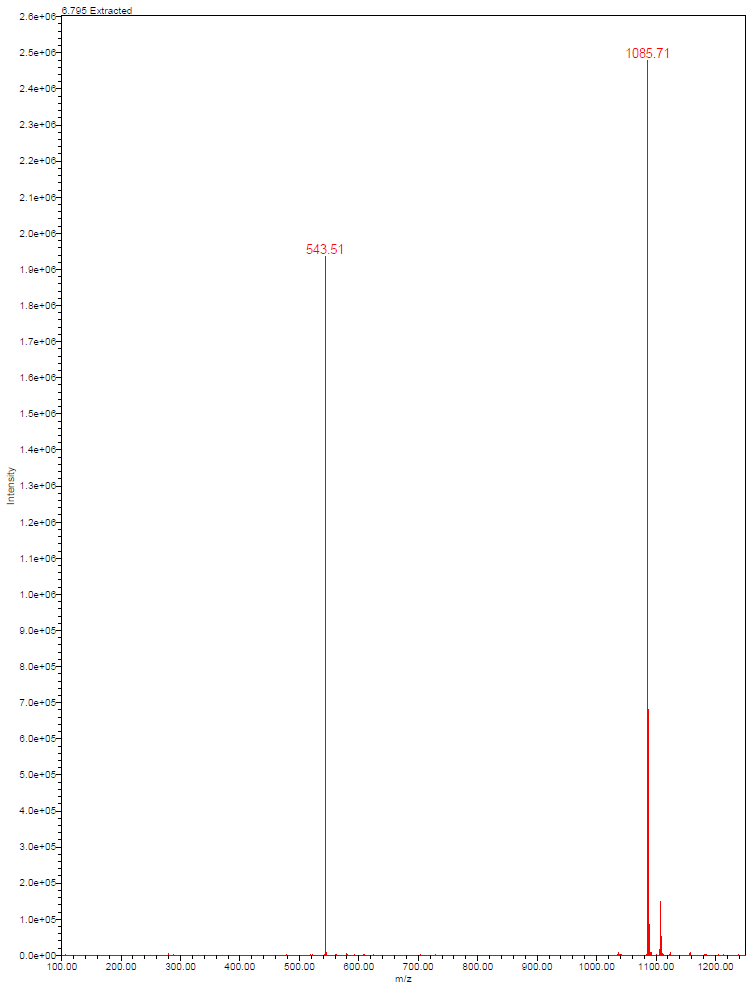


Figure S21: The MS spectrum of LW01182: calculated [M+H]^+^ (m/z) 1085.5; found 1085.7.

Synthesis of LW01175

Following general procedures, LW01175 was obtained in 40% yield after HPLC purification using 25% CH_3_CN and 0.1% TFA in H_2_O (retention time = 13.0 min).


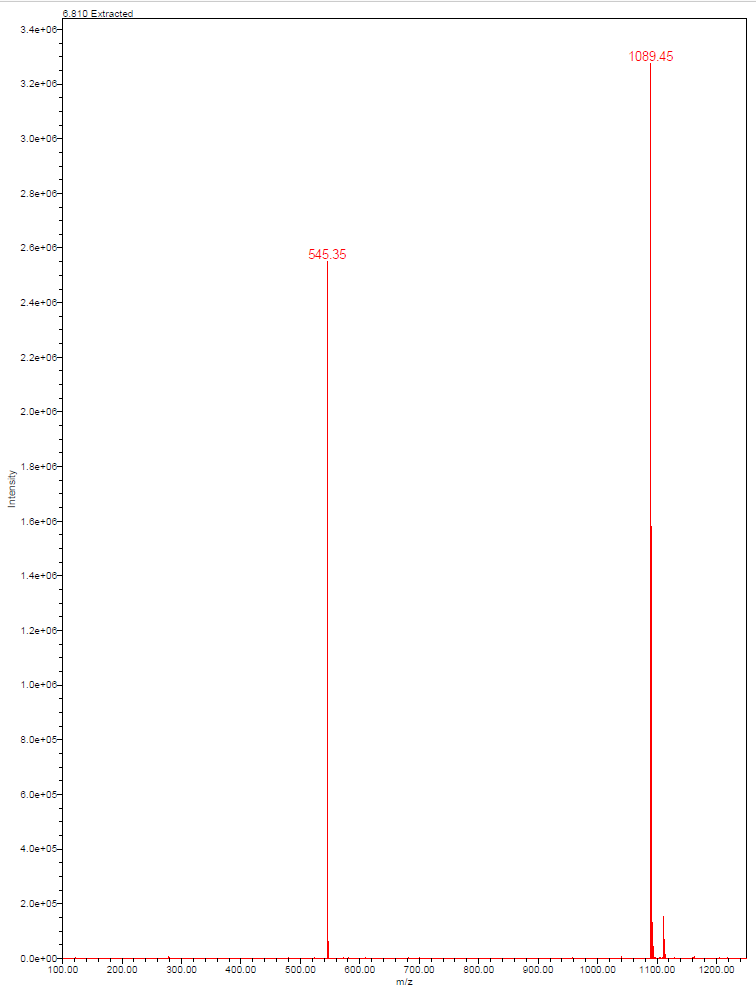


Figure S22: The MS spectrum of LW01175: calculated [M+H]^+^ (m/z) 1089.5; found 1089.5.

Synthesis of LW01166

Following general procedures, LW01166 was obtained in 46% yield after HPLC purification using 25% CH_3_CN and 0.1% TFA in H_2_O (retention time = 10.5 min)


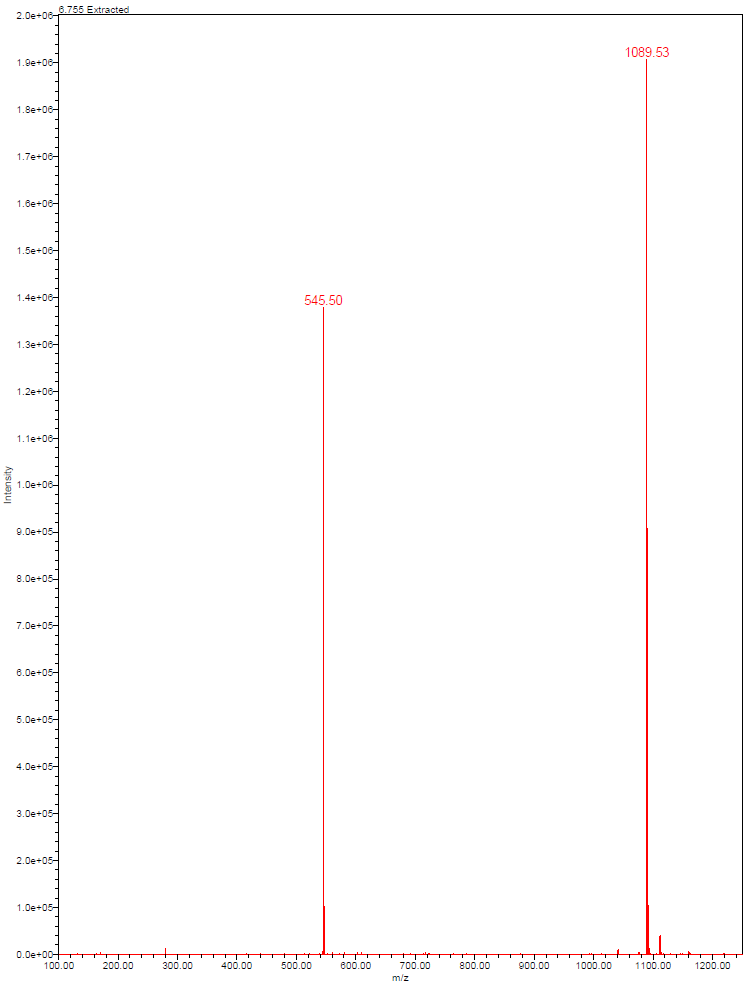


Figure S23: The MS spectrum of LW01166: calculated [M+H]^+^ (m/z) 1089.5; found 1089.5.

Synthesis of LW01180

Following general procedures, LW01180 was obtained in 36% yield after HPLC purification using 25% CH_3_CN and 0.1% TFA in H_2_O (retention time = 11.4 min).


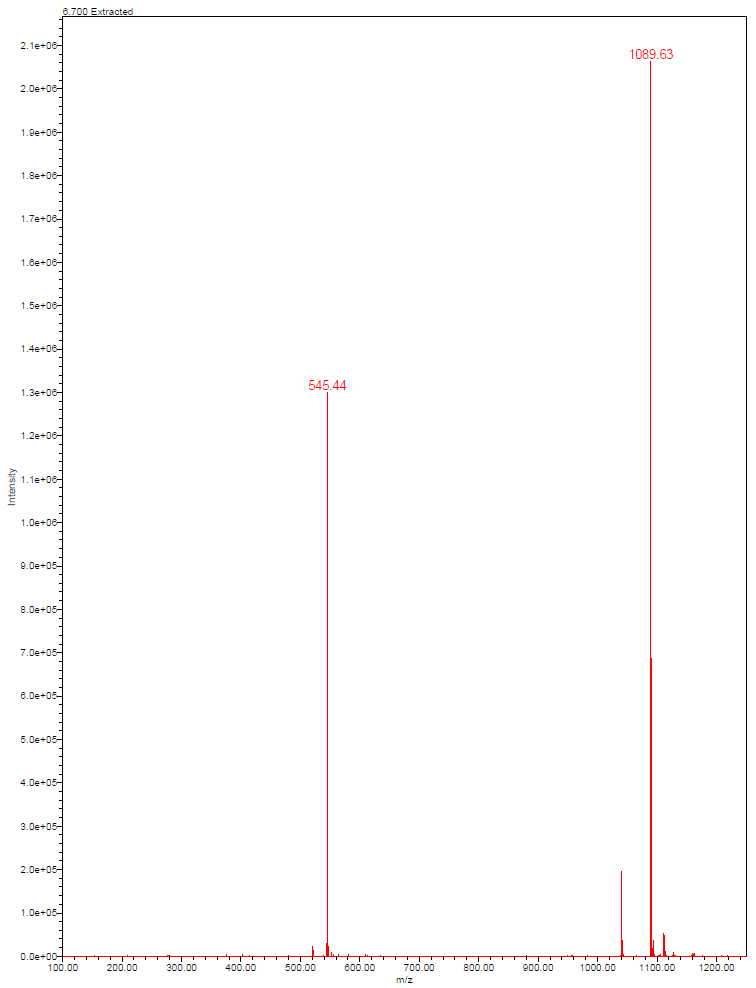


Figure S24: The MS spectrum of LW01180: calculated [M+H]^+^ (m/z) 1089.5; found 1089.6.

Synthesis of LW02013

Following general procedures, LW02013 was obtained in 8.4% yield after HPLC purification using 18% CH_3_CN and 0.1% TFA in H_2_O (retention time = 13.5 min).


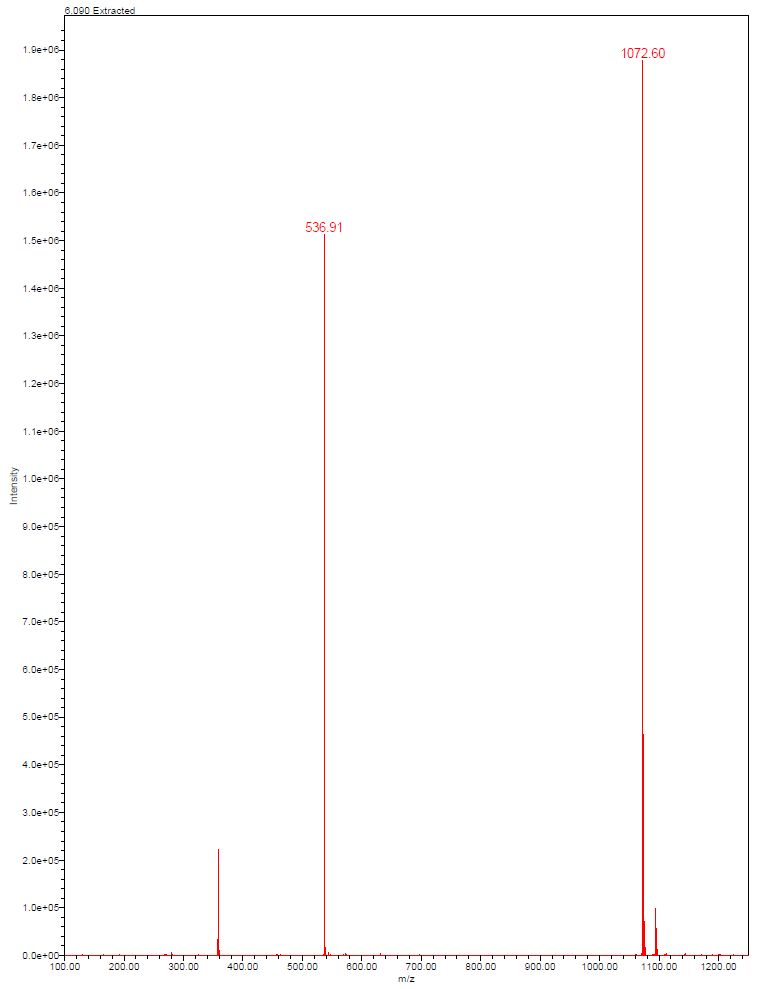


Figure S25: The MS spectrum of LW02013: calculated [M+H]^+^ (m/z) 1072.5; found 1072.6.

Synthesis of LW02015

Following general procedures, LW02015 was obtained in 24% yield after HPLC purification using 26% CH_3_CN and 0.1% TFA in H_2_O (retention time = 14.4 min).


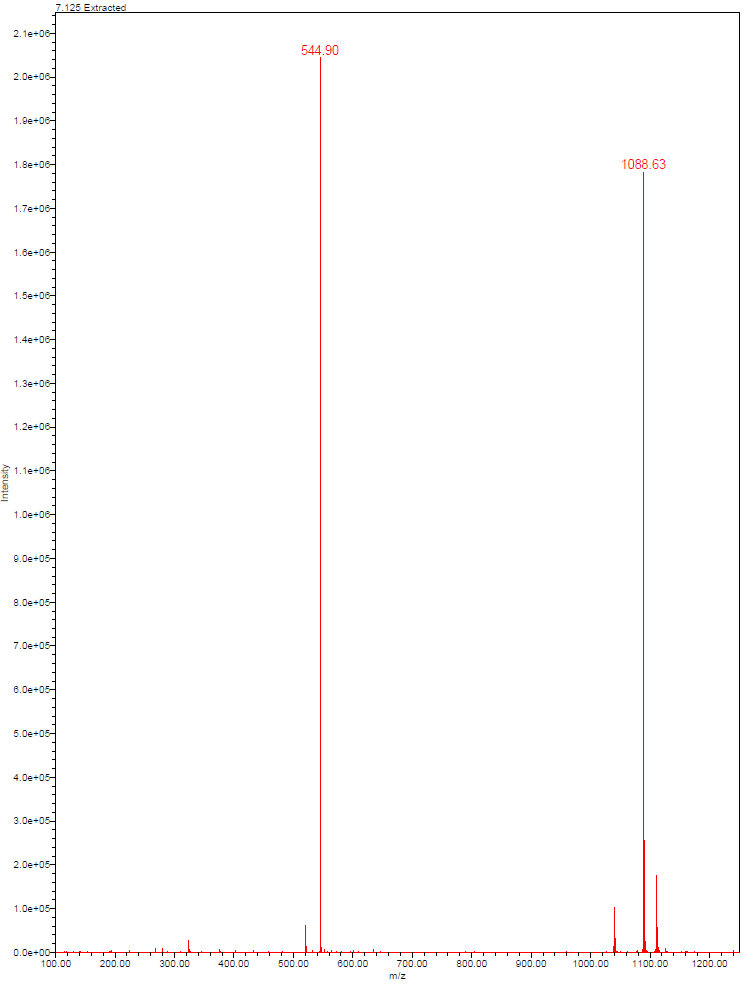


Figure S26: The MS spectrum of LW02015: calculated [M+H]^+^ (m/z) 1088.5; found 1088.6.

Synthesis of LW01107

Following general procedures, LW01107 was obtained in 19% yield after HPLC purification using 23% CH_3_CN and 0.1% TFA in H_2_O (retention time = 14.2 min).


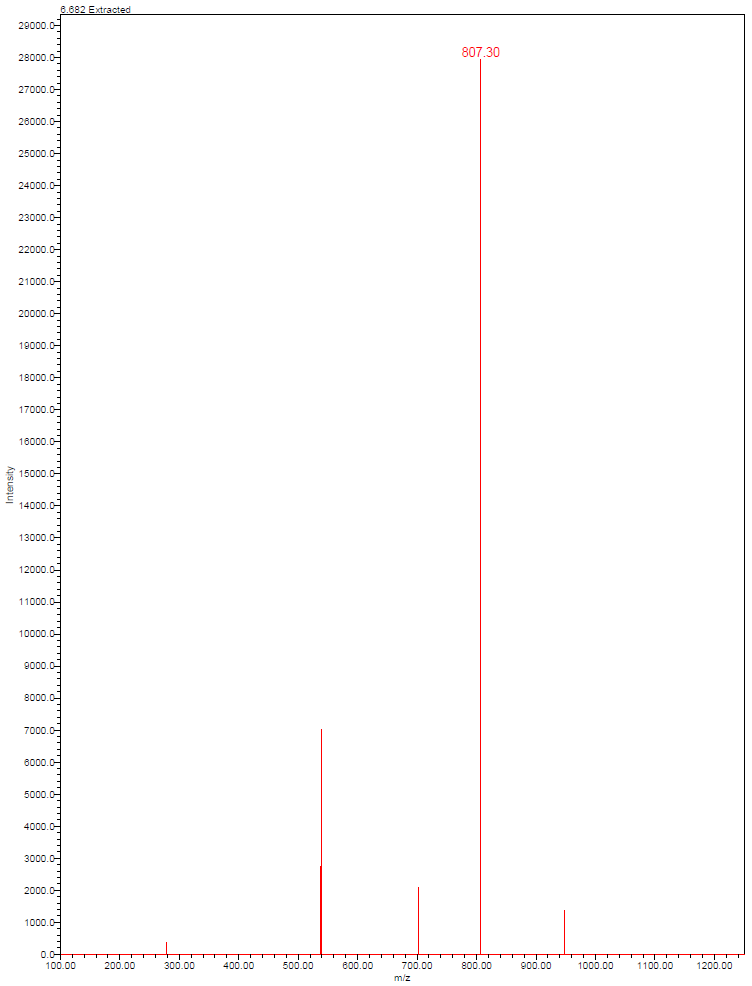


Figure S27: The MS spectrum of LW01107: calculated [M+2H]^2+^ (m/z) 806.4; found 807.3.

Synthesis of LW01108

Following general procedures, LW01108 was obtained in 26% yield after HPLC purification using 24% CH_3_CN and 0.1% TFA in H_2_O (retention time = 10.9 min).


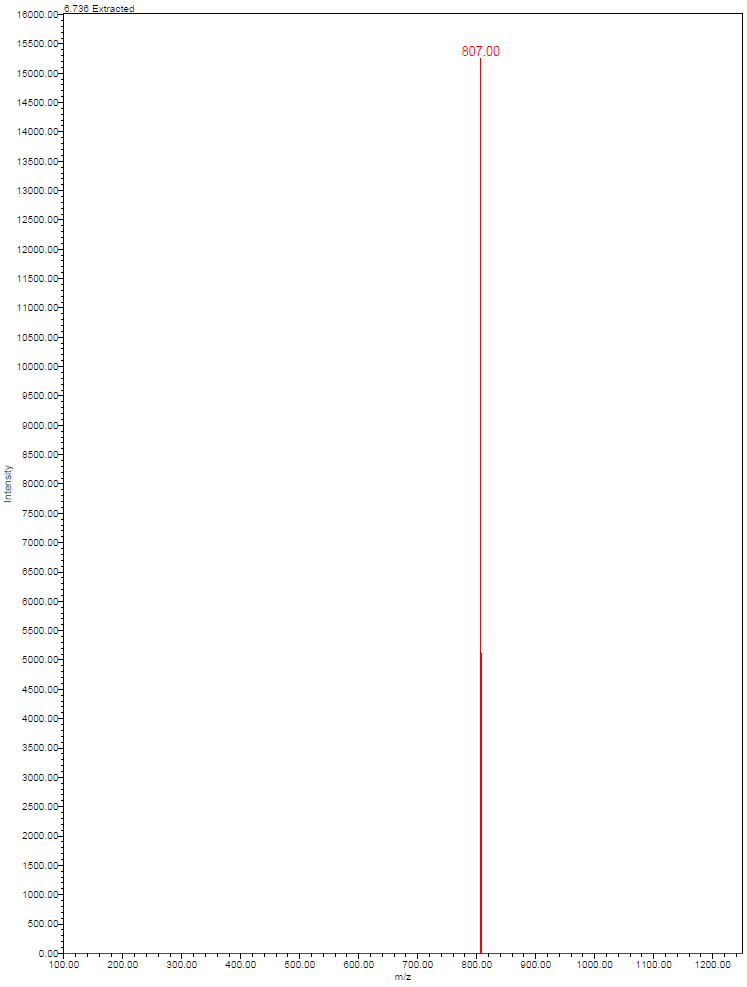


Figure S28: The MS spectrum of LW01108: calculated [M+2H]^2+^ (m/z) 806.4; found 807.0.

Synthesis of LW01149

Following general procedures, LW01149 was obtained in 4.0% yield after HPLC purification using 25% CH_3_CN and 0.1% TFA in H_2_O (retention time = 8.2 min).


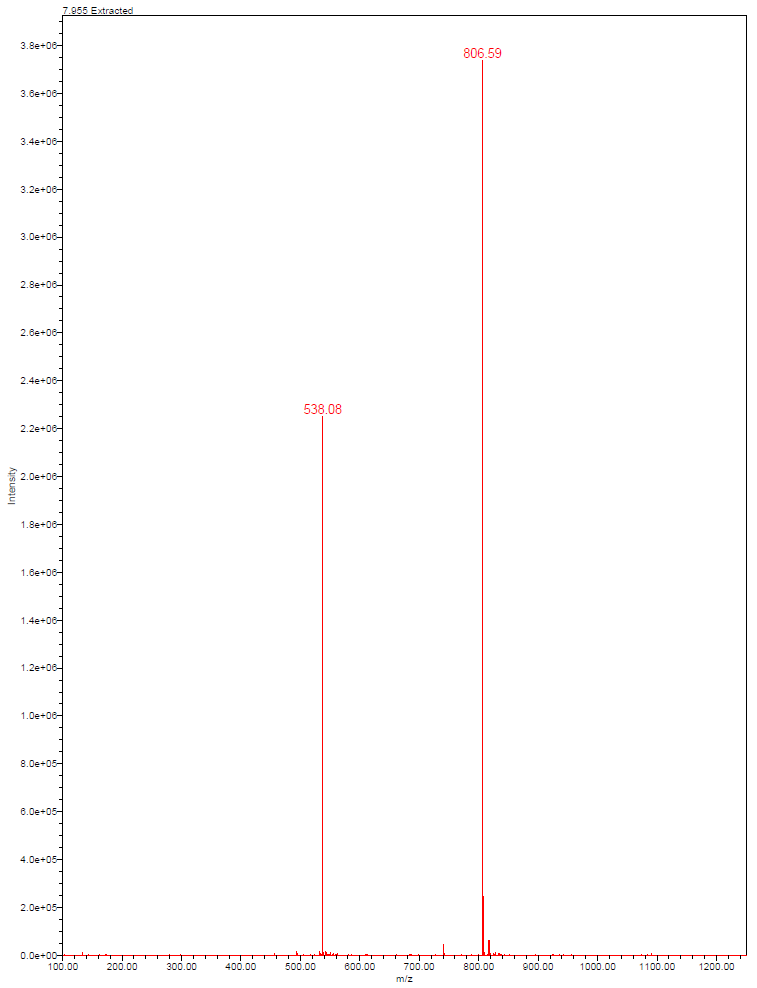


Figure S29: The MS spectrum of LW01149: calculated [M+2H]^2+^ (m/z) 806.4; found 806.6.

Synthesis of LW02021

Following general procedures, LW02021 was obtained in 33% yield after HPLC purification using 25% CH_3_CN and 0.1% TFA in H_2_O (retention time = 8.9 min).


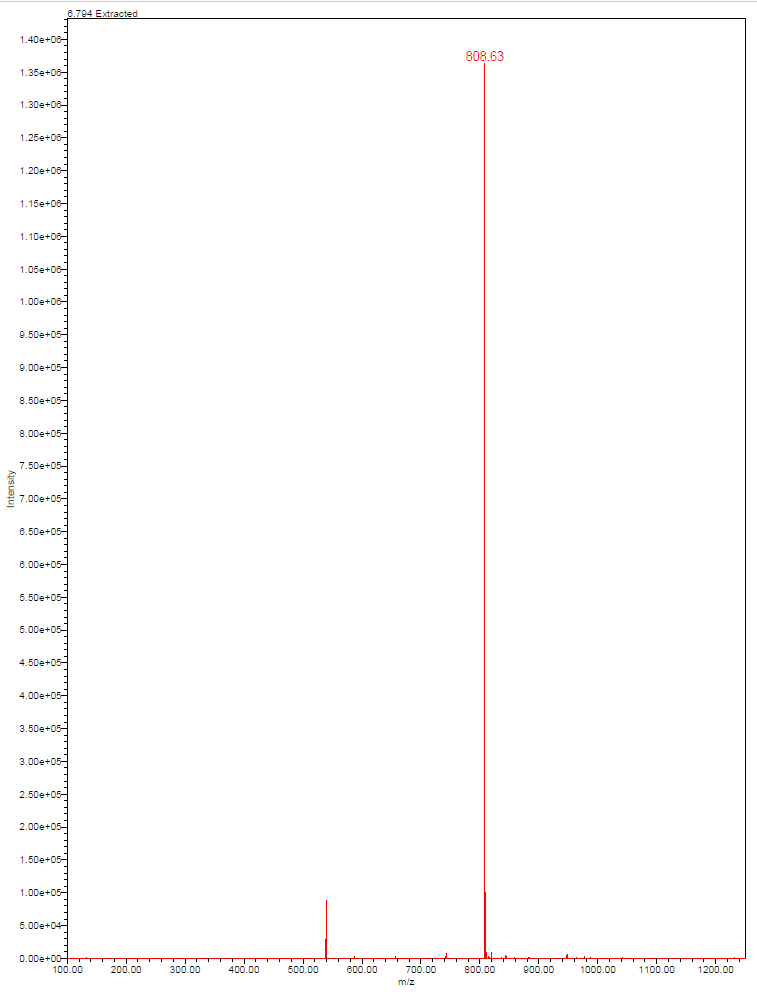


Figure S30: The MS spectrum of LW02021: calculated [M+2H]^2+^ (m/z) 808.4; found 808.6.

Synthesis of LW02023

Following general procedures, LW02023 was obtained in 19% yield after HPLC purification using 25% CH_3_CN and 0.1% TFA in H_2_O (retention time = 12.2 min).


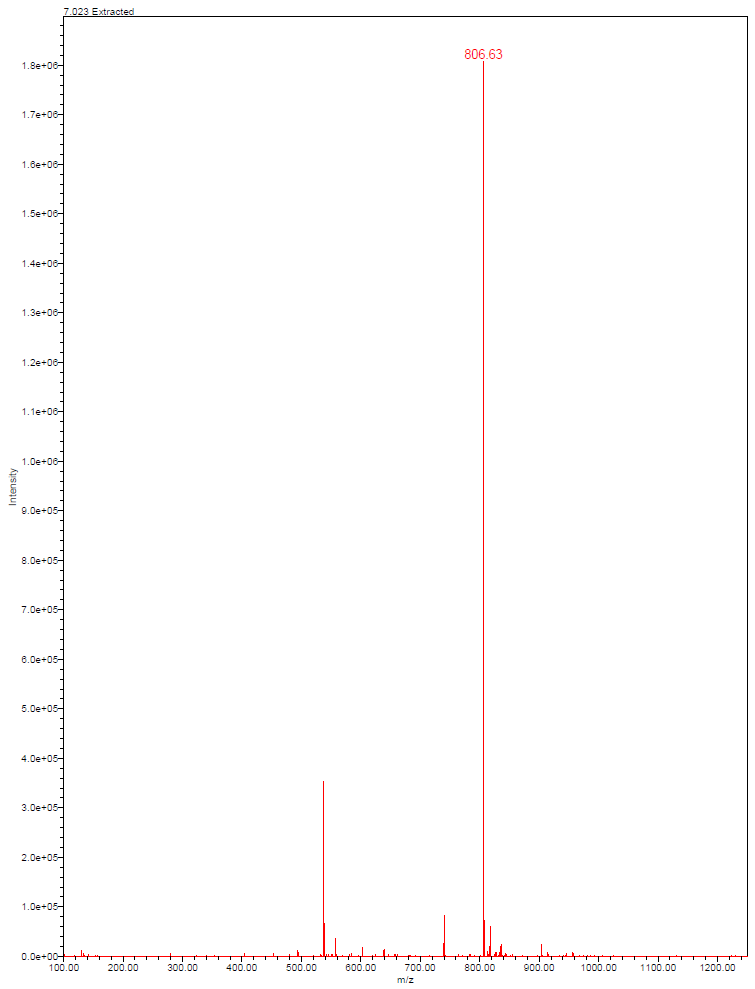


Figure S31: The MS spectrum of LW02023: calculated [M+2H]^2+^ (m/z) 806.4; found 806.6.

Synthesis of LW02025

Following general procedures, LW02025 was obtained in 16% yield after HPLC purification using 23% CH_3_CN and 0.1% TFA in H_2_O (retention time = 13.7 min).


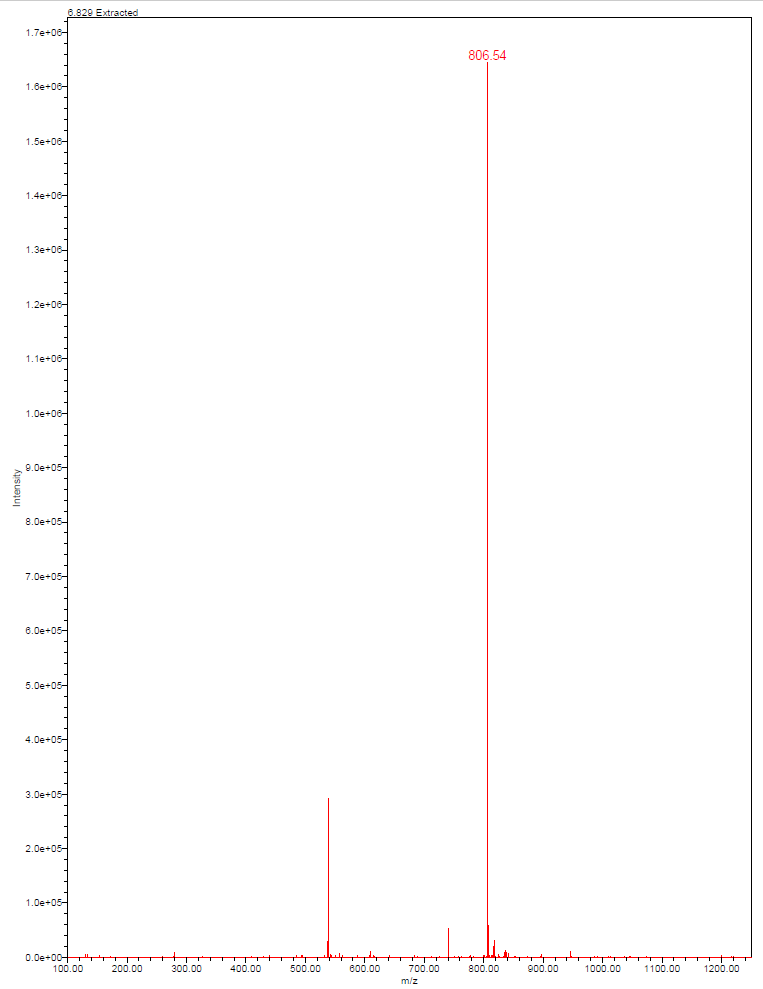


Figure S32: The MS spectrum of LW02025: calculated [M+2H]^2+^ (m/z) 806.4; found 806.5.

Synthesis of LW01110

Following general procedures, LW01110 was obtained in 11% yield after HPLC purification using 24% CH_3_CN and 0.1% TFA in H_2_O (retention time = 14.9 min).


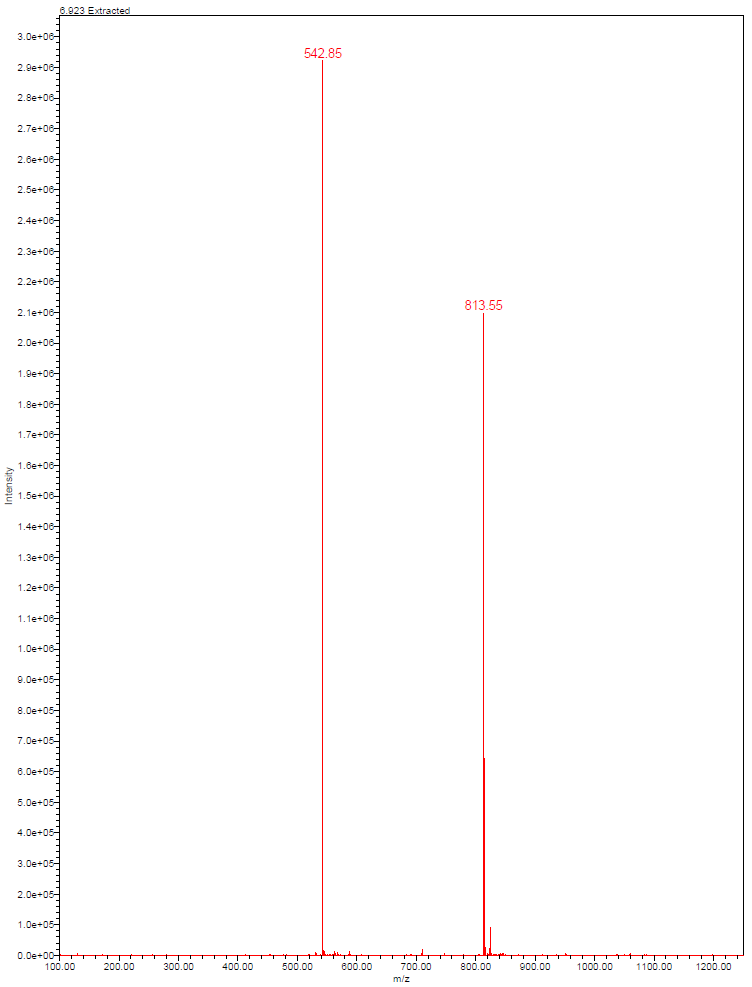


Figure S33: The MS spectrum of LW01110: calculated [M+2H]^2+^ (m/z) 813.4; found 813.6.

Synthesis of LW01142

Following general procedures, LW01142 was obtained in 17% yield after HPLC purification using 25% CH_3_CN and 0.1% TFA in H_2_O (retention time = 12.4 min).


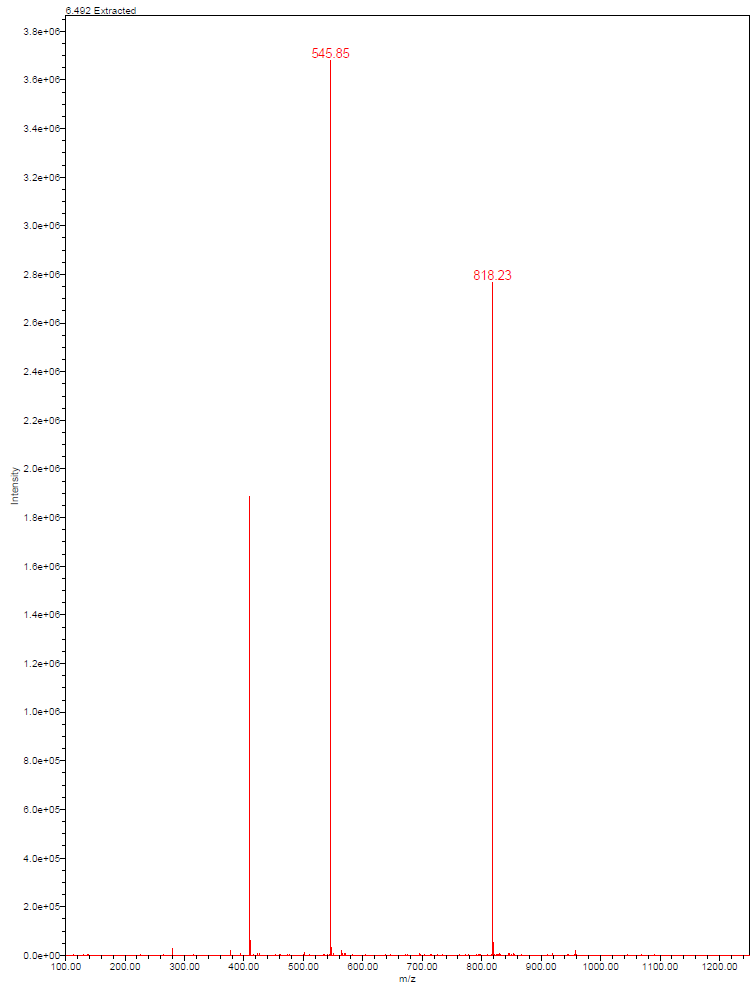


Figure S34: The MS spectrum of LW01142: calculated [M+2H]^2+^ (m/z) 817.9; found 818.2.

Synthesis of LW01143

Following general procedures, LW01143 was obtained in 16% yield after HPLC purification using 26% CH_3_CN and 0.1% TFA in H_2_O (retention time = 12.0 min).


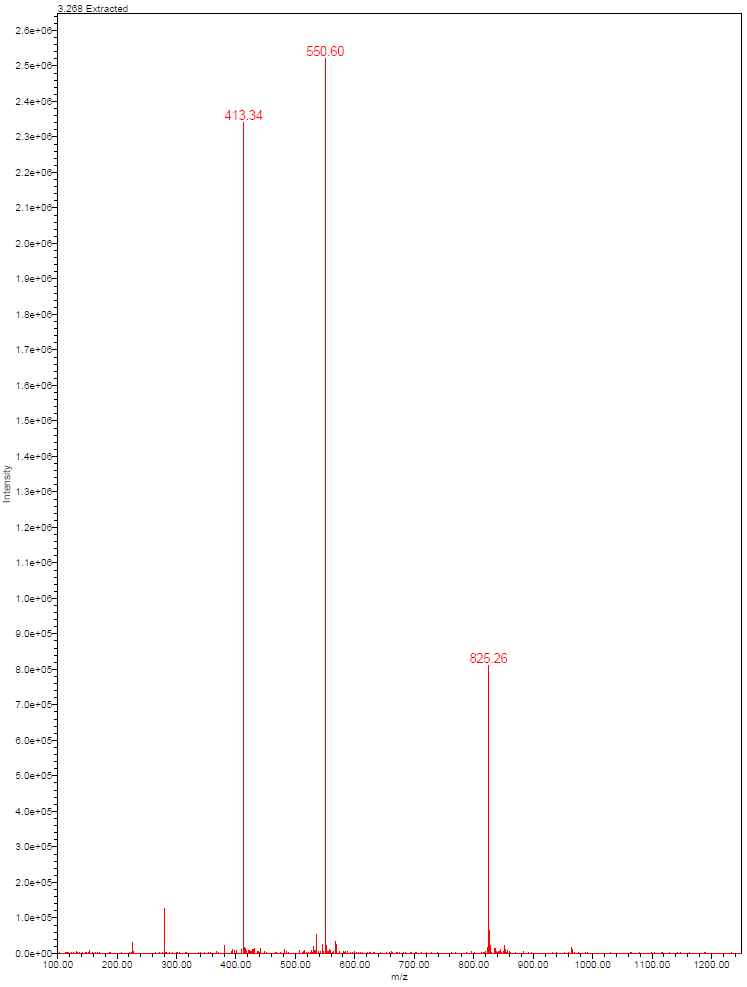


Figure S35: The MS spectrum of LW01143: calculated [M+2H]^2+^ (m/z) 824.9; found 825.3.

Synthesis of LW02040

Following general procedures, LW02040 was obtained in 21% yield after HPLC purification using 25% CH_3_CN and 0.1% TFA in H_2_O (retention time = 14.7 min).


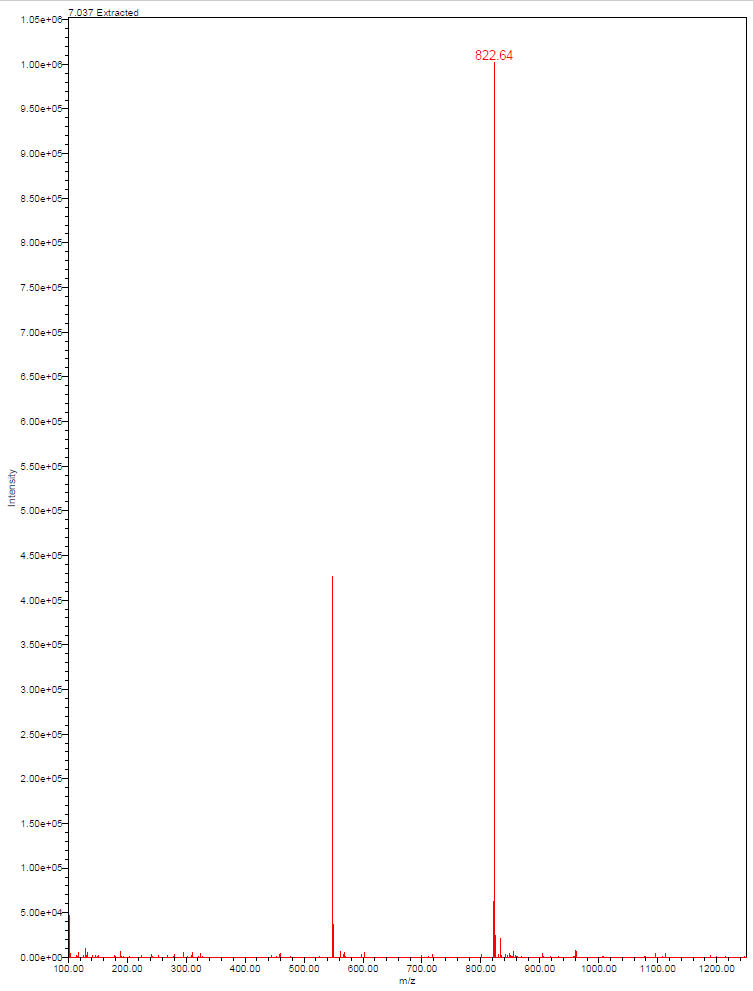


Figure S36: The MS spectrum of LW02040: calculated [M+2H]^2+^ (m/z) 822.4; found 822.6.

**General procedures for the synthesis of nonradioactive Ga-complexed standards**

The nonradioactive Ga-complexed standards were prepared by heating a solution of the DOTA-conjugated precursor with GaCl_3_ (5 eq.) in NaOAc buffer (0.1 M, 500 µL, pH 4.2 – 4.5) at 80 °C for 15 min. The reaction mixture was then purified via HPLC (semi-preparative column, flow rate: 4.5 mL/min). The HPLC eluates containing the desired peptide were collected and lyophilized. The purities of all nonradioactive Ga-complexed standards were ≥ 97% as determined by HPLC (analytical column, flow rate: 2.0 mL/min, Table S3).

Table S3: Peptide sequences and purities of Ga-complexed DOTA-conjugated GRPR-targeted peptides. The substituted unnatural amino acids are in bold.

| Name | Sequence | Purity (%) |
| --- | --- | --- |
| Ga-LW01107 | Ga-DOTA-Pip-D-Phe-Gln-Trp-Ala-Val-Gly-**NMe-His**-Leu-Thz-NH_2_ | 99 |
| Ga-LW01108 | Ga-DOTA-Pip-D-Phe-Gln-Trp-Ala-**Tle**-Gly-His-Leu-Thz-NH_2_ | 99 |
| Ga-LW01149 | Ga-DOTA-Pip-D-Phe-Gln-**α-Me-Trp**-Ala-Val-Gly-His-Leu-Thz-NH_2_ | 97 |
| Ga-LW02021 | Ga-DOTA-Pip-D-Phe-Gln-**7-F-Trp**-Ala-Val-Gly-His-Leu-Thz-NH_2_ | 99 |
| Ga-LW02023 | Ga-DOTA-Pip-D-Phe-Gln-**5-Me-Trp**-Ala-Val-Gly-His-Leu-Thz-NH_2_ | 98 |
| Ga-LW02025 | Ga-DOTA-Pip-D-Phe-Gln-**2-Me-Trp**-Ala-Val-Gly-His-Leu-Thz-NH_2_ | 99 |
| Ga-LW01110 | Ga-DOTA-Pip-D-Phe-Gln-Trp-Ala-**Tle**-Gly-**NMe-His**-Leu-Thz-NH_2_ | 98 |
| Ga-LW01142 | Ga-DOTA-Pip-D-Phe-His-Trp-Ala-**Tle**-Gly-**NMe-His**-Leu-Thz-NH_2_ | 99 |
| Ga-LW01143 | Ga-DOTA-Pip-D-Phe-His-Trp-Ala-**Tle**-**NMe-Gly**-**NMe-His**-Leu-Thz-NH_2_ | 99 |
| Ga-LW02040 | Ga-DOTA-Pip-D-Phe-Gln-**7-F-Trp**-Ala-**Tle**-Gly-**NMe-His**-Leu-Thz-NH_2_ | 99 |

Synthesis of Ga-LW01107

Following general procedures, Ga-LW01107 was obtained in 73% yield after HPLC purification using 23% CH_3_CN and 0.1% TFA in H_2_O (retention time = 14.2 min).


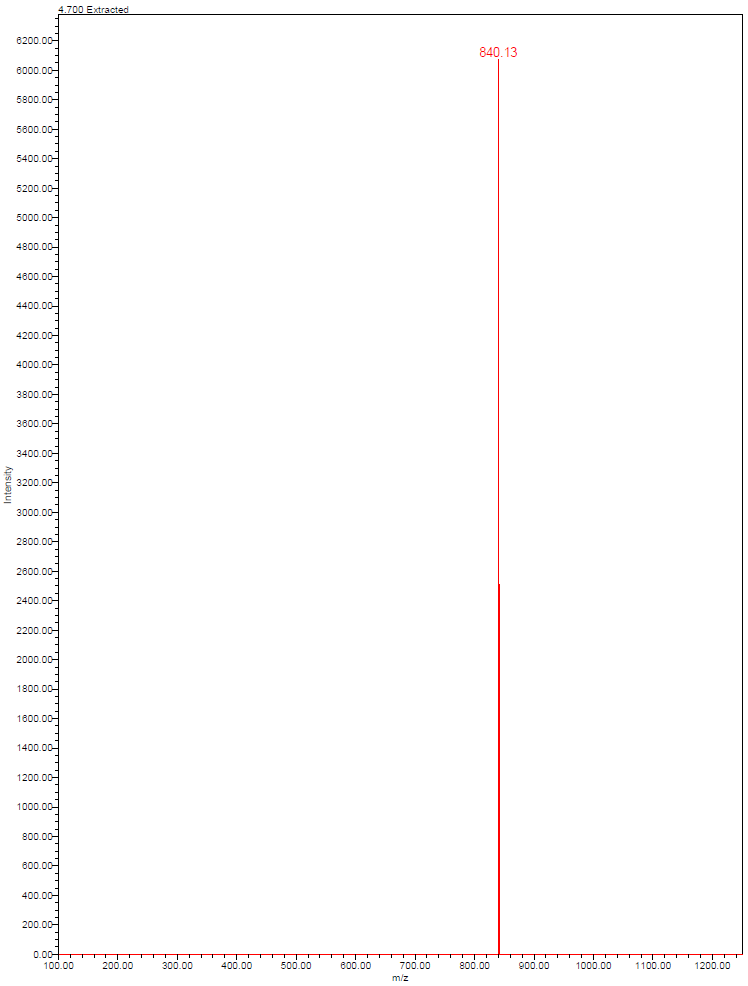


Figure S37: The MS spectrum of Ga-LW01107: calculated [M+2H]^2+^ (m/z) 839.9; found 840.1.

Synthesis of Ga-LW01108

Following general procedures, Ga-LW01108 was obtained in 59% yield after HPLC purification using 24% CH_3_CN and 0.1% TFA in H_2_O (retention time = 13.2 min).


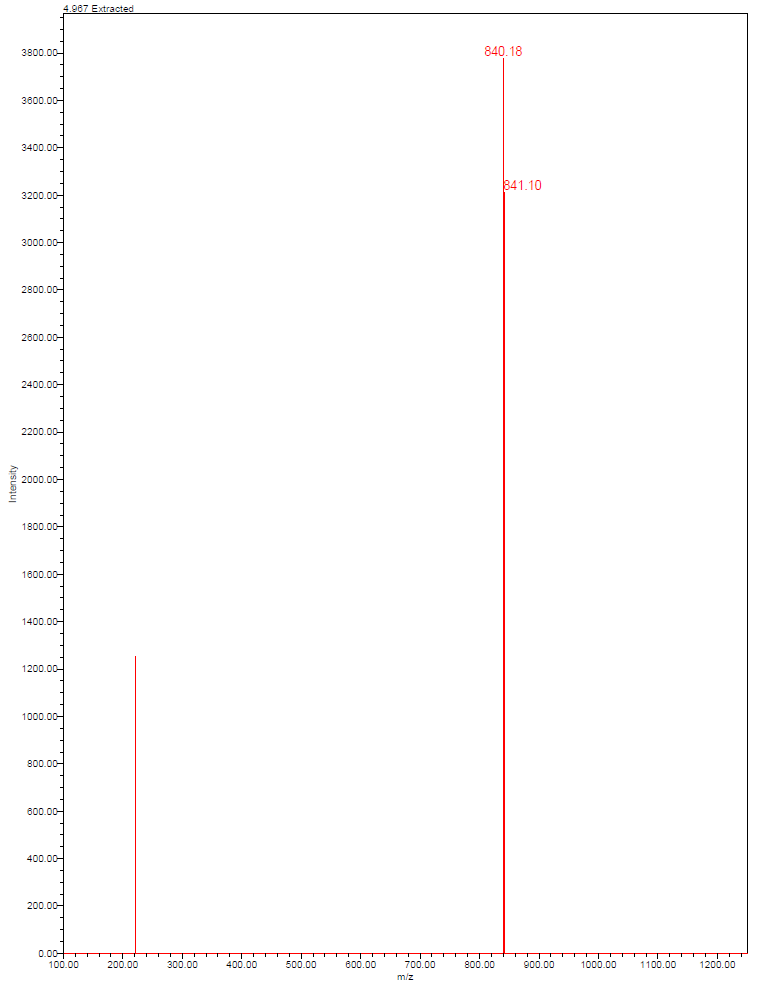


Figure S38: The MS spectrum of Ga-LW01108: calculated [M+2H]^2+^ (m/z) 839.9; found 840.2.

Synthesis of Ga-LW01149

Following general procedures, Ga-LW01149 was obtained in 72% yield after HPLC purification using 24% CH_3_CN and 0.1% TFA in H_2_O (retention time = 13.2 min).


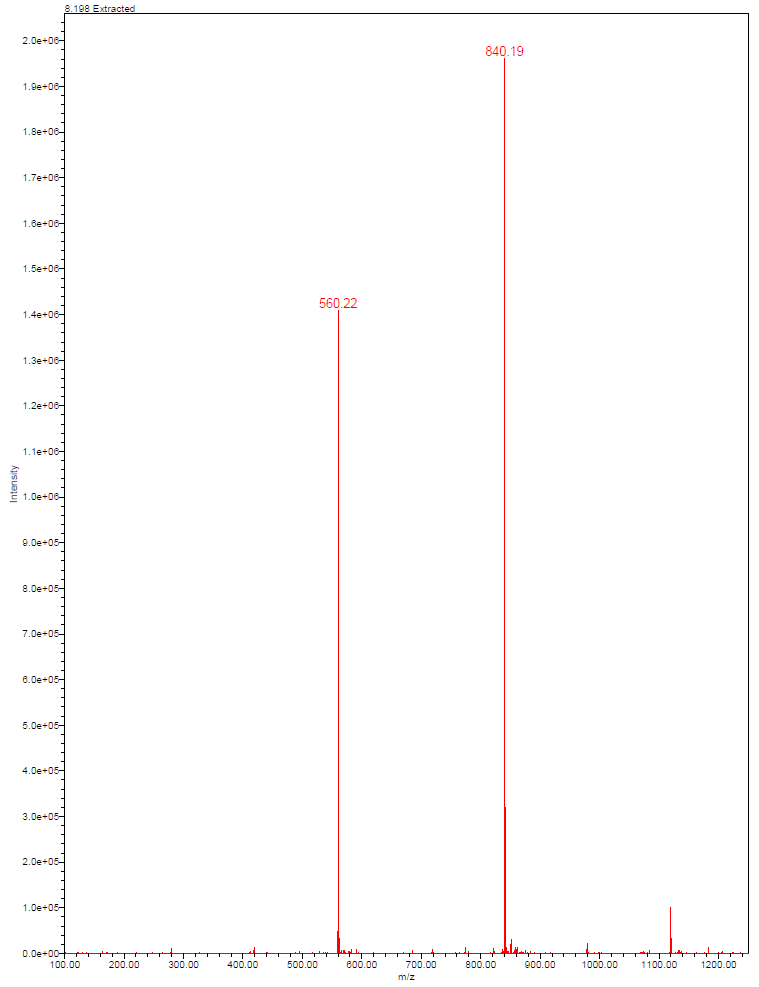


Figure S39: The MS spectrum of Ga-LW01149: calculated [M+2H]^2+^ (m/z) 839.2; found 840.2.

Synthesis of Ga-LW02021

Following general procedures, Ga-LW02021 was obtained in 82% yield after HPLC purification using 25% CH_3_CN and 0.1% TFA in H_2_O (retention time = 14.4 min).


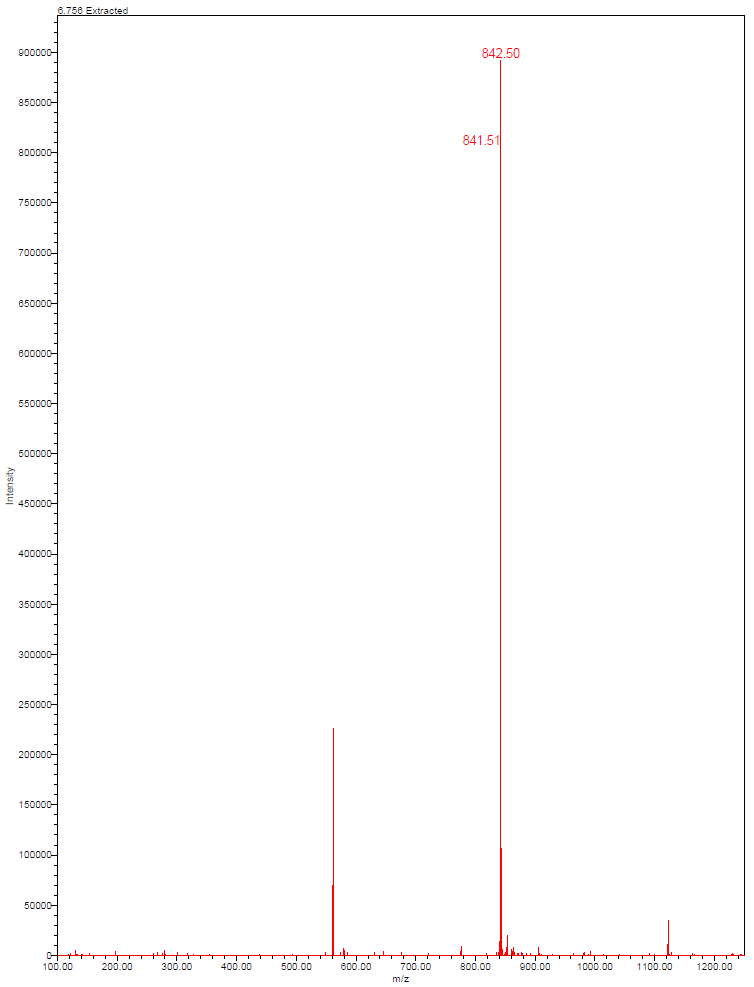


Figure S40: The MS spectrum of Ga-LW02021: calculated [M+2H]^2+^ (m/z) 841.9; found 842.5.

Synthesis of Ga-LW02023

Following general procedures, Ga-LW02023 was obtained in 84% yield after HPLC purification using 25% CH_3_CN and 0.1% TFA in H_2_O (retention time = 13.1 min).


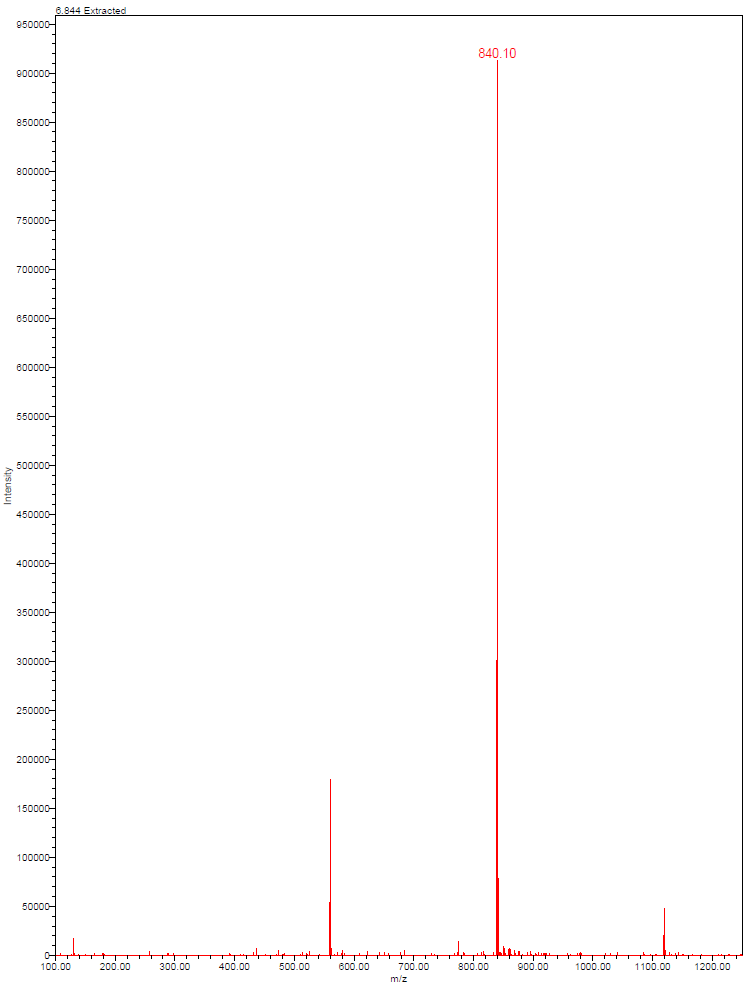


Figure S41: The MS spectrum of Ga-LW02023: calculated [M+2H]^2+^ (m/z) 839.9; found 840.1.

Synthesis of Ga-LW02025

Following general procedures, Ga-LW02025 was obtained in 81% yield after HPLC purification using 23% CH_3_CN and 0.1% TFA in H_2_O (retention time = 16.8 min).


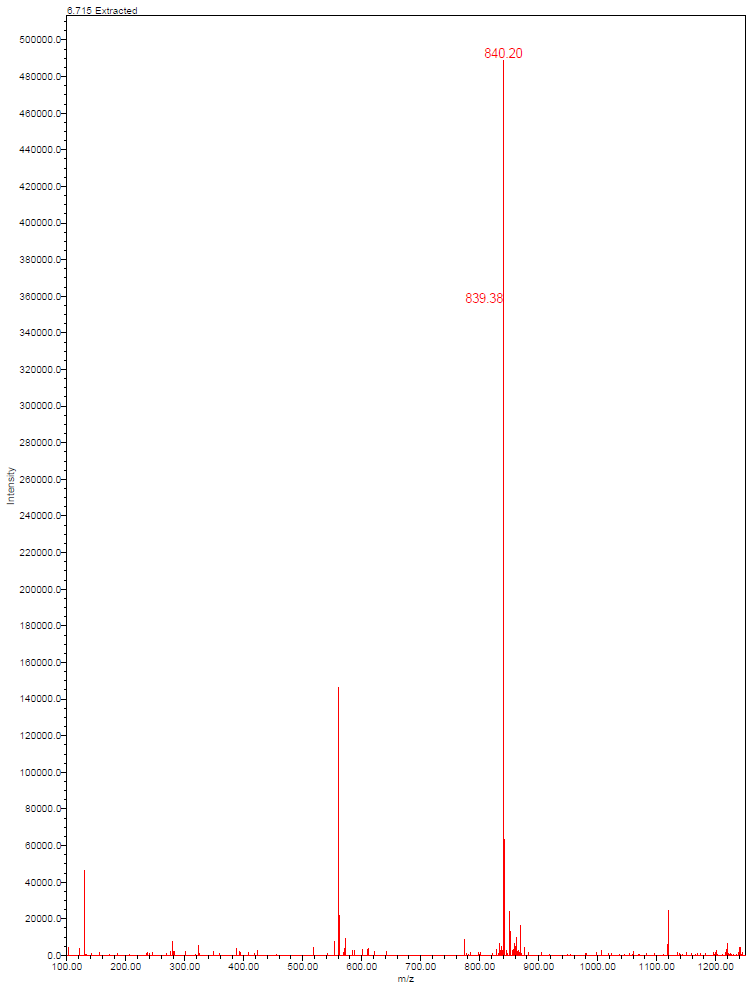


Figure S42: The MS spectrum of Ga-LW02025: calculated [M+2H]^2+^ (m/z) 839.9; found 840.2.

Synthesis of Ga-LW01110

Following general procedures, Ga-LW01110 was obtained in 54% yield after HPLC purification using 24% CH_3_CN and 0.1% TFA in H_2_O (retention time = 15.8 min).


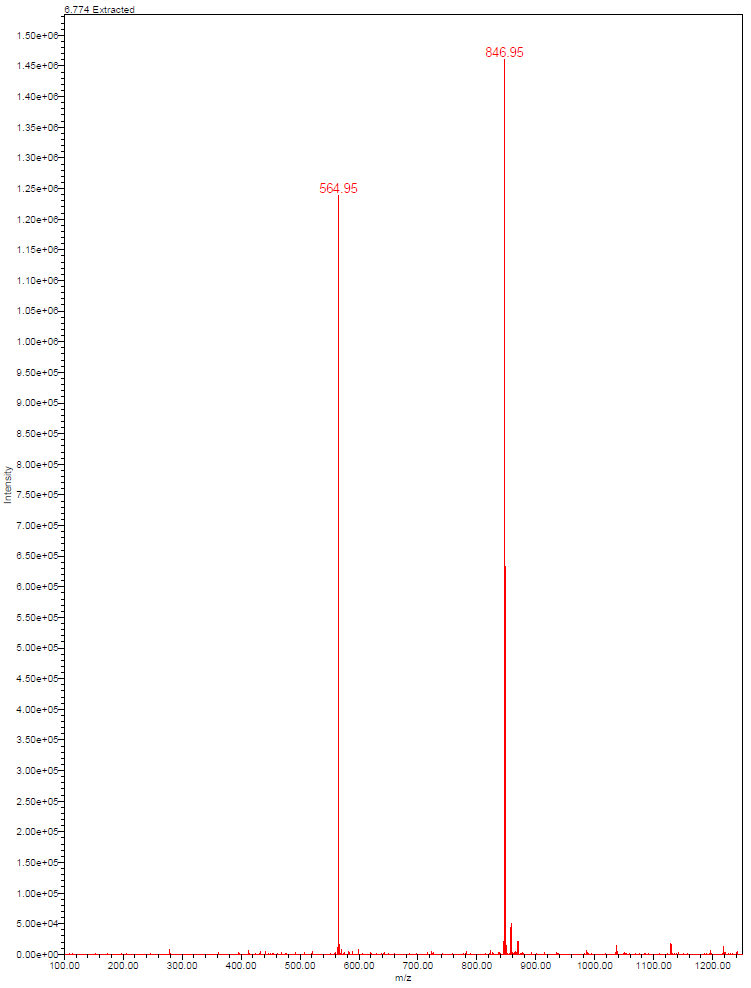


Figure S43: The MS spectrum of Ga-LW01110: calculated [M+2H]^2+^ (m/z) 846.9; found 847.0.

Synthesis of Ga-LW01142

Following general procedures, Ga-LW01142 was obtained in 53% yield after HPLC purification using 25% CH_3_CN and 0.1% TFA in H_2_O (retention time = 11.1 min).


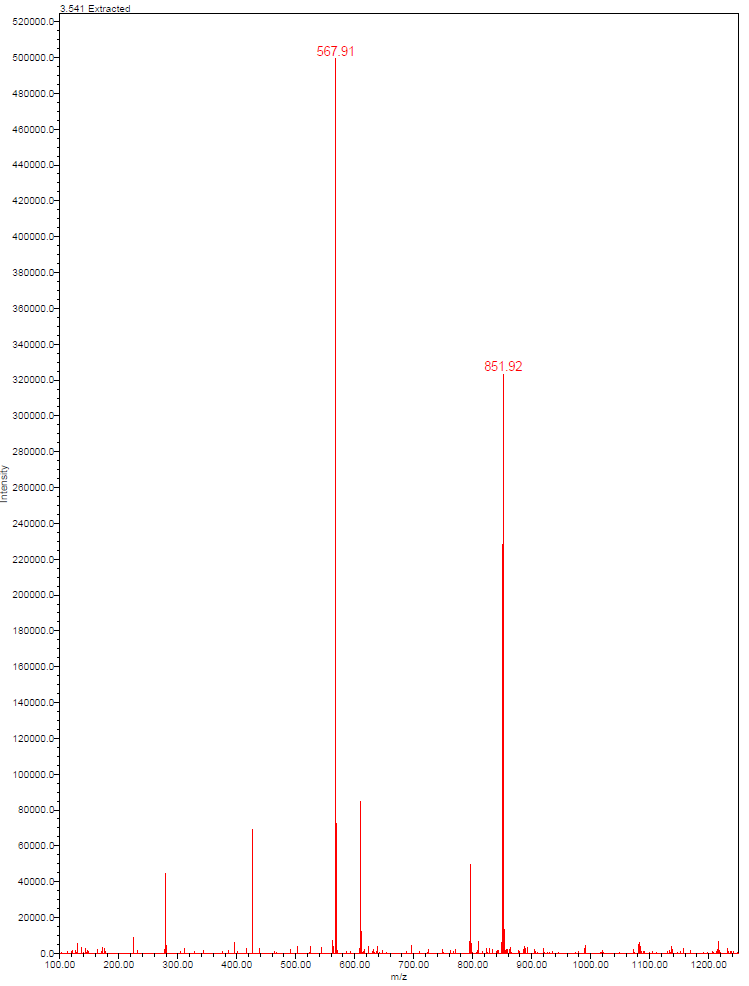


Figure S44: The MS spectrum of Ga-LW01142: calculated [M+2H]^2+^ (m/z) 851.4; found 851.9.

Synthesis of Ga-LW01143

Following general procedures, Ga-LW01143 was obtained in 47% yield after HPLC purification using 26% CH_3_CN and 0.1% TFA in H_2_O (retention time = 12.8 min).


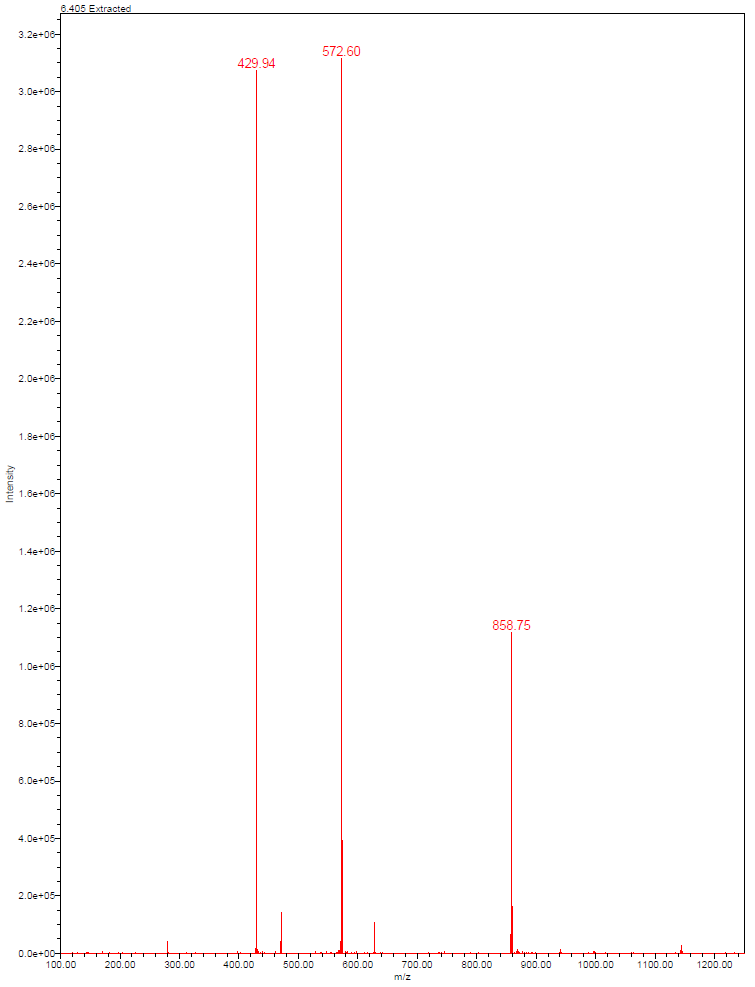


Figure S45: The MS spectrum of Ga-LW01143: calculated [M+2H]^2+^ (m/z) 858.4; found 858.8.

Synthesis of Ga-LW02040

Following general procedures, Ga-LW02040 was obtained in 87% yield after HPLC purification using 25% CH_3_CN and 0.1% TFA in H_2_O (retention time = 16.8 min).


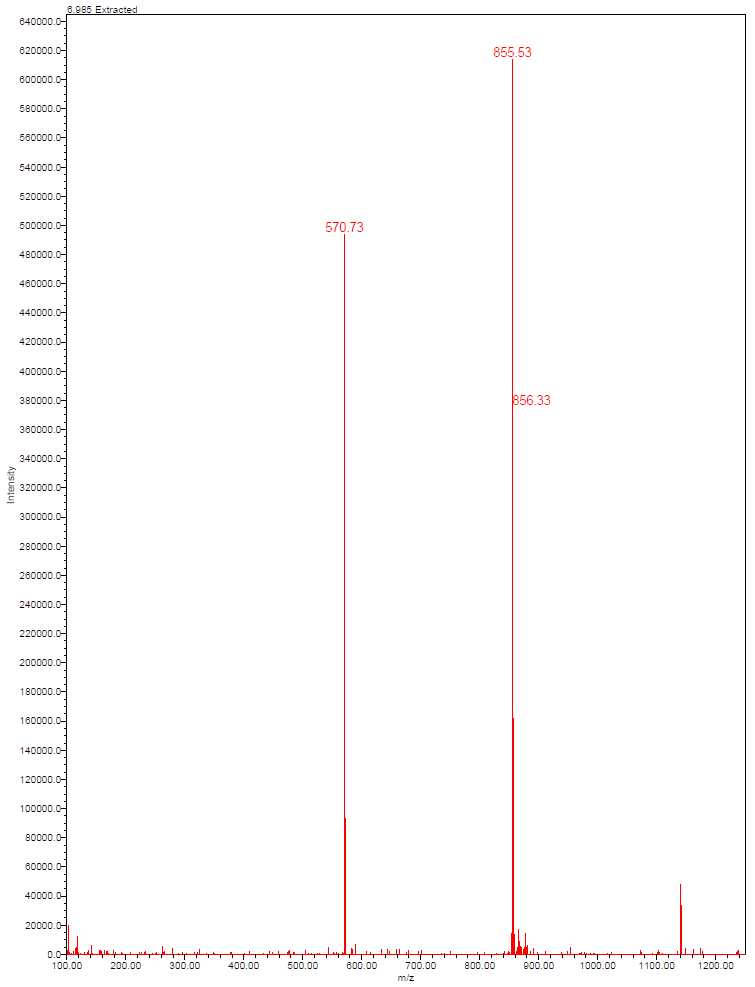


Figure S46: The MS spectrum of Ga-LW02040: calculated [M+2H]^2+^ (m/z) 855.9; found 855.5.

**General procedures for the synthesis of ^68^Ga-labeled compounds**

The radiolabeling experiments were performed following previously published procedures [1-3]. Purified ^68^Ga in 0.5 mL water was added into a 4-mL glass vial preloaded with 700 μL of HEPES buffer (2 M, pH 5.0) and 10 μL precursor solution (1 mM). The radiolabeling reaction was carried out under microwave heating for 1 min before being purified by HPLC using the semi-preparative column. The eluate fraction containing the radiolabeled product was collected, diluted with water (50 mL), and passed through a C18 Sep-Pak cartridge that was pre-washed with ethanol (10 mL) and water (10 mL). The ^68^Ga-labeled product was eluted off the cartridge with ethanol (0.4 mL), and diluted with PBS for imaging and biodistribution. Quality control was performed using the analytical column. The HPLC conditions and retention times are provided in Tables S1. The tracers were obtained in 30-81% decay-corrected radiochemical yields with ≥72.8 GBq/µmol molar activity and >95% radiochemical purity.

Table S4: HPLC conditions for the purification and quality control of ^68^Ga-labeled LW01107, LW01108, LW01110, LW01142, LW02021, and LW02040. FA: formic acid; TFA: trifluoroacetic acid.

| Tracer | HPLC conditions | | Retention time (min) |
| --- | --- | --- | --- |
| [^68^Ga]Ga-LW01107 | Semi-Prep | 16.5% CH_3_CN and 0.1% FA in H_2_O; flow rate 4.5 mL/min | 23.2 |
|  | QC | 20% CH_3_CN and 0.1% FA in H_2_O; flow rate 2 mL/min | 9.0 |
| [^68^Ga]Ga-LW01108 | Semi-Prep | 18% CH_3_CN and 0.1% FA in H_2_O; flow rate 4.5 mL/min | 10.8 |
|  | QC | 21% CH_3_CN and 0.1% FA in H_2_O; flow rate 2 mL/min | 5.8 |
| [^68^Ga]Ga-LW01110 | Semi-Prep | 18% CH_3_CN and 0.1% FA in H_2_O; flow rate 4.5 mL/min | 21.2 |
|  | QC | 22% CH_3_CN and 0.1% FA in H_2_O; flow rate 2.0 mL/min | 8.8 |
| [^68^Ga]Ga-LW01142 | Semi-Prep | 16% CH_3_CN and 0.1% TFA in H_2_O; flow rate 4.5 mL/min | 17.3 |
|  | QC | 19% CH_3_CN and 0.1% FA in H_2_O; flow rate 2.0 mL/min | 11.2 |
| [^68^Ga]Ga-LW02021 | Semi-Prep | 17% CH_3_CN and 0.1% TFA in H_2_O; flow rate 4.5 mL/min | 32.7 |
|  | QC | 21% CH_3_CN and 0.1% FA in H_2_O; flow rate 2.0 mL/min | 12.1 |
| [^68^Ga]Ga-LW02040 | Semi-Prep | 19% CH_3_CN and 0.1% TFA in H_2_O; flow rate 4.5 mL/min | 27.1 |
|  | QC | 22% CH_3_CN and 0.1% FA in H_2_O; flow rate 2.0 mL/min | 6.8 |


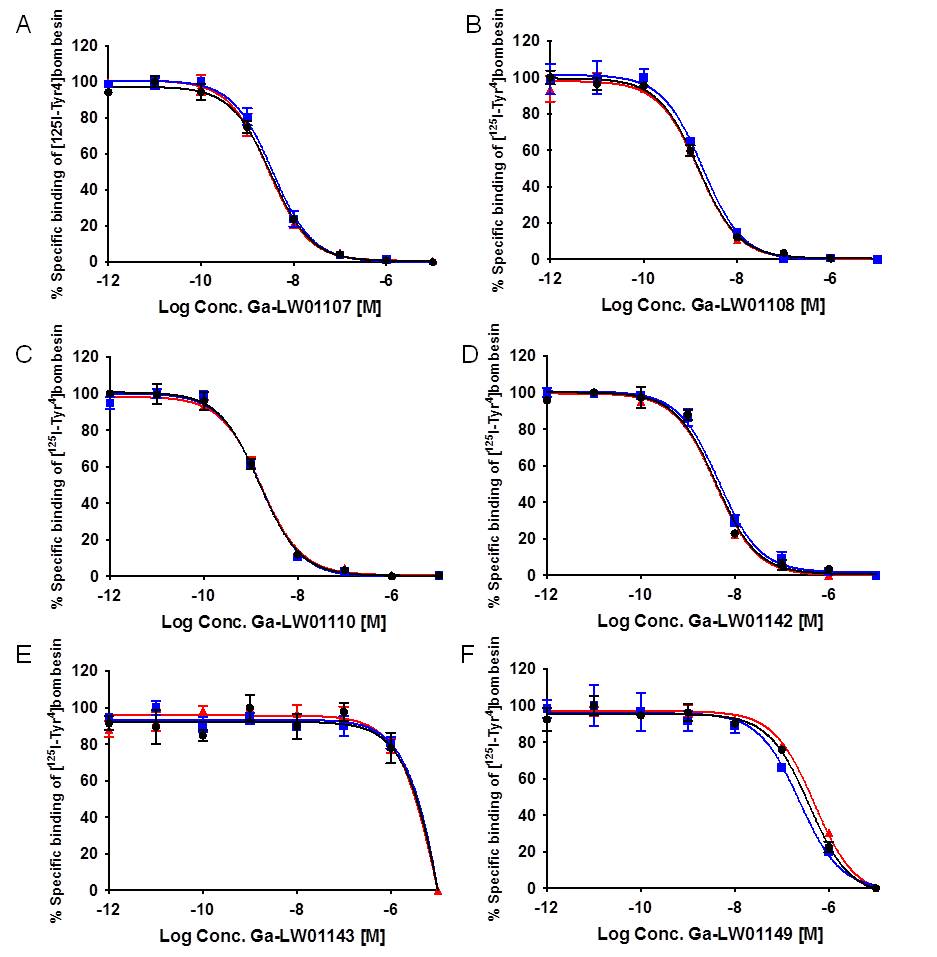


Figure S47: Displacement curves (n = 3) of [^125^I-Tyr^4^]Bombesin by (A) Ga-LW01107, (B) Ga-LW01108, (C) Ga-LW01110, (D) Ga-LW01142, (E) Ga-LW01143, and (F) Ga-LW01149 generated using GRPR-expressing PC-3 cells.


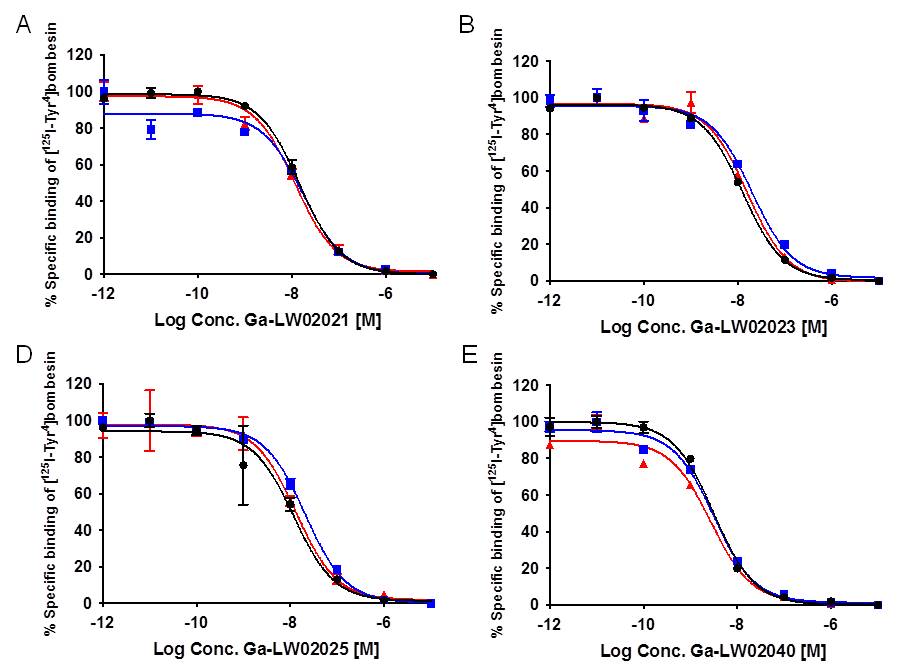


Figure S48: Displacement curves (n = 3) of [^125^I-Tyr^4^]Bombesin by (A) Ga-LW02021, (B) Ga-LW02023, (C) Ga-LW02025, and (D) Ga-LW02040 generated using GRPR-expressing PC-3 cells.


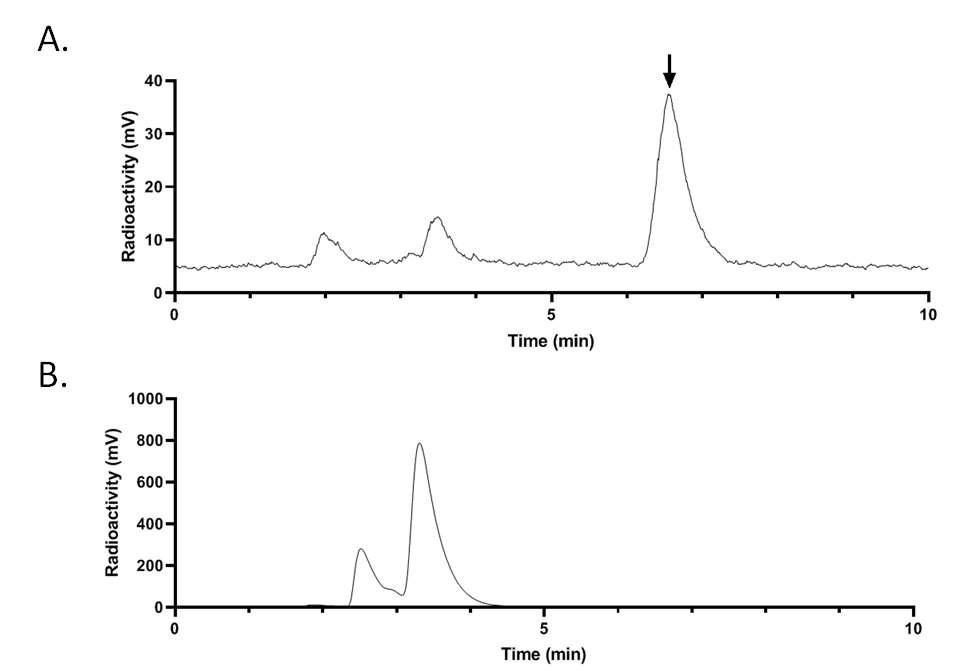


Figure S49: Representative radio-HPLC chromatograms from analysis of intact fraction of [^68^Ga]Ga-LW01107 in mouse (A) plasma and (B) urine samples collected at 15 min post-injection. The peak of intact tracer is pointed by a black arrow.


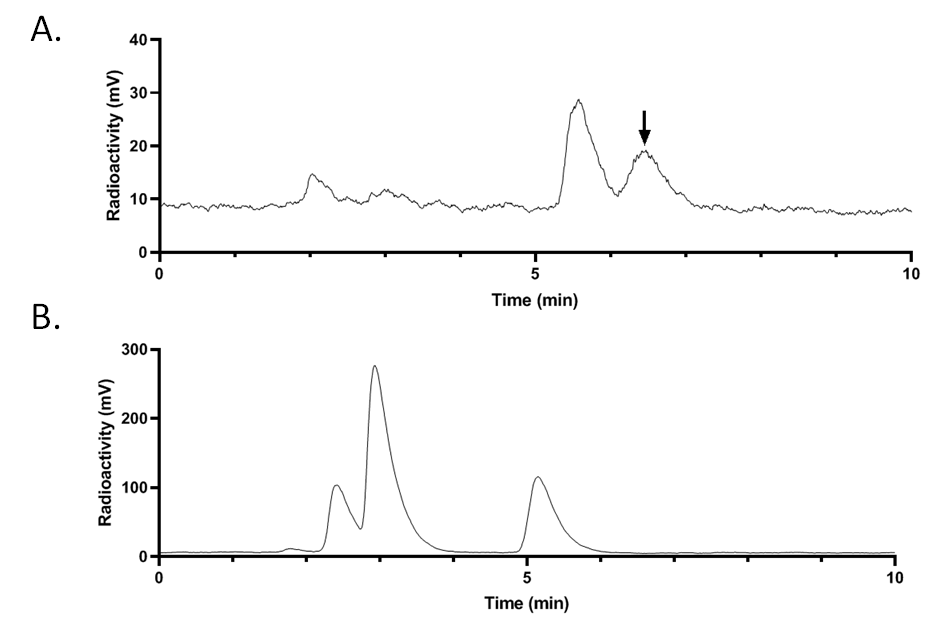


Figure S50: Representative radio-HPLC chromatograms from analysis of intact fraction of [^68^Ga]Ga-LW01108 in mouse (A) plasma and (B) urine samples collected at 15 min post-injection. The peak of intact tracer is pointed by a black arrow.


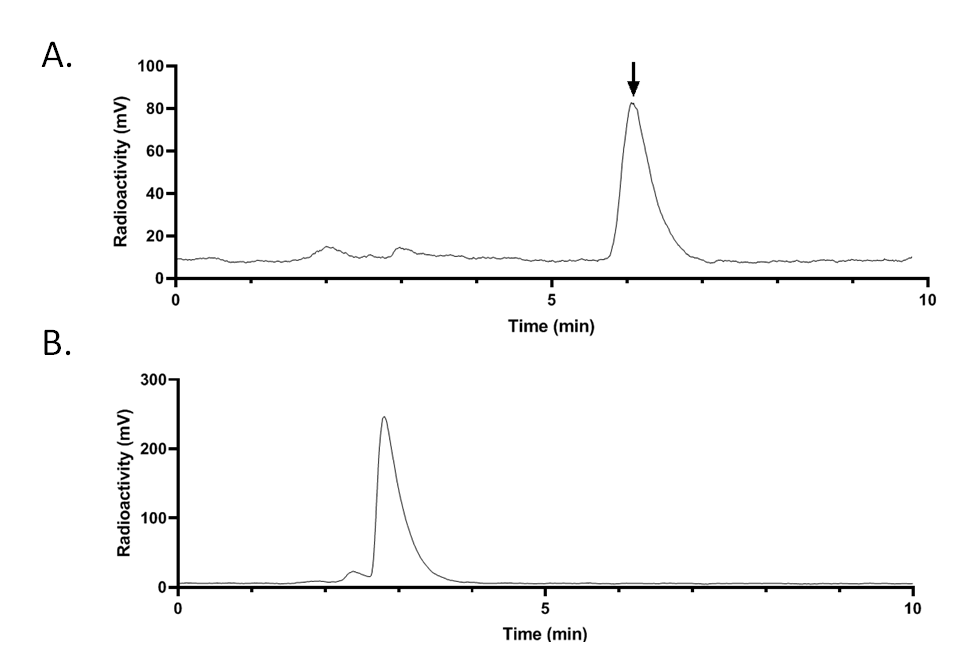


Figure S51: Representative radio-HPLC chromatograms from analysis of intact fraction of [^68^Ga]Ga-LW01110 in mouse (A) plasma and (B) urine samples collected at 15 min post-injection. The peak of intact tracer is pointed by a black arrow.


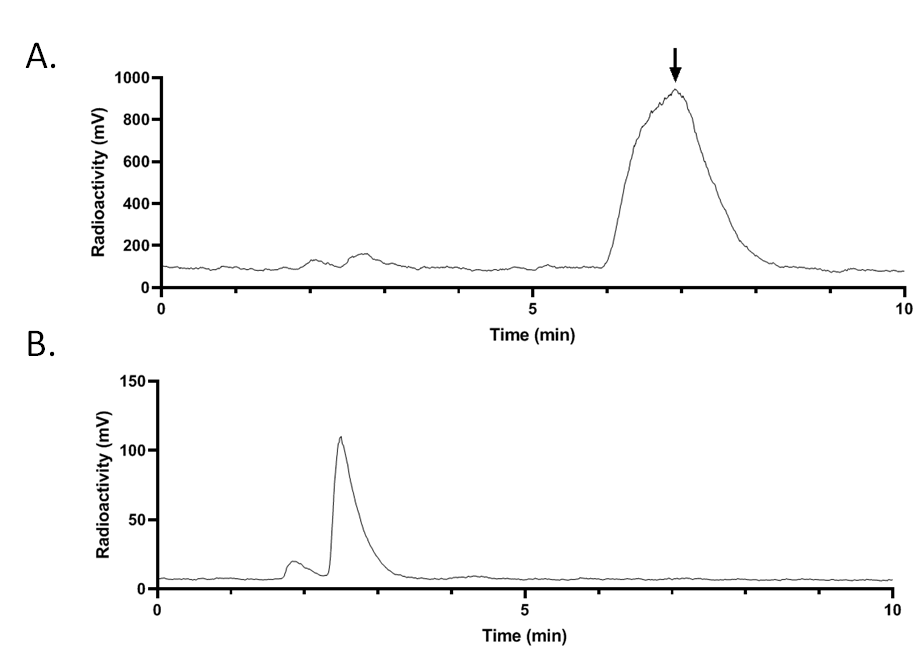


Figure S52: Representative radio-HPLC chromatograms from analysis of intact fraction of [^68^Ga]Ga-LW01142 in mouse (A) plasma and (B) urine samples collected at 15 min post-injection. The peak of intact tracer is pointed by a black arrow.


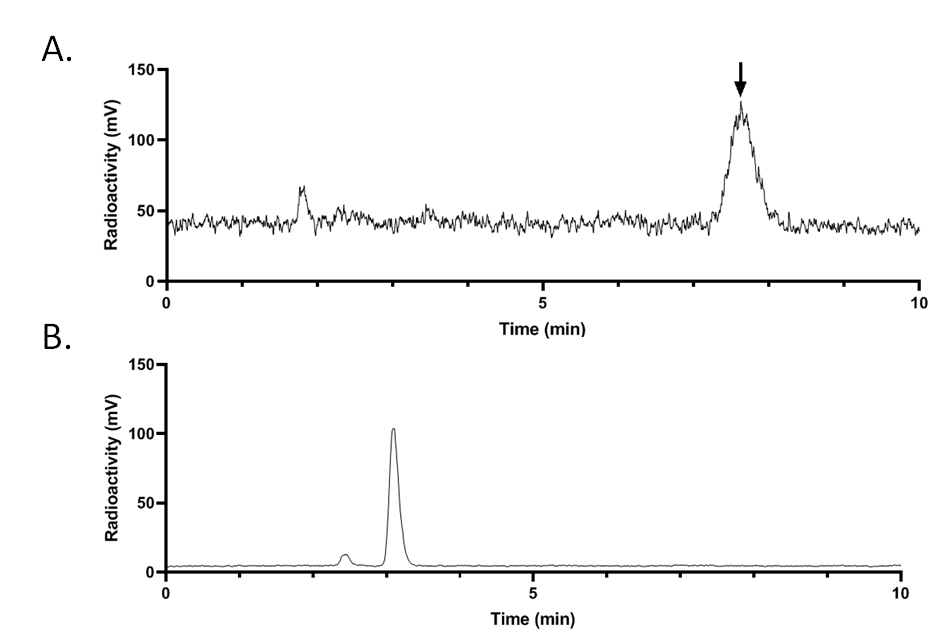


Figure S53: Representative radio-HPLC chromatograms from analysis of intact fraction of [^68^Ga]Ga-LW02040 in mouse (A) plasma and (B) urine samples collected at 15 min post-injection. The peak of intact tracer is pointed by a black arrow.

Table S5: Biodistribution and uptake ratios of ^68^Ga-labeled GRPR-targeted tracers in PC-3 tumor-bearing mice at 1 h post-injection. Data are presented as mean ± SD (n = 4). The data of [^68^Ga]Ga-TacBOMB2, [^68^Ga]Ga-RM2, and [^68^Ga]Ga-AMBA have been reported previously [7, 8], and are included here for comparison.

| Tissue  (%ID/g) | [^68^Ga]Ga-TacBOMB2 | [^68^Ga]Ga-LW01107 | [^68^Ga]Ga-LW01108 | [^68^Ga]Ga-LW01110 | [^68^Ga]Ga-LW01142 | [^68^Ga]Ga-LW02021 | [^68^Ga]Ga-LW02040 | [^68^Ga]Ga- RM2 | [^68^Ga]Ga-AMBA |
| --- | --- | --- | --- | --- | --- | --- | --- | --- | --- |
| Blood | 0.39 ± 0.12 | 0.30 ± 0.04 | 0.59 ± 0.18 | 0.69 ± 0.14 | 6.88 ± 0.29 | 0.53 ± 0.19 | 0.80 ± 0.08 | 0.64 ± 0.10 | 0.58 ± 0.07 |
| Fat | 0.10 ± 0.03 | 0.05 ± 0.01 | 0.06 ± 0.02 | 0.09 ± 0.02 | 0.32 ± 0.08 | 0.05 ± 0.02 | 0.05 ± 0.01 | 0.05 ± 0.03 | 0.08 ± 0.02 |
| Testes | 0.16 ± 0.05 | 0.10 ± 0.02 | 0.16 ± 0.03 | 0.16 ± 0.07 | 1.28 ± 0.19 | 0.36 ± 0.46 | 0.26 ± 0.07 | 0.18 ± 0.03 | 0.20 ± 0.03 |
| Small intestine | 0.60 ± 0.12 | 0.31 ± 0.01 | 2.29 ± 0.54 | 2.11 ± 0.48 | 1.70 ± 0.10 | 0.63 ± 0.12 | 2.42 ± 0.13 | 5.08 ± 1.05 | 8.62 ± 1.00 |
| Large intestine | - | 0.18 ± 0.04 | 1.31 ± 0.33 | 1.27 ± 0.27 | 1.07 ± 0.09 | 0.44 ± 0.18 | 1.90 ± 0.21 | 2.19 ± 0.67 | 4.90 ± 0.91 |
| Spleen | 0.27 ± 0.10 | 0.13 ± 0.02 | 0.40 ± 0.30 | 0.29 ± 0.05 | 0.88 ± 0.09 | 0.84 ± 1.39 | 0.28 ± 0.04 | 0.44 ± 0.26 | 2.49 ± 2.16 |
| **Pancreas** | **1.30 ± 0.14** | **0.39 ± 0.03** | **9.32 ± 1.97** | **8.99 ± 1.54** | **4.40 ± 0.27** | **1.22 ± 0.18** | **11.7 ± 0.47** | **41.9 ± 10.1** | **62.4 ± 4.26** |
| Stomach | 0.54 ± 0.27 | 0.07 ± 0.01 | 0.89 ± 0.18 | 0.94 ± 0.31 | 0.98 ± 0.05 | 0.40 ± 0.27 | 1.47 ± 0.41 | 3.87 ± 2.80 | 2.32 ± 0.60 |
| Liver | 0.29 ± 0.13 | 0.32 ± 0.15 | 0.35 ± 0.19 | 0.41 ± 0.06 | 2.88 ± 0.45 | 0.37 ± 0.14 | 0.71 ± 0.09 | 0.84 ± 0.55 | 0.43 ± 0.05 |
| Adrenal glands | 0.52 ± 0.11 | 0.32 ± 0.19 | 1.51 ± 0.99 | 1.64 ± 0.18 | 2.07 ± 0.27 | 0.38 ± 0.08 | 2.38 ± 1.62 | 3.01 ± 0.91 | 10.0 ± 2.49 |
| Kidneys | 2.43 ± 0.16 | 1.88 ± 0.17 | 2.47 ± 0.66 | 3.26 ± 0.25 | 6.36 ± 0.41 | 2.15 ± 0.29 | 4.00 ± 0.33 | 2.57 ± 0.48 | 5.70 ± 2.45 |
| Heart | 0.14 ± 0.02 | 0.11 ± 0.02 | 0.18 ± 0.04 | 0.23 ± 0.03 | 1.62 ± 0.12 | 0.16 ± 0.05 | 0.27 ± 0.04 | 0.19 ± 0.03 | 0.22 ± 0.02 |
| Lungs | 0.39 ± 0.10 | 0.28 ± 0.04 | 0.49 ± 0.22 | 0.59 ± 0.12 | 4.59 ± 0.81 | 0.49 ± 0.15 | 2.36 ± 1.46 | 0.62 ± 0.26 | 0.62 ± 0.06 |
| **PC-3 tumor** | **5.95 ± 0.50** | **7.05 ± 0.71** | **5.90 ± 0.68** | **16.6 ± 1.60** | **11.4 ± 1.22** | **3.08 ± 0.48** | **12.3 ± 2.14** | **10.5 ± 2.03** | **6.69 ± 1.03** |
| Bone | 0.26 ± 0.11 | 0.06 ± 0.02 | 0.15 ± 0.11 | 0.12 ± 0.00 | 0.47 ± 0.08 | 0.08 ± 0.02 | 0.14 ± 0.01 | 0.11 ± 0.03 | 0.33 ± 0.13 |
| Muscle | 0.13 ± 0.08 | 0.07 ± 0.02 | 0.17 ± 0.11 | 0.14 ± 0.03 | 0.52 ± 0.07 | 0.18 ± 0.04 | 0.18 ± 0.03 | 0.14 ± 0.06 | 0.17 ± 0.01 |
| Brain | 0.03 ± 0.01 | 0.01 ± 0.00 | 0.02 ± 0.00 | 0.03 ± 0.00 | 0.15 ± 0.02 | 0.02 ± 0.01 | 0.03 ± 0.00 | 0.03 ± 0.01 | 0.04 ± 0.01 |
| Tumor/bone | 28.9 ± 18.3 | 126 ± 32.7 | 54.2 ± 32.9 | 134 ± 16.7 | 25.0 ± 4.53 | 38.2 ± 9.17 | 88.6 ± 16.7 | 96.5 ± 27.1 | 22.6 ± 9.13 |
| Tumor/muscle | 57.9 ± 32.8 | 99.8 ± 11.2 | 45.4 ± 19.6 | 119 ± 22.6 | 22.0 ± 2.37 | 18.2 ± 5.02 | 69.9 ± 15.9 | 80.8 ± 27.5 | 39.5 ± 7.46 |
| Tumor/blood | 16.4 ± 5.64 | 23.9 ± 1.80 | 11.0 ± 4.24 | 24.7 ± 4.17 | 1.66 ± 0.18 | 6.51 ± 2.60 | 15.4 ± 3.51 | 16.5 ± 3.06 | 11.7 ± 2.10 |
| Tumor/kidney | 2.46 ± 0.33 | 3.75 ± 0.19 | 2.57 ± 0.96 | 5.10 ± 0.39 | 1.81 ± 0.28 | 1.44 ± 0.23 | 3.08 ± 0.62 | 4.13 ± 0.73 | 1.36 ± 0.73 |
| Tumor/pancreas | 4.64 ± 0.77 | 17.9 ± 1.10 | 0.66 ± 0.17 | 1.87 ± 0.25 | 2.61 ± 0.39 | 2.56 ± 0.44 | 1.04 ± 0.16 | 0.25 ± 0.04 | 0.11 ± 0.01 |

Table S6: Biodistribution (mean ± SD, n = 4) and uptake ratios of ^68^Ga-labeled GRPR-targeted tracers in PC-3 tumor-bearing mice. The mice in the blocked group were co-injected with 100 µg of [D-Phe^6^,Leu-NHEt^13^,des-Met^14^]Bombesin(6-14). The significance of differences between groups (1 h vs 1 h blocked; 1 h vs 3 h): **p* < 0.05; ***p* < 0.01; ****p* < 0.001.

| Tissue  (%ID/g) | [^68^Ga]Ga-LW01110 | |  | [^68^Ga]Ga-LW01142 | | |
| --- | --- | --- | --- | --- | --- | --- |
|  | 1 h | 1 h blocked |  | 1 h | 1 h blocked | 3 h |
| Blood | 0.69 ± 0.14 | 1.57 ± 0.61* |  | 6.88 ± 0.29 | 5.48 ± 0.44* | 2.48 ± 0.61*** |
| Fat | 0.09 ± 0.02 | 0.19 ± 0.06* |  | 0.32 ± 0.08 | 0.47 ± 0.13 | 0.12 ± 0.04** |
| Testes | 0.16 ± 0.07 | 0.42 ± 0.08** |  | 1.28 ± 0.19 | 0.90 ± 0.33 | 0.66 ± 0.20** |
| Small intestine | 2.11 ± 0.48 | 1.25 ± 0.39* |  | 1.70 ± 0.10 | 1.19 ± 0.10*** | 0.76 ± 0.30*** |
| Large intestine | 1.27 ± 0.27 | 0.79 ± 0.21* |  | 1.07 ± 0.09 | 0.71 ± 0.07*** | 0.80 ± 0.21* |
| Spleen | 0.29 ± 0.05 | 0.52 ± 0.07** |  | 0.88 ± 0.09 | 0.82 ± 0.04 | 0.51 ± 0.07*** |
| **Pancreas** | **8.99 ± 1.54** | **5.23 ± 2.42*** |  | **4.40 ± 0.27** | **3.09 ± 0.52**** | **2.14 ± 0.67***** |
| Stomach | 0.94 ± 0.31 | 0.45 ± 0.16* |  | 0.98 ± 0.05 | 0.55 ± 0.16** | 0.32 ± 0.08*** |
| Liver | 0.41 ± 0.06 | 0.82 ± 0.19** |  | 2.88 ± 0.45 | 3.31 ± 0.27 | 1.50 ± 0.31** |
| Adrenal glands | 1.64 ± 0.18 | 1.02 ± 0.20** |  | 2.07 ± 0.27 | 2.47 ± 1.39 | 1.01 ± 0.25*** |
| Kidneys | 3.26 ± 0.25 | 7.09 ± 1.16*** |  | 6.36 ± 0.41 | 7.27 ± 0.66 | 4.88 ± 1.00* |
| Heart | 0.23 ± 0.03 | 0.46 ± 0.12** |  | 1.62 ± 0.12 | 1.39 ± 0.19 | 0.58 ± 0.15*** |
| Lungs | 0.59 ± 0.12 | 1.23 ± 0.37** |  | 4.59 ± 0.81 | 3.80 ± 0.78 | 2.70 ± 0.28** |
| **PC-3 tumor** | **16.6 ± 1.60** | **9.32 ± 1.57***** |  | **11.4 ± 1.22** | **7.85 ± 0.91**** | **15.3 ± 2.45*** |
| Bone | 0.12 ± 0.00 | 0.40 ± 0.35 |  | 0.47 ± 0.08 | 0.39 ± 0.05 | 0.17 ± 0.03*** |
| Muscle | 0.14 ± 0.03 | 0.73 ± 0.73 |  | 0.52 ± 0.07 | 0.64 ± 0.16 | 0.18 ± 0.05*** |
| Brain | 0.03 ± 0.00 | 0.06 ± 0.02* |  | 0.15 ± 0.02 | 0.10 ± 0.01** | 0.06 ± 0.01*** |
| Tumor/bone | 134 ± 16.7 | 35.5 ± 18.7*** |  | 25.0 ± 4.53 | 20.7 ± 4.77 | 91.6 ± 12.2*** |
| Tumor/muscle | 119 ± 22.6 | 21.4 ± 12.2*** |  | 22.0 ± 2.37 | 13.4 ± 5.41* | 86.6 ± 25.1** |
| Tumor/blood | 24.7 ± 4.17 | 6.62 ± 2.48*** |  | 1.66 ± 0.18 | 1.43 ± 0.16 | 6.40 ± 1.70*** |
| Tumor/kidney | 5.10 ± 0.39 | 1.32 ± 0.15*** |  | 1.81 ± 0.28 | 1.09 ± 0.17** | 3.17 ± 0.46** |
| Tumor/pancreas | 1.87 ± 0.25 | 1.99 ± 0.62 |  | 2.61 ± 0.39 | 2.57 ± 0.30 | 7.36 ± 1.17*** |
|  |  |  |  |  |  |  |

**References**

1. Lin K-S, Pan J, Amouroux G, Turashvili G, Mesak F, Hundal-Jabal N, et al. In vivo radioimaging of bradykinin receptor B1, a widely overexpressed molecule in human cancer. Cancer Res. 2015;75:387-93.

2. Amouroux G, Pan J, Jenni S, Zhang C, Zhang Z, Hundal-Jabal N, et al. Imaging bradykinin B1 receptor with ^68^Ga-labeled [des-Arg^10^] Kallidin derivatives: effect of the linker on biodistribution and tumor uptake. Mol. Pharmaceutics. 2015;12:2879-88.

3. Lin K-S, Amouroux G, Pan J, Zhang Z, Jenni S, Lau J, et al. Comparative studies of three ^68^Ga-labeled [Des-Arg^10^] kallidin derivatives for imaging bradykinin B1 receptor expression with PET. J. Nucl. Med. 2015;56:622-7.

4. Lau J, Rousseau E, Zhang Z, Uribe CF, Kuo H-T, Zeisler J, et al. Positron emission tomography imaging of the gastrin-releasing peptide receptor with a novel bombesin analogue. ACS Omega. 2019;4:1470-8.

5. Bratanovic IJ, Zhang C, Zhang Z, Kuo HT, Colpo N, Zeisler J, et al. A Radiotracer for Molecular Imaging and Therapy of Gastrin-Releasing Peptide Receptor-Positive Prostate Cancer. J. Nucl. Med. 2022;63:424-30.

6. Zhang C, Pan J, Lin K-S, Dude I, Lau J, Zeisler J, et al. Targeting the neuropeptide Y1 receptor for cancer imaging by positron emission tomography using novel truncated peptides. Mol. Pharmaceutics. 2016;13:3657-64.

7. Wang L, Zhang Z, Merkens H, Zeisler J, Zhang C, Roxin A, et al. ^68^Ga-Labeled [Leu^13^ψThz^14^] Bombesin (7–14) Derivatives: Promising GRPR-Targeting PET Tracers with Low Pancreas Uptake. Molecules. 2022;27:3777.

8. Wang L, Bratanovic IJ, Zhang Z, Kuo H-T, Merkens H, Zeisler J, et al. ^68^Ga-Labeled [Thz^14^] Bombesin (7–14) Analogs: Promising GRPR-Targeting Agonist PET Tracers with Low Pancreas Uptake. Molecules. 2023;28:1977.
